# Photothermal-driven multifunctional injectable hydrogel platform for promoting diabetic bone defect repair through synergistic immunomodulation and bone homeostasis

**DOI:** 10.7150/thno.130240

**Published:** 2026-03-25

**Authors:** Yufan Zhu, Huifan Liu, Zhiqiang Yang, Yaxing He, Ping Wu, Lin Cai, Lufeng Yao, Xiaobin Zhu, Minhao Wu

**Affiliations:** 1 Department of Radiation and Medical Oncology, Zhongnan Hospital, Wuhan University, Wuhan, China.; 2 Department of Spine Surgery and Musculoskeletal Tumor, Zhongnan Hospital of Wuhan University, 168 Donghu Street, Wuchang District, Wuhan 430071 Hubei, China.; 3 Department of Anesthesiology, Research Centre of Anesthesiology and Critical Care Medicine, Zhongnan Hospital of Wuhan University, Wuhan, Hubei, China.; 4 State Key Laboratory of Macromolecular Drugs and Large-scale Preparation, School of Pharmaceutical Science, Wenzhou Medical University, Wenzhou 325035, China.; 5 Department of foot and ankle Surgery, Ningbo No.6 Hospital, Ningbo, 315040, China.; 6 Ningbo Clinical Research Center for Orthopedics, Sports Medicine & Rehabilitation, Ningbo, Zhejiang, China.

**Keywords:** diabetic bone defects, immune microenvironment, mild heat stimulation, bone homeostasis, spatial-temporal intelligent release

## Abstract

**Background:**

Excessive inflammation and exacerbated oxidative stress are significant hallmarks of the diabetic bone microenvironment, which give rise to dysregulated immune reactions and impaired bone homeostasis, thereby hindering bone defect healing and increasing the incidence of bone nonunion.

**Methods:**

A biodegradable photothermal hybrid (SC/MTZ) was developed through the *in situ* self-assembly of zeolitic imidazolate framework-8 (ZIF-8) nanoparticles on tannic acid (TA)-functionalized Ti_3_C_2_T_x_ MXene nanosheets, which were then integrated into a methacrylated silk fibroin/carboxymethyl chitosan methacryloyl matrix. The integration of *in situ* photopolymerization and chelation coordination for double crosslinking, along with the incorporation of heterojunction MXene@TA/ZIF-8 (MTZ) nanosheets, enhances the physicochemical properties and biological activity of the hydrogels, providing optimal mechanical support and prolonged retention at the defect site.

**Results:**

The hydrogel platform demonstrated outstanding antibacterial properties and effectively reprogrammed macrophages from the proinflammatory M1 phenotype to the anti-inflammatory M2 phenotype. This was achieved through the combined effects of localized mild hyperthermia and stimuli-responsive release of bioactive agents (TA and Zn^2+^), which also enhanced mitochondrial function and inhibited RANKL-induced osteoclast formation and bone resorption. *In situ* injection of the photoactivated SC/MTZ hydrogel markedly accelerated cranial defect healing in diabetic rats by synergistically enhancing immune homeostasis, osteogenesis, and angiogenesis while suppressing osteoclast activity.

**Conclusions:**

In summary, this study proposes an innovative method for developing multifunctional photothermal nanosheet-encapsulated hybrid hydrogels aimed at effectively managing diabetic bone defects.

## Introduction

With the increasing prevalence of diabetes and the significant decline in bone metabolism among elderly individuals, the treatment of diabetic bone defects has become a critical clinical issue in orthopedics, particularly in the context of an aging population 1. Existing clinical evidence suggests that patients with compromised metabolic patterns, particularly pathological conditions such as diabetes mellitus, encounter substantial challenges in terms of tissue repair and regeneration compared with healthy individuals, potentially leading to higher disability and mortality rates 2. This dilemma is linked to an imbalanced regenerative microenvironment due to inflammation, oxidative stress, bacterial infection, inadequate neovascularization, and disrupted bone homeostasis, which together constitute a harmful cascade reaction that hinders the healing of bone defects 3, 4. In the pathological microenvironment of diabetes mellitus, macrophages are one of the most critical cells involved in bone repair. They tend to differentiate into the proinflammatory M1 phenotype and are prevented from transitioning into the anti-inflammatory M2 phenotype 5. This situation leads to an overproduction of proinflammatory mediators and reactive oxygen species (ROS), sustaining a prolonged inflammatory state that impairs the natural bone healing process 6. Growing evidence suggests that increased oxidative stress and subsequent ROS accumulation in the microenvironment are key factors impairing the regenerative potential of endogenous cells during diabetic bone repair 7. Excessive ROS production in diabetes patients surpasses the cellular antioxidant capacity, leading to mitochondrial dysfunction, DNA and protein damage, extensive osteoblast apoptosis, and inhibited osteogenic differentiation of bone marrow mesenchymal stem cells (BMSCs). Besides, disrupted immune balance and excessive ROS accumulation, along with wound exposure, increase susceptibility to bacterial and microbial infections, including biofilm formation, which may delay bone defect healing 8. Increased proinflammatory cytokines such as TNF-α and IL-6, along with persistent NF-κB signaling activation, are considered key factors disrupting bone homeostasis between osteoblast-driven formation and osteoclast-driven resorption^6^. Unlike proinflammatory M1 macrophages, M2 macrophages promote inflammation resolution, vascularization, and tissue regeneration by releasing anti-inflammatory cytokines such as IL-4 and IL-10, along with angiogenic and pro-differentiating factors such as VEGF, TGF-β, and BMP-2. Additionally, an M2-dominant immune microenvironment has proven favorable for the integration of implanted materials with surrounding host tissues 9. Recent studies have shown that M2 macrophage-derived anti-inflammatory cytokines, including TGF-β and IL-10, inhibit osteoclast maturation by blocking the RANKL/RANK interaction and promoting osteogenic differentiation, thus collaboratively maintaining bone homeostasis 10. Consequently, the development of a comprehensive strategy that leverages multidimensional synergistic effects, such as antioxidation, antibacterial action, M1/M2 phenotype transformation regulation, and the enhancement of osteogenesis and angiogenesis, is essential for effectively treating diabetic bone defects.

Recent advancements in bone graft development have focused on incorporating diverse material-mediated biophysical cues, including morphology, light, electrical, mechanical, and magnetic signals, and heat. These innovations provide valuable insights into coordinating inflammation and tissue regeneration during bone repair 11. Mild heating (41-43 °C) via near-infrared (NIR)-mediated photothermal therapy (PTT) serves as a crucial biophysical regulator, affecting biological processes such as inflammation, immune reactions, and bone regeneration 12. Numerous clinical studies have shown that mild heat stimulation effectively promotes fracture healing, and local mild hyperthermia therapy is advised as a supplementary treatment for patients with traumatic bone ischemia and nonunion 13. In the context of complex conditions such as diabetes mellitus, mild hyperthermia-assisted nanomaterials can alleviate alveolar bone resorption and promote bone healing in diabetic periodontitis by attenuating inflammation, inhibiting apoptosis, eliminating ROS and bacteria, and correcting mitochondrial dysfunction 14, 15. Mild heat stimulates osteogenic differentiation and bone formation by upregulating the expression of osteogenesis-related markers and heat shock proteins (HSPs). Owing to its minimal invasiveness, high spatiotemporal control, specificity, and repeatability, mild PTT has garnered significant recognition, showing significant potential in regulating cellular functions and tissue homeostasis 16. Two-dimensional (2D) Ti_3_C_2_T_x_ MXene nanosheets, which are responsive to photo-stimuli, have been extensively studied in the biomedical field because of their excellent photothermal conversion capability, biocompatibility, and natural osteoinductivity 17. MXene exhibits superior catalase-like activity, effectively scavenging reactive oxygen species such as H_2_O_2_ and •OH, thereby assisting in maintaining the cellular redox balance and controlling inflammation 18. However, their poor oxidation stability, susceptibility to aggregation and lack of functionalities present great challenges for their biological applications, especially in the context of prolonged inflammation and excessive ROS. Our earlier study highlighted the significant potential of 2D Ti_3_C_2_T_x_ MXene nanosheets in promoting osteogenic differentiation and bone regeneration. However, these materials may experience reduced stability and antioxidant efficiency under adverse conditions, such as redox imbalance or acidic environments. In addition, a single application of an MXene-based material fails to completely suppress deteriorated inflammation and oxidative stress under diabetic conditions, thus limiting its therapeutic effect and clinical applications 19. Another important point to consider is that the therapeutic efficacy of mild PTT is also limited, and achieving satisfactory outcomes for bone regeneration via mild PTT alone is challenging because of the complex physiological microenvironment present in diabetic bone defect sites. Thus, it is imperative to enhance the functionalities of MXene nanosheets to improve their physicochemical and biological performance 18. Zeolitic imidazolate framework-8 (ZIF-8), a metal-organic framework (MOF) known for its pH sensitivity, intelligently responds to the post-traumatic slightly acidic environment (pH~6.5) by continuously releasing Zn^2+^, thereby exhibiting strong antibacterial, anti-inflammatory, and pro-osteo-/angiogenic properties 20. Zn, the second most prevalent micronutrient in the body, supports bone tissue growth and mineralization. Furthermore, Zn plays a significant role in the inhibition of osteoclastic activity 21. Research has demonstrated the osteogenic and immunomodulatory properties of ZIF-8 nanoparticles and their nanocomposites. These materials facilitate the transformation of proinflammatory M1 macrophages to anti-inflammatory M2 macrophages, thereby enhancing osteogenic differentiation and bone regeneration 22. ZIF-8 holds significant potential for diverse applications in bone repair. Despite their bioactivity, these nanoparticles suffer from low stability and rapid degradation. Using pure ZIF-8 material as a drug delivery system poses challenges in sustaining the regenerative environment necessary for bone defect healing. Moreover, most MOFs are hydrophobic because of the hydrophobic groups on their organic ligands, which results in their aggregation and sedimentation under physiological conditions, severely restricting their application in the field of biomedical engineering 23.

Polyphenols, including epigallocatechin 3-gallate (EGCG), tannic acid (TA), pyrogallol, gallic acid, and dopamine (DA), are extensively used for material surface functionalization due to their excellent biocompatibility, biodegradability, adhesive properties, and mild synthesis conditions 24. Polyphenols exhibit significant ROS scavenging and antibacterial properties and are characterized by extensive free radical neutralization and stability under physiological conditions, indicating their potential for healing chronic diabetic wounds and infected bone defects 7, 25. TA, a plant-derived polyphenol rich in hydroxyl groups, can establish multiple hydrogen bonds with MXene, thereby increasing its oxidation stability and biological functions through straightforward compounding 26. TA, a primary component of coatings, has received approval from the United States Food and Drug Administration (FDA) 27. The polyphenol-mediated *in situ* modification approach effectively enhances the stability and biological properties of MXene nanosheets, improving their ROS scavenging and anti-inflammatory capabilities. A separate study demonstrated that combining mild hyperthermia with anti-inflammatory and antioxidant activities effectively alleviates oxidative stress and promotes M2 macrophage polarization, thus diminishing inflammatory responses and fostering an environment conducive to bone repair 28. Consequently, TA-modified MXene (MXene@TA) nanosheets are anticipated to facilitate tissue repair and bone regeneration in inflammatory environments by enhancing ROS elimination and immune regulation through mild PTT. TA acts as an optimal biological growth template and stabilizing agent, offering numerous adhesion sites for the imidazolate ligand. This facilitates the formation of dimensionally stable ZIF-8 nanosheets with Zn^2+^ ions via classical coordination chemistry. We hypothesized that the formation of a heterojunction material via the growth of ZIF-8 nanoparticles on a polyphenol-mediated MXene (MXene@TA) could combine the benefits of photothermal agents, bioactive agents, and natural antioxidants. This integration may effectively eliminate pathogenic bacteria, scavenge reactive oxygen species (ROS), alleviate oxidative stress, and modulate local immune responses.

Given the challenges of local administration at bone defect sites, an advanced treatment platform for loading and delivering therapeutic agents is needed to maximize nanomaterial efficacy and provide a biocompatible microenvironment favorable for bone regeneration. Moreover, for nanoscale bioactive materials, ensuring their long-term retention at the target site after *in situ* injection while also meeting the structural support and mechanical strength required for bone repair is difficult 29. Hydrogels, characterized by their hydrophilic, porous, and biocompatible 3D polymer networks, have become promising carriers for functional substances such as growth factors, drugs, cells, and bioactive materials. In recent years, bioactive hydrogels have attracted significant interest from researchers due to their potential for tissue engineering applications. Photo-crosslinking hydrogels offer substantial benefits for bone regeneration, such as biocompatibility, biosafety, biodegradability, and excellent injectability, which enable them to conform to diverse defect shapes 30. These characteristics increase the contact area between the implants and tissue, thereby increasing the probability of irregular bone defect repair. Despite some encouraging progress, most currently available photo-crosslinking hydrogel systems possess weak mechanical properties and rapid degradation, which compromise their structural stability and bioactivity, thereby limiting the use of these hydrogels in long-term bone repair. Despite the enhancement of the physical properties and bioactivity of photo-crosslinked hydrogels through modification strategies like adding functional nanoparticles, challenges such as uneven inorganic particle dispersion and aggregation within the polymer matrix remain significant obstacles for biomedical applications of hydrogel implants 31. Research has shown that polyphenol modification strategies enhance nanofiller hydrophilicity, promoting their even distribution in hydrogel networks 32, 33. Moreover, polyphenol-mediated nanofillers endow these hydrogels with enhanced mechanical performance and provide excellent degradation protection through the formation of multiple covalent and noncovalent interactions (such as electrostatic interactions, hydrogen bonding, and π-π stacking). More importantly, polyphenol-functionalized nanofiller-encapsulated hydrogels alleviate oxidative stress and the inflammatory response by scavenging ROS in the diabetic pathological microenvironment and downregulating proinflammatory cytokines 34. These findings suggest that MTZ heterojunction nanosheets, which are composed of ZIF-8-decorated MXene@TA, can be incorporated into injectable and photocurable hydrogels. This integration synergizes the benefits of both nanosheets and hydrogels, potentially enhancing multimodal therapeutic capabilities and offering innovative solutions for diabetic bone defect repair.

**Scheme 1:**
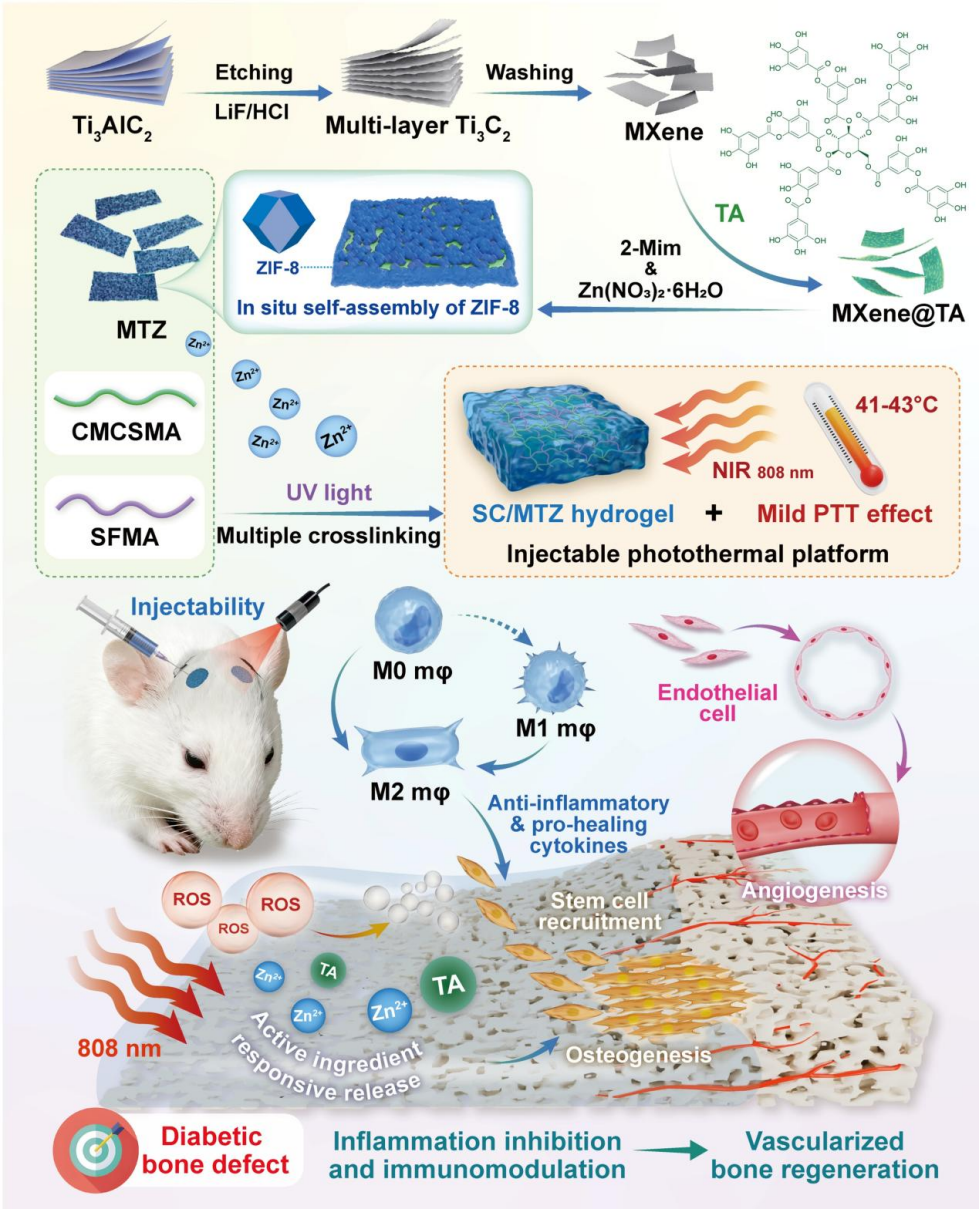
Schematic diagram of the design and application of a mild photothermal-driven multifunctional injectable hydrogel platform to restore the inflammatory microenvironment and promote the regeneration of diabetic bone defects. The programmed SC/MTZ hydrogel achieves intelligent, on-demand release of Zn^2+^ ions and TA in response to the combined effects of NIR irradiation (external stimuli) and weakly acidic diabetic conditions (internal stimuli), thereby enabling control of bacterial infection and coordination of improvements in the bone microenvironment. The spatiotemporal local mild hyperthermia-assisted SC/MTZ hydrogel platform enhanced the immune microenvironment by increasing anti-inflammatory cytokines in macrophages and inhibiting osteoclastogenesis. It also directly promoted BMSC osteogenic differentiation and HUVEC angiogenesis, supporting vascularized bone regeneration.

In this study, we developed a multifunctional injectable nanocomposite hydrogel (SC/MTZ) to test our hypothesis. This platform aims to prevent bacterial infection, alleviate oxidative stress, regulate local inflammation, restore immune balance, and promote osteo-/angiogenesis. These functions collectively increase nutrient, oxygen, and cell supplies at the defect site, thereby facilitating the healing of diabetic bone defects. **Scheme 1** demonstrates that silk fibroin (SF) and carboxymethyl chitosan (CMCS), both natural biological macromolecules, are first modified with methacryloyl groups to create the basic structure of the hydrogel. The resulting SF/CMCS (SC) is a photocurable hydrogel with a bone ECM-like microstructure, developed in our previous study with minor adjustments 22, 35. To address the intricate pathological changes in bone injuries caused by diabetes mellitus, a multifunctional heterojunction nanoplatform (MTZ) was developed. This platform, which is designed to scavenge ROS, modulate macrophage polarization, and enhance osteogenesis, was synthesized using MXene@TA nanosheets as a biological template, facilitating the *in situ* growth of nanoscale ZIF-8 within the nanosheets. After TA treatment, the catechols of the MXene serve as anchoring sites, facilitating the *in situ* growth of ZIF-8 via robust electrostatic adsorption and chelation. Owing to the TA modification and sequential *in situ* growth of the ZIF-8 nanoparticles, the resulting MTZ heterojunctions exhibited excellent structural stability, antioxidant effects, anti-inflammatory ability, and pro-osteogenic capacity while retaining their excellent photothermal properties. The MTZ heterojunctions were subsequently efficiently integrated with injectable SC hydrogels through a double-dynamic network involving covalent bonds (derived from double bond radical polymerization of the SC skeleton) and metal coordination bonds (originating from the Zn^2+^ in ZIF-8 and the carboxyl and amino groups in CMCS). The engineered MTZ nanosheets with strong interfacial compatibility were uniformly embedded within the hydrogel matrix, conferring improved mechanical properties and photothermal conversion capability. This method may enhance the efficacy of functionalized MTZ nanosheets in supporting cell survival, activating osteoblasts and macrophages, and reducing oxidative stress in the bone defect microenvironment. RNA sequencing (RNA-seq) analysis was conducted to investigate the mechanisms contributing to the pathological inflammatory environment. The MTZ-encapsulated hydrogel exhibited strong and potent antibacterial properties due to the inherent biological activity of CMCS, MXene, TA, and ZIF-8. *In vitro* and *in vivo* experiments demonstrated that the multifunctional hydrogel system, when exposed to mild NIR irradiation, exhibited synergistic therapeutic effects. These include preventing bacterial invasion to inhibit infection and restore mitochondrial function, modulating osteoclast and osteoblast activity to maintain bone homeostasis, and promoting M2 macrophage polarization to regulate inflammatory and regenerative factors, thereby enhancing the osteoimmune microenvironment for improved bone regeneration in diabetic conditions. The mild photothermal-assisted SC/MTZ hydrogel system shows significant potential for managing diabetic bone defects because of its injectability, ability to induce mild hyperthermia, effective modulation of the local immune microenvironment, and synergistic therapeutic effects **(Scheme 1)**. It also offers promising prospects for repairing other inflammation-related tissues.

## Materials and Methods

### Materials

All chemicals and reagents used in this study were of analytical grade and utilized as received without further purification. The specific materials and their corresponding commercial suppliers are detailed as follows: cocoons (Huzhou Silk Co., Ltd., Zhejiang, China); carboxymethyl chitosan, tannic acid (TA), zinc nitrate hexahydrate (ZnNO_3_⋅6H_2_O), sodium hydroxide (NaOH), layered ternary carbide (Ti_3_AlC_2_) powder, hydrochloric acid (HCl), methacrylic anhydride, glycidyl methacrylate (GMA), 4′-6-diamidino-2-phenylindole (DAPI), 2-methylimidazole, lithium phenyl (2,4,6-trimethylbenzoyl) phosphinate (LAP), and Triton X-100 (Sigma-Aldrich, Shanghai, China); DPPH, lithium fluoride (LiF), lithium bromide (LiBr), ABTS, and hydrogen peroxide (H_2_O_2_) (Aladdin Chemistry, Shanghai, China). For biological assessments, lipopolysaccharide (LPS) and streptozotocin (STZ) were supplied by MedChemExpress (NJ, USA). Various cellular probes and assay kits, including the Live/dead cell staining kit (Calcein-AM/EthD-1), DHE, and DCFH-DA, were acquired from BestBio Biotechnologies (Shanghai, China). Furthermore, CCK-8, TRIzol RNA extraction kit, RIPA lysis buffer, BCIP/NBT ALP color development kit, Hoechst 33342, JC-1 staining assay kit, and MDA assay kit were sourced from Beyotime Biotechnology (Shanghai, China). Alizarin red S (ARS) and Von Kossa staining kits were procured from Solarbio Co., Ltd. (Beijing, China). Recombinant M-CSF and RANKL were provided by R&D Systems. Bovine bone slices were derived from Thousand Sunrise Bio-Technology Co., Ltd. (Shanghai, China). Any other unspecified standard chemical reagents were purchased from Macklin Biochemical Technology Co., Ltd. (Shanghai, China).

### Preparation and characterization of SFMA and CMCSMA

Both SFMA and CMCSMA were synthesized according to previously described methods 36, 37. To extract SF, silkworm cocoons were cut into strips and boiled in Na_2_CO_3_ solution (0.02 M) for 1 h to remove the sericin layer. After washing three times with deionized (DI) water, the degummed silk (2 g) was air-dried at 37 °C, dissolved in 10 mL of LiBr solution (9.3 M), and then stirred continuously for 4 h at 60 °C. After dissolution, 350 mM glycidyl methacrylate (GMA) was added to the mixture, which was stirred at 60 °C for 3 h. Next, the resulting mixture was dialyzed against DI water for 5 days. For the synthesis of CMCSMA, CMCS (10 g) was dissolved in 100 mL of DI water and stirred at 60 °C for 1 h until it was completely dissolved. Subsequently, methacrylic anhydride (8 mL) was added to the mixture, which was stirred continuously at 60 °C for 3 h. To halt the reaction, preheated PBS was added, and the mixture was stirred for 15 min. Subsequently, the resulting product was dialyzed against DI water for 7 days at 4 °C, followed by lyophilization to obtain purified CMCSMA samples. The lyophilized SFMA and CMCSMA products were stored at -80 °C for subsequent experiments.

### Preparation and characterization of MTZ

MXene was synthesized by etching the Ti_3_AlC_2_ phase using established protocols 38, 39. Initially, 1 g of Ti_3_AlC_2_ powder was slowly added to 10 mL of LiF/HCl etching solution and stirred continuously for 24 h. Afterwards, the obtained mixture was centrifuged and repeatedly rinsed with DI water. Finally, the precipitates were collected to yield MXene. For surface modification, TA (10 mg) was added to 10 mL of DI water and stirred, followed by the incorporation of 100 mg of MXene nanosheets. The mixture was then sonicated for an additional 5 min and stirred for 8 h. After the reaction, the precipitate was collected and lyophilized to yield TA-functionalized MXene (MXene@TA) nanosheets. After the successful preparation of the MXene@TA nanosheets, ZIF-8 was synthesized *in situ* on the MXene surface to fabricate heterogeneous MTZ nanosheets, following previously described methods 40. Briefly, 100 mg of MXene@TA nanosheets were dispersed in 10 mL of ethanol and stirred with 3.24 g of 2-methylimidazole (2-Mim) at room temperature for 8 h to obtain a uniform suspension. Subsequently, 0.1833 g of Zn(NO_3_)_2_·6H_2_O was added to the mixture, which was stirred vigorously for 6 h. Finally, the products were collected by centrifugation and lyophilized to obtain MTZ nanosheets. Moreover, pure ZIF-8 nanoparticles were prepared for comparison via the same protocol without the incorporation of MXene@TA nanosheets.

Field-emission scanning electron microscopy (FE-SEM, Zeiss, SIGMA, Germany) was used to characterize the morphology and elemental distribution of the samples. Atomic force microscopy (AFM; Bruker Dimension FastScan, Germany) was used to evaluate the thickness of each sample. X-ray diffraction (XRD, Ultima4, Japan), Fourier transform infrared spectroscopy (FTIR, SP400, Norwalk, CT, USA), and X-ray photoelectron spectroscopy (XPS, ESCALAB 250XI, Thermo Scientific, New York) were used to assess the crystal structure, chemical composition, and element states. A Zetasizer Nano system (Malvern Instruments, UK) was utilized to record both the surface charge (zeta potential) and the hydrodynamic size distribution of the prepared nanoparticles. The optical absorption properties of all formulations were acquired using a Shimadzu UV-1800 spectrophotometer (Japan). To assess their thermal degradation behavior, the samples were subjected to a Netzsch STA 2500 Regulus analyzer (Germany) in a continuous nitrogen flow. The *in vitro* photothermal conversion capabilities were monitored by irradiating the samples with a continuous-wave NIR laser (808 nm, KS-810F-8000, Kai Site, China). Temperature variations were simultaneously captured utilizing an infrared thermal camera (FLIR Systems, Inc., Wilsonville, OR). Specifically, the samples (50 μg/mL) were exposed to NIR irradiation (1.5 W/cm^2^, 5 min). During irradiation, the real-time temperature and thermal images of the above solutions were recorded. The photothermal conversion efficiency (η) of the samples was determined by monitoring the temperature change of their aqueous solution (100 μg/mL) under NIR laser irradiation at 1.0 W/cm² for 5 min and then turning off the laser. Temperature variations in the materials were closely tracked with an infrared thermal imaging camera. These parameters were calculated according to previously reported equations 41. After incubation at 37 °C for 30 days, the photothermal properties were measured again under the same experimental conditions. To study the pH-responsive release behavior of Zn^2+^ ions, the MTZ nanosheets were dispersed in 10 ml of PBS (pH = 6.5 or 7.4) under gentle shaking. The concentration of Zn^2+^ was detected via an inductively coupled plasma optical emission spectrometer (ICP-OES, Optima 7000 DV, USA).

### Cytocompatibility of MTZ

Macrophages (RAW264.7 cells) and MC3T3-E1 pre-osteoblastic cells were purchased and cultured in DMEM supplemented with 10% FBS and 1% P/S. All cells were maintained in a humidified chamber at 37 °C with 5% CO_2_. To assess the impact of MTZ nanosheets on the viability and proliferation of RAW264.7 and MC3T3-E1 cells, live/dead cell staining and CCK-8 assays were conducted according to the manufacturer's instructions. Briefly, RAW264.7 and MC3T3-E1 cells were separately seeded into 96-well plates at a density of 4×10^3^ cells per well and then cocultured with varying concentrations of MTZ nanosheets. Moreover, after 3 days of coculture, a live/dead cell staining kit (calcein-AM and PI) was used to distinguish live and dead cells.

### Intracellular ROS-scavenging capacity of MTZ

The antioxidant activity of MTZ nanosheets was measured via a DCFH-DA staining assay as previously described 42. Briefly, the cells were seeded in 96-well plates and incubated for 24 h. Subsequently, the medium was replaced with medium containing 1 µg/mL LPS or 500 μM H_2_O_2_ and various concentrations of MTZ. After a further 24 h of incubation, the cells were incubated with DCFH-DA solution at 37 °C for 30 min and then observed under a fluorescence microscope (Olympus, Japan).

### *In vitro* osteogenic differentiation of MTZ

MC3T3-E1 pre-osteoblastic cells were cultured with basal medium for 24 h, followed by the addition of 500 μM H_2_O_2_ with or without MTZ nanosheets in osteogenic induction medium for 7 and 14 days. After 7 days, ALP staining and activity analyses were performed via a BCIP/NBT color development kit and an ALP assay kit, respectively, according to the manufacturer's instructions. After 14 days, the cells were fixed, stained with ARS solution, and observed under an optical microscope. For quantitative analysis of ARS staining, the samples were dissolved in 10% cetylpyridinium chloride, and the absorbance was measured at 562 nm via a microplate reader. Furthermore, osteogenic marker expression in MC3T3-E1 cells was assessed by immunofluorescence. The nanosheets and cells were cocultured under oxidative stress conditions as described above. Following osteoinductive coculture for 7 days, the cells were fixed with 4% paraformaldehyde, permeabilized with 0.1% Triton X-100, blocked with 5% BSA, and incubated with primary antibodies specific for Runx2 and OPN at 4 °C overnight, followed by incubation with appropriate secondary antibodies. After washing with PBS, the samples were counterstained with DAPI. Finally, the stained samples were observed and analyzed via an inverted fluorescence microscope.

### *In vitro* macrophage polarization assessment of MTZ

RAW264.7 cells were cultured in 24-well plates for 24 h, followed by the addition of 1 µg/mL LPS with or without MTZ treatment for 3 days. Afterwards, immunofluorescence staining was performed to detect the expression of iNOS and CD206 (M1 and M2 marker proteins, respectively) in treated macrophages. Briefly, the cells were fixed with 4% paraformaldehyde for 30 min and blocked with 5% goat serum for 1.5 h. Subsequently, the cells were incubated with primary antibodies against iNOS and CD206 at 4 °C overnight. After being rinsed with PBS, the cells were then incubated with the corresponding secondary antibodies for 1 h at 37 °C. Finally, the nuclei were stained with DAPI solution for 15 min, and the stained samples were photographed under an inverted fluorescence microscope. To quantitatively assess the degree of M1/M2-type macrophage polarization, the expression levels of CD86 (M1 marker) and CD206 (M2 marker) were detected via flow cytometry. Briefly, macrophages were subjected to immunofluorescence staining. The cells were harvested via centrifugation and incubated with CD86 (BioLegend) or CD206 (BioLegend) in the dark. After incubation for 20 min, the samples were analyzed via a flow cytometer (ACEA FC500, Beckman Coulter, Fullerton, CA, USA). Next, the levels of inflammation-associated cytokines, including TNF-α, IL-1β, and IL-4, were analyzed via commercial ELISA kits according to the manufacturer's instructions.

### RNA sequencing analysis

RNA sequencing (RNA-seq) was used to assess gene expression in RAW264.7 cells following various treatments. In brief, RAW264.7 cells treated with LPS (1 µg/mL) were cocultured with MTZ nanosheets (100 μg/mL) for two days and collected for RNA-seq analysis. TRIzol reagent was used to extract total RNA from cells according to the manufacturer's instructions. Next, RNA-seq was conducted at Frasergen Bioinformatics Co., Ltd. Differential gene transcripts were analyzed for functional and signaling pathway enrichment via the Kyoto Encyclopedia of Genes and Genomes (KEGG) and Gene Ontology (GO) databases. Differences between the control and MTZ groups were analyzed in predefined gene sets via gene set enrichment analysis (GSEA). In this work, a corrected P value < 0.05 indicated a significant difference in DEGs, as determined by GO and KEGG enrichment analyses.

### Preparation and characterization of hybrid hydrogels

For the fabrication of the SC/MTZ hydrogel, 0.5 g of SFMA and 0.5 g of CMCSMA were dissolved in 10 mL of PBS and stirred vigorously until fully dissolved. Then, heterogeneous MTZ nanosheets (100 mg/mL) and photo-initiator LAP (2 mg/mL) were added to the above solution and thoroughly mixed. Afterward, the mixture was transferred into a mold and incubated at 37 °C for 4 h, followed by exposure to UV light for 30 s to form SC/MTZ. For comparison, SC was prepared via a procedure similar to that above, without the addition of MTZ nanosheets. Vial-tilt experiments were performed to assess hydrogel formation during gelation.

Following gelation, the hydrogels were observed via a superresolution digital microscope (VHX-700, Keyence, Osaka, Japan). FE-SEM and a high-resolution microcomputed tomography system (micro-CT, SkyScan 1276, Bruker, Germany) were used to assess the pore size and microstructure of the hybrid hydrogels. To assess the injectability, adaptability, and moldability of the matrix hydrogels, precursor solutions were injected into various 3D customized molds and irregular bone defects to induce gelation. The compressive properties of the hybrid hydrogels were investigated via a universal mechanical testing system (CMT6503, Shenzhen SANS Test Machine, China). The rheological properties of the hybrid hydrogels were evaluated via a rheometer (Anton Paar MCR92, China). To assess swelling behavior, various freeze-dried hydrogel samples were pre-weighed (M0) and immersed in PBS (pH 7.4) at 37 °C until swelling equilibrium was reached. At predetermined time intervals, the samples were removed from the PBS and weighed (M1) after the surface water was removed using filter paper. To assess degradation behavior, various freeze-dried hydrogel samples were pre-weighed (W0) and then immersed in PBS solution (pH = 7.4) containing 1 U/mL collagenase II at 37 °C. At the scheduled time points, the residual hydrogel samples were removed, freeze-dried, and weighed (W1). Changes in weight and morphology were recorded during the swelling and degradation experiments.

### Evaluation of the photothermal, antioxidant, and stimuli-responsive release properties

To assess the photothermal effect, the hydrogel was subjected to an 808 nm NIR laser at different power densities (0, 0.25, 0.5, and 0.75 W/cm^2^) for 5 min. Next, the hydrogel was implanted in the backs of the rats and then exposed to an 808 nm NIR laser at a power density of 0.5 W/cm^2^ for 5 min. During irradiation, an infrared thermal imager (FLIR Systems, Inc., Wilsonville, OR) was applied to record the temperature change curve and infrared images of the hydrogel surfaces over time. The photothermal stability of the hydrogel was then assessed through five on/off cycles of NIR irradiation. To determine the antioxidant activity, DPPH and ABTS free radical scavenging assays were performed according to a previously reported method 43. In brief, the sample was added to the DPPH or ABTS reaction system and incubated in the dark for 30 min, as described. The SC/MTZ+NIR group was exposed to the 808 nm NIR laser (0.5 W/cm^2^) for 5 min. Finally, the absorbance of each sample was measured via a microplate reader. The release profiles of TA and Zn^2+^ from the composite hydrogels were measured under different conditions. At predetermined time points, the supernatant was collected and replenished with an equal volume of fresh PBS solution. The concentration of TA was calculated using a standard curve, as described previously 44. The Zn^2+^ concentrations of the leaching solutions were measured via ICP-OES using the same method described above.

### *In vitro* antibacterial performance

The antibacterial effects were assessed using *S. aureus* and *E. coli*. The spread plate method, SEM, and live/dead bacteria staining assay were employed. For the group without NIR irradiation, the samples were mixed with a bacterial suspension (1 × 10^6^ CFU/mL) and incubated at 37 °C for 8 h in a relatively humidified atmosphere. For the SC/MTZ+NIR group, the samples were irradiated with an 808 nm NIR laser (0.5 W/cm^2^, 5 min) every 4 h. The group without hydrogel was used as a control group. The treated bacterial suspensions were subsequently serially diluted. A total of 10 μL of each diluted bacterial suspension was plated onto Luria Bertani agar plates and incubated overnight at 37 °C. Finally, the bacterial colonies on the Luria Bertani agar plates were photographed via a digital camera. The absorbance of the bacterial suspensions at 600 nm was measured via a microplate reader. Moreover, the treated *S. aureus* and *E. coli* were fixed with 2.5% glutaraldehyde at 4 °C overnight, followed by gradient dehydration with a series of ethanol solutions. Finally, SEM was used to examine the bacterial morphology and microstructure after different treatments. After the bacterial suspensions were collected in the same way, the bacteria were stained with a LIVE/DEAD Bacterial Viability Kit (Thermo Fisher, USA) for 10 min and then photographed and observed via CLSM.

### Cytocompatibility, cell migration, antioxidant performance, and vascularization

Both BMSCs and HUVECs were seeded at a density of 3 × 10^4^ cells per well in 24-well plates pre-coated with various hydrogels. Additionally, in the SC/MTZ+NIR group, the cells were irradiated with an 808 nm NIR laser (0.5 W/cm^2^) for 5 min daily to meet the mild-temperature PTT requirement (42 ± 1 °C). The same method was used for NIR treatment in all subsequent cell experiments. After coculturing for 1, 2, and 3 days, a CCK-8 assay was utilized to evaluate cell viability and proliferation according to the manufacturer's instructions. For live/dead cell staining, cells were incubated with calcein-AM and PI for 15 minutes after 3 days of coculture and then observed under a fluorescence microscope. For the EdU staining assay, the cells were incubated with EdU for 2 h and then counterstained with Hoechst 33342 according to the manufacturer's protocol. The stained samples were visualized via a fluorescence microscope. For cytoskeleton staining, cells were fixed with 4% paraformaldehyde and permeabilized with 0.2% Triton X-100 for 10 min. The cytoskeleton and nuclei were stained with TRITC-phalloidin and DAPI, respectively. Finally, the cell morphology was observed via fluorescence microscopy. For immunofluorescence staining, the cells were rinsed with PBS and fixed in 4% formaldehyde solution for 15 min as described above. After being washed with PBS, the cells were treated with anti-vinculin for 1 h, followed by incubation with DAPI for 5 min. Images were captured via a fluorescence microscope.

To determine intracellular ROS levels, cells were seeded onto the hydrogel in a 24-well plate as described above and incubated with 500 μM H_2_O_2_-supplemented medium for 12 h. Cells without hydrogel treatment served as the positive control group. After irradiation with an 808 nm NIR laser (5 min, 0.5 W/cm^2^), the amount of ROS produced by both the BMSCs and the HUVECs was detected via a DCFH-DA probe following the manufacturer's instructions. Finally, the samples were observed under a fluorescence microscope and analyzed via ImageJ software (NIH, USA).

To investigate angiogenic potential, extracts of the hydrogels were prepared according to the standard protocol in ISO 10993-12 45. In brief, 100 μL of hydrogel was incubated with 1 mL of cell culture medium at 37 °C for 3 days. The extracts were subsequently collected and filtered through 0.22 μm sterile filter membranes. For the SC/MTZ+NIR group, daily periodic NIR irradiation (808 nm, 0.5 W/cm^2^) was performed for 5 min during incubation. For the *in vitro* wound healing assay, HUVECs were incubated in FBS-free medium for 24 h. Then, a sterilized 200 μL pipette tip was used to create scratches on the cell monolayer. After washing with PBS to remove cell debris, the cells were cocultured with or without the hydrogel extracts for 24 h. The scratch area in the HUVECs was photographed with a microscope and quantitatively evaluated in ImageJ. Additionally, a Transwell assay was performed to assess HUVEC migration following various treatments. After incubation for 24 h, the migrated HUVECs in the Transwell chamber were fixed with 4% paraformaldehyde, stained with 0.1% crystal violet (Solaribo, China) for 15 min, and then observed under a microscope. The angiogenic effects of different hydrogel extracts were also evaluated via a tube formation assay. Briefly, HUVECs were seeded in 6-well plates and treated with extracts of different hydrogels for 24 h. Then, the cells were seeded in 24-well plates precoated with Matrigel (Corning, NY, USA) and cocultured for 8 h. Afterward, the cells were stained with calcein-AM to observe the formation of tubular structures under an inverted fluorescence microscope. Angiogenesis analysis via ImageJ software. The angiogenic potential of various extracts was further assessed using the chorioallantoic membrane (CAM) assay, as previously described 46. In brief, fertilized chicken embryos were initially incubated in an incubator at 37 °C with 60% humidity. After 3 days, the eggshells were gently opened to reveal the CAM structure, and 200 μL of each hydrogel extract was added. The egg opening was sealed with a transparent film, and the eggs were incubated daily to discard any abnormal embryos. After an additional 24 h of incubation, the blood vessels within the CAM were photographed with a digital camera. Additionally, immunofluorescence staining for the angiogenesis-related markers CD31 and HIF-1α was performed to evaluate the angiogenic effects of various hydrogel extracts. The subsequent experimental procedures were identical to those described previously. Additionally, the expression levels of angiogenesis-related genes in HUVECs were evaluated via quantitative real-time polymerase chain reaction (qRT-PCR). Briefly, total RNA was isolated from cellular lysates using TRIzol, and complementary DNA (cDNA) was subsequently synthesized using the HiScript III RT SuperMix Kit according to the manufacturer's instructions. Subsequently, qRT-PCR was performed using primers specific to the genes of interest and ChamQ SYBR qPCR Master Mix on a 7500 Real-Time PCR system (Applied Biosystems, USA). GAPDH was used as the housekeeping gene, and the primer sequences are listed in **Table S1**.

### *In vitro* evaluation of osteogenic activity

To induce an oxidative stress microenvironment, 500 μM H_2_O_2_ was added to the osteogenic induction medium as previously described 14. In brief, BMSCs were exposed to 500 μM H_2_O_2_ for 24 h and then cocultured with the hydrogels. In the SC/MTZ+NIR group, the cells were irradiated with an 808 nm NIR laser (0.5 W/cm^2^) for 5 min daily as described above. After 2 days of incubation, a CCK-8 assay, live/dead staining, and flow cytometry were performed to assess the cytoprotective effects against oxidative stress. Afterward, the mitochondrial membrane potential (MMP) of the BMSCs was evaluated via a JC-1 MMP assay kit in accordance with the provided instructions. Apoptosis was assessed with a deoxynucleotidyl transferase dUTP nick-end labeling (TUNEL) assay kit according to the manufacturer's instructions. Moreover, an immunofluorescence assay was performed to determine caspase-3 expression in BMSCs. Additionally, the expression levels of oxidative stress-related genes in BMSCs were evaluated via qRT-PCR following the procedure described above. The expression levels of the target genes were quantified using the 2-ΔΔCT method and normalized to the reference gene GAPDH. The sequences of primers used are shown in **Table S1**. For ALP, ARS, and Von Kossa staining assays, BMSCs were cocultured with the hydrogels under oxidative stress, following the same procedure described above. After 7 days, ALP staining was performed via a BCIP/NBT color development kit according to the manufacturer's instructions. After 14 and 21 days, the cells were fixed with 4% paraformaldehyde, stained with ARS and Von Kossa solutions according to the manufacturer's instructions, and then observed under an optical microscope. Additionally, immunofluorescence staining and western blotting were used to examine the expression of osteogenesis-related proteins in BMSCs from all experimental groups. The expression levels of osteogenesis-related genes in the BMSCs were also evaluated via qRT-PCR. The subsequent experimental procedures were identical to those described previously. The primer sequences are listed in **Table S1**. Meanwhile, osteogenic proteins were extracted from BMSCs after various treatments via RIPA lysis buffer. Proteins were resolved by SDS polyacrylamide gel electrophoresis (SDS-PAGE) before being transferred to polyvinylidene fluoride (PVDF) membranes. The membranes were blocked with 4% BSA for 2 h and then incubated overnight at 4 °C with specific primary antibodies. The membranes were subsequently washed three times with 0.1% TBST and then incubated with the appropriate secondary antibodies at room temperature for 1 h. Protein signals were visualized via an enhanced chemiluminescence detection system (Tanon, Shanghai, China).

### *In vitro* evaluation of immunomodulatory and osteoblastic inhibitory activity

To induce an oxidative stress microenvironment, RAW264.7 cells were treated with H_2_O_2_ (500 μM) for 24 h as described above and then cocultured with the hydrogels. Moreover, the cells in the SC/MTZ+NIR group were irradiated with an 808 nm NIR laser (0.5 W/cm^2^) for 5 min daily. After coculturing for 24 h, a CCK-8 assay, live/dead staining, and flow cytometry were performed to evaluate the cytoprotective effects against oxidative stress. Afterward, the mitochondrial membrane potential (MMP) of RAW264.7 cells was evaluated via a JC-1 MMP assay kit in accordance with the provided instructions. To assess their anti-inflammatory and immunomodulatory effects, RAW264.7 cells were initially stimulated with LPS (1 μg/mL) for 24 h, followed by coculture with the hydrogels for 3 days. Moreover, the cells in the SC/MTZ+NIR group were irradiated with an 808 nm NIR laser (0.5 W/cm^2^) for 5 min daily as described above. Then, immunofluorescence staining and flow cytometry analysis were conducted as described above. The relative expression of target genes was quantified using the 2-ΔΔCt method, with GAPDH as the internal reference gene. The primer sequences are provided in **Table S1**. Western blot analysis was used to evaluate the expression of inflammation-related proteins in RAW264.7 cells across different treatment groups, as previously described 6.

In accordance with our previously reported methods 47, bone marrow-derived macrophages (BMMs) were obtained from 4-week-old C57BL/6 mice and subsequently cocultured with the hydrogels in α-MEM supplemented with M-CSF (30 ng/mL) for 48 h. To evaluate osteoblastic differentiation, BMMs were seeded in a 48-well plate and cultured in osteoclast medium supplemented with 75 ng/mL NF-κB receptor activator (RANKL) for 7 days. After various hydrogel treatments with or without NIR irradiation (808 nm, 0.5 W/cm^2^) for 5 min daily, the cells were subjected to TRAP staining using a TRAP activity staining kit at 37 °C for 60 min and then observed under an optical microscope. For the F-actin ring formation assay, the cells were fixed with 4% paraformaldehyde for 15 min, permeabilized with 0.1% Triton X-100 for 10 min, and blocked with 5% BSA for 2 h. Next, the osteoclast cytoskeleton was stained with phalloidin and DAPI. Finally, the stained samples were observed and analyzed via an inverted fluorescence microscope. The expression of osteoclastogenesis-related markers was then assessed through immunofluorescence staining, qRT-PCR, and western blot analysis. The primer sequences for the relevant genes are listed in **Table S1**.

### *In vitro* immunomodulation-mediated osteogenic differentiation

The immunomodulation-mediated osteogenic effects were evaluated using RAW264.7 cells as previously reported 16. First, conditioned medium was collected by culturing macrophages under various hydrogel stimuli with or without NIR irradiation (808 nm, 0.5 W/cm^2^). Immunofluorescence staining assay was used to evaluate the expression of beneficial chemokines in the supernatants of treated macrophages across different experimental groups. Additionally, the expression levels of osteogenic cytokines were quantified by ELISA according to the manufacturer's protocols. Furthermore, the supernatant concentrations of TA and Zn were assayed via UV-vis spectrophotometry and ICP-OES, respectively. To evaluate cell migration, both MC3T3-E1 pre-osteoblastic cells and BMSCs were treated with conditioned medium and subjected to Transwell migration and wound-healing assays using the same methods described above. Furthermore, after culturing for 7 and 14 days, ALP activity, ARS staining, Von Kossa staining, and immunofluorescence staining of Runx2 and OPN were performed. The expression of osteogenic markers, including Col-1, Runx2, OPN, and OCN, in BMSCs was assessed by qRT-PCR and western blot analysis after 7 days of culture. The experimental process details are identical to those described previously.

### *In vivo* subcutaneous implantation

Eighteen BALB/c mice (male, 6-8 weeks, 20-25 g) were utilized in this study and subjected to subcutaneous implantation, as previously described 48. All animal procedures were approved by the Animal Care and Use Committee of Wuhan University, and the protocols complied with the Guide for the Care and Use of Laboratory Animals. After administering anesthesia and disinfecting the surgical site, various hydrogels were implanted into subcutaneous pockets on the backs of the mice. The SC/MTZ+NIR group was exposed to the NIR laser as described previously. After 2 weeks of implantation, the mice were euthanized, and the hydrogels with surrounding tissue were collected, fixed in 4% polyformaldehyde, dehydrated through a series of alcohol solutions, and embedded in paraffin wax for sectioning. The samples were subsequently subjected to hematoxylin and eosin (H&E) staining and Masson's trichrome (MST) staining to assess tissue ingrowth and collagenous fibrotic capsule formation. The local levels of ROS in the hydrogel and surrounding tissue were then evaluated using a dihydroethidium (DHE) fluorescence probe on frozen slices. To investigate macrophage polarization, the local inflammatory response, and neovascularization *in vivo*, immunohistochemistry (iNOS, CD206, and CD31) and immunofluorescence (TNF-α, IL-10, and CD31) assays were conducted according to the manufacturers' instructions. In addition, to measure the concentration of inflammation-related mediators, the samples, along with the adjacent subcutaneous tissues, were collected and frozen in liquid nitrogen for ELISA experiments according to the manufacturer's instructions. Finally, to assess the biosafety of the implanted materials *in vivo*, the major organs, including the heart, liver, spleen, lung, and kidney, were collected for H&E staining at 4 weeks post-implantation. In particular, blood samples were used to evaluate serum biochemical indicators.

### *In vivo* critical-sized cranial defect model in diabetic rats

Sixty male Sprague-Dawley (SD) rats (8 weeks old, weighing 250 ± 25 g) were intraperitoneally injected with STZ (55 mg/kg), freshly dissolved in 0.1 M citrate buffer (pH 4.5), to induce a diabetic model according to previously formulated protocols 49. Blood glucose levels were monitored from the tail vein using a Roche glucose meter and then weekly. Diabetic rats were induced for 2 weeks, during which fasting blood glucose levels consistently exceeded 16.7 mmol/L. The diabetic rats described above were randomly and equally assigned to groups for *in vivo* experiments. After anesthesia with 2.5% sodium pentobarbital (40 mg/kg), two critical-sized cranial defects (5 mm in diameter) were created using a dental trephine drill, which was flushed and cooled with physiological saline while maintaining dural integrity throughout the surgical procedure. In the hydrogel-treated groups, 100 µL of hydrogel precursor solution was injected into the defect region of each rat, followed by UV irradiation to *in situ* photo-crosslink the hydrogels. In the control group, 100 µL of sterile saline was injected into the defect sites with no implanted materials. The animals in the SC/MTZ+NIR group were irradiated with an 808 nm NIR laser (0.5 W/cm^2^, 5 min) every other day for 8 weeks. The laser probe was fixed at a perpendicular distance of 5 cm from the exposed cranial defect site using a custom stand, ensuring a uniform, stable spot size that covered the entire defect area. At 2, 4, and 8 weeks after implantation, the SD rats were euthanized, and the cranial tissues were harvested and fixed in 4% paraformaldehyde for 2 days.

For radiological analysis, the samples were subjected to micro-CT (SkyScan1276, Bruker, Germany) with specific settings: 6.5 µm, 58 kV, 215 µA, and 0.25 mm aluminum. 3D reconstruction and analysis were subsequently conducted, and bone morphometric parameters, including bone tissue volume/total tissue volume (BV/TV), bone mineral density (BMD), trabecular thickness (Tb.Th), trabecular number (Tb.N), and trabecular separation (Tb.Sp), were measured and analyzed. After micro-CT analysis, the fixed samples were decalcified in 10% ethylenediaminetetraacetic acid (EDTA) solution, dehydrated, embedded in paraffin, and cut into sections (5 μm thick). At 2 weeks after surgery, local ROS levels in the bone defect area were detected by DHE staining of frozen sections of collected bone tissue, as previously described 50. Immunohistochemical staining of HO-1, TNF-α, and IL-10 was performed to analyze oxidative stress and the inflammatory response. Immunofluorescence staining was performed to assess the expression of iNOS (M1 marker) and CD206 (M2 marker). At 4 and 8 weeks after surgery, the above sections were subjected to H&E, MST, and Goldner's trichrome (GST) staining according to the manufacturer's instructions. An optical microscope was then used to observe the slices. In addition, immunohistochemical staining of BMP-2 and VEGF was performed to analyze early osteogenic and angiogenic potential. Immunofluorescence staining of CD31, α-SMA, CD44, and CD90 was performed to evaluate blood vessel formation and stem cell recruitment. Additionally, some harvested samples from the bone defect site were immediately frozen in liquid nitrogen and stored at -80 °C for qRT-PCR. Furthermore, immunohistochemistry and immunofluorescence were carried out to evaluate the expression of osteogenic marker proteins, including Col-1, Runx2, OPN, and OCN. Osteoclastic activity was also assessed by TRAP immunofluorescence staining.

### Statistical analysis

In the present study, all the data are presented as the mean ± standard deviation (mean ± SD). Statistical analysis was performed using Origin 2018 software (Origin Lab Corporation, USA) by one-way ANOVA with Tukey's test. Data with abnormal distribution or heterogeneity of variance were analyzed by the Mann-Whitney U test and Kruskal-Wallis's nonparametric test. All experiments were conducted with at least three independent replicates unless otherwise specified. In all figures, values were considered significant at p* or p^#^, with a p value < 0.05, and highly significant at p** or p^# #^, with a p value < 0.01.

## Results and Discussion

### Preparation and characterization of MTZ nanosheets

**Figure 1:**
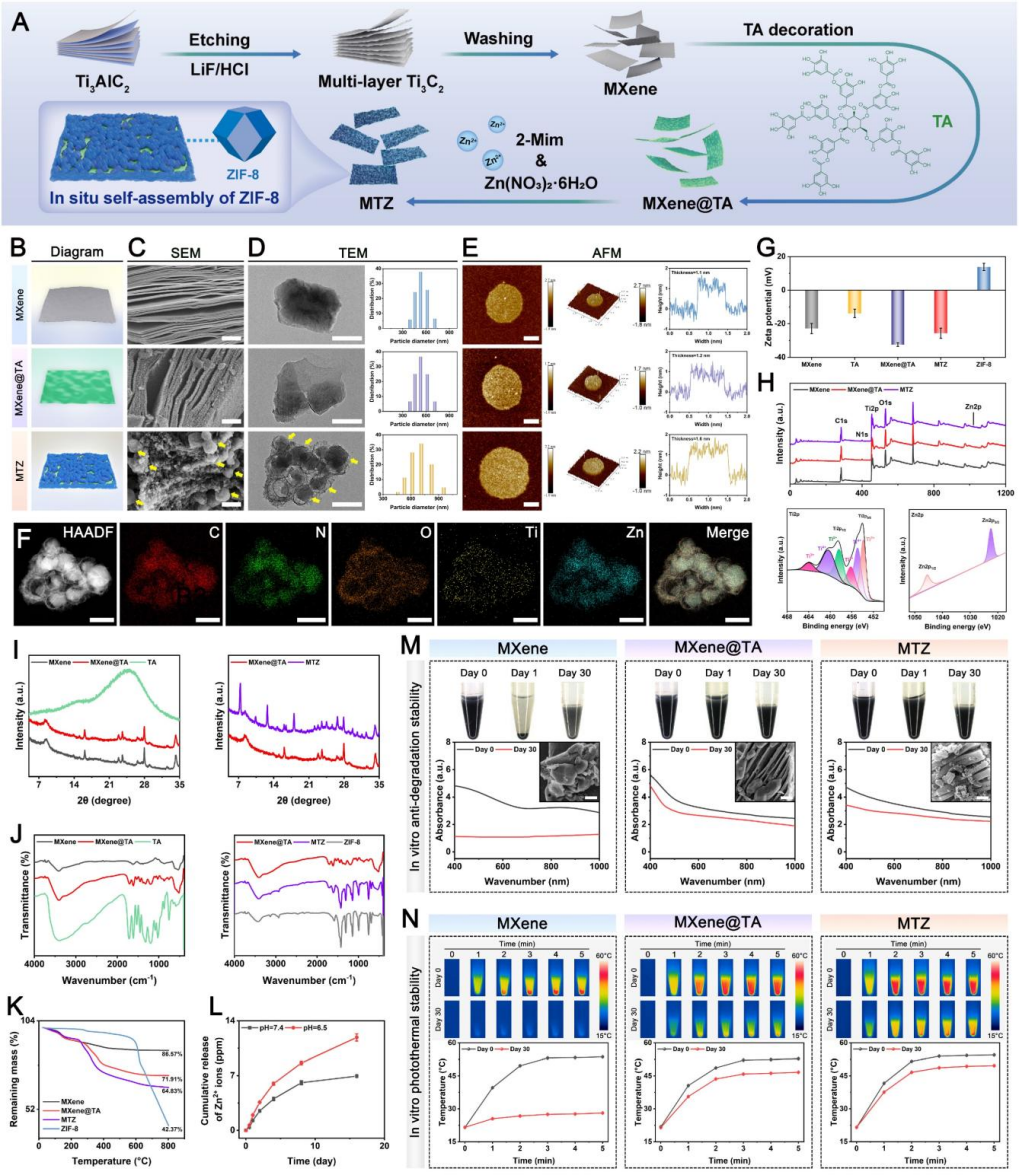
** Structural characterization of MTZ. (A)** Diagram depicting the MTZ synthesis procedure. **(B)** Graphical demonstration of the as-designed nanomaterials.** (C-E)** SEM, TEM, and AFM images, along with size distributions and height profiles, of various samples. The yellow arrow represents the decorated ZIF-8 nanoparticle. Scale bar: 200 nm.** (F)** EDS elemental mapping images of MTZ. Scale bar: 200 nm.** (G)** Zeta potential of the different samples. **(H)** XPS survey of various samples and high-resolution XPS spectra for Ti2p and Zn2p signals in MTZ. **(I-K)** XRD patterns, FTIR spectra, and TGA curves of the different samples. **(L)** Cumulative Zn^2+^ ion release from MTZ at pH values of 6.5 and 7.4. **(M-N)** The *in vitro* physiological and photothermal stability of MXene, MXene@TA, and MTZ was assessed by measuring their temperature profiles and real-time infrared thermal images under 808 nm irradiation at 1.5 W/cm². Scale bar: 200 nm. Data are expressed as mean ± SD (n = 3).

In this work, a multifunctional therapeutic nanoplatform (MTZ) with excellent ROS-scavenging, immunomodulatory, cytoprotective, and osteogenic activities was initially prepared via a polyphenol-inspired *in situ* self-assembly strategy **(Figure 1A)**. Generally, combining MXene and ZIF-8 is difficult because the MXene surface lacks sufficient active sites for ZIF-8 to undergo *in situ* assembly. To address these challenges, MXene was functionalized with TA, which incorporated numerous catechol groups on its surface, forming MXene@TA. The polyphenolic structure of TA provides MXene with various interactions, including hydrogen bonding, π-π stacking, electrostatic and hydrophobic interactions, and oxidation-related covalent bonds. Consequently, catechol-rich TA provided essential surface-active sites for the *in situ* self-assembly of the ZIF-8 nanoparticles. During self-assembly, MXene@TA, which is abundant in negatively charged oxygen-containing groups such as hydroxyl and carboxyl groups, provides multiple nucleation sites for 2-methylimidazole (2-Mim) adsorption via electrostatic interactions. Subsequently, MTZ heterojunctions were prepared via coordination interactions between Zn^2+^ and 2-Mim, resulting in ZIF-8 formation *in situ* on the surface of MXene@TA. Furthermore, TA functionalization potentially enhanced the biological activity and structural stability of the MXene nanosheets, allowing them to disperse homogeneously in the hydrogel matrix for subsequent gelation.

As shown in **Figure 1B-D**, the MXene nanosheets possess accordion-like multilayer nanostructures. The 2D ultrathin sheet-like structure, featuring a large specific surface area, offers abundant nucleation sites for ZIF-8 nanoparticles that display a spherical granular morphology **(Figure S1)**, consistent with a prior report 51. Notably, unlike the smooth surface of pristine MXene nanosheets, the MXene@TA nanosheets exhibited a rough surface with numerous nanodots, suggesting strong interactions between TA and MXene. In the TA modification process, multivalent titanium ions interact with catechol groups in TA to create a metal-phenolic network (TA-Ti complexes) that uniformly coats the surface of the synthesized nanosheets. Following the *in situ* growth of the ZIF-8 nanoparticles, numerous spherical nanoparticles were tightly bound to the 2D MXene@TA layer, forming unique heterojunction structures of MXene, TA, and ZIF-8, as confirmed by SEM images **(Figure S2A)**. The atomic force microscopy (AFM) images **(Figure 1E)** clearly reveal that the MTZ heterojunctions (~1.6 nm) are thicker than the MXene heterojunctions (~1.1 nm) and MXene@TA heterojunctions (~1.2 nm), suggesting the successful anchoring of the ZIF-8 nanoparticles on the nanosheet surface. Energy-dispersive X-ray spectroscopy (EDS) analysis **(Figure 1F)** was conducted further to examine the heterojunction structure of the MTZ nanosheet. The EDS elemental mapping images demonstrated a consistent distribution of C, N, O, Ti, and Zn across the sample, corroborating the morphological observations of the nanosheets. These results suggest that the abundant phenolic hydroxyl groups in TA enhance the stability and adhesion of MXene by serving as an intermediate layer, leading to strong affinity and interfacial interactions between the two materials. Dynamic light scattering (DLS) data **(Figure 1D)** indicate that MXene and MXene@TA have comparable particle sizes. In contrast, MTZ is slightly larger in size, confirming the incorporation of ZIF-8 into the MXene substrate via a TA-mediated *in situ* self-assembly approach. The surface modification of the negative polyphenol reduced the MXene zeta potential from -22.8 to -32.5 mV, which aligns with previous studies 34. Following the combination of 2-Mim and Zn^2+^, the potential increased to -25 mV **(Figure 1G)**, indicating that ZIF-8 self-assembled into the TA-functionalized MXene in situ. The results collectively offer initial evidence for the successful synthesis of MTZ heterojunctions, considering both morphological and structural aspects.

**Figure 1I** shows the broad peak at 2θ = 24.5° of the TA sample, indicating its amorphous nature. Compared with that of pristine MXene, the diffraction peak at 2θ = 8° for the (002) lattice plane of MXene@TA is slightly to the left, indicating increased interlayer spacing, likely due to TA molecule intercalation. For the XRD pattern of MTZ, both the (002) diffraction peak of MXene and the (110), (200), and (211) diffraction peaks belonging to ZIF-8 can be found, indicating that they occur in the MTZ heterojunction. The size and structure of the nanosheets remained unchanged after TA grafting. **Figure 1J** illustrates that both ZIF-8 and MTZ exhibit a characteristic peak at 420 cm^-1^, indicative of Zn-N bond vibration, confirming the successful integration of ZIF-8 with the nanosheets. The vibration peak at 520 cm^-1^, associated with Ti-O vibrations, confirms the formation of MXene nanosheets. The 520 cm^-1^ vibration peak corresponds to the Ti-O vibration, confirming the formation of MXene nanosheets. The MXene@TA spectrum exhibited a C=O stretching peak at 1710 cm^-1^ and benzene ring skeleton peaks at 1450-1616, 872, and 760 cm^-1^, confirming successful TA molecule decoration. The -OH vibrational peak shifts from 3423 cm^-1^ in MXene to 3408 cm^-1^ in MXene@TA, suggesting hydrogen bond formation. After TA modification, the catechol groups on the MXene surface serve as anchor sites for *in situ* ZIF-8 self-assembly. **Figure S2B** illustrates that the MTZ heterojunction forms due to electrostatic interactions between negatively charged MXene@TA and positively charged 2-Mim, followed by coordination between Zn^2+^ and 2-Mim, leading to the *in situ* growth of ZIF-8 nanoparticles on 2D MXene@TA nanosheets. These findings prove the successful construction of MTZ nanosheets with heterogeneous structures. The full-scan XPS survey spectra corroborated the EDS elemental mapping results by confirming the presence of C 1s, N 1s, O 1s, Ti 2p, and Zn 2p peaks in the MTZ heterojunction. **Figure 1H** shows a stronger C 1s peak for MXene@TA than for MXene, confirming successful TA loading. Discernible peaks at 1022.5 and 1045.5 eV correspond to Zn 2p3/2 and Zn 2p1/2, respectively, validating the incorporation of ZIF-8 nanoparticles onto MXene@TA. MTZ exhibited four unique peaks at 281.8, 284.8, 286.1, and 288.8 eV, corresponding to C-Ti, C-C, C-O, and O-C=C bonds, respectively, distinguishing it from pure MXene. The MTZ spectrum displayed high-resolution N 1s peaks at 400.1 and 402 eV, attributed to the C-N and C=N bonds, respectively **(Figure S3)**. As shown in **Figure 1K** and **Figure S4**, the TG and DTG analyses indicated that at 800 °C, the residual weights for MXene, MXene@TA, and MTZ were 86.57%, 71.91%, and 64.83%, respectively, demonstrating the effective incorporation of TA and ZIF-8 into the MXene nanosheets. The loading amounts of TA molecules and ZIF-8 on MXene are approximately 14.66% and 7.08%, respectively. To mimic the bone injury microenvironment, samples were tested under weakly acidic (pH 6.5) and neutral (pH 7.4) conditions **(Figure 1L)**. The study showed that Zn^2+^ ions were released slowly and sustainably over 28 days at physiological pH (7.4), with a significantly increased release rate under mildly acidic conditions (pH 6.5). This highlights the responsiveness of MTZ nanosheets to the acidic environment of diabetic bone defect sites, which have a low pH of approximately 5.5-6.5 52. The pH-responsive release characteristics of ZIF-8-based nanomaterials align with those reported in prior studies, facilitating controlled Zn^2+^ ion release in the acidic microenvironment of bone injuries.

To undertake further *in vivo* applications, the structural and photothermal stability of the MTZ heterojunction in PBS was assessed. **Figure 1M** illustrates that both MXene@TA and MTZ remained well dispersed in PBS without precipitation for 30 days, unlike MXene, which precipitated within a few days. Moreover, the black color of the MXene dispersion gradually fades over time because of rapid oxidation. Compared with the MXene dispersion, the TA and ZIF-8 coatings effectively minimized color changes in the MXene@TA and MTZ dispersions, indicating their stable dispersion in water. Studies indicate that MXene is prone to reacting with water and oxygen, leading to reduced chemical stability 53. In this work, the assembled heterogeneous structure (TA and ZIF-8) allows for stable dispersion in an aqueous solution, preventing aggregation and improving the stability of MXene. UV-vis spectroscopy and SEM analysis verified that TA and ZIF-8 effectively shielded the MXene from oxidation **(Figure 1M)**. This protection likely results from hydrogen bonding occupying the reactive sites of MXene and enhancing hydrophobic MOF structures, thereby blocking water and oxygen molecules and preserving structural integrity and properties. On day 30, the absorbance intensity of the MXene solution dramatically decreased, whereas the absorbance intensity of both the MXene@TA and MTZ solutions only slightly decreased, indicating the protective role of TA and ZIF-8, which greatly enhanced the stability of the MXene nanosheets against oxygen attack. These findings demonstrated that the incorporation of TA and ZIF-8 endows the MTZ heterojunction with superior anti-degradation and dispersion properties, which is attributed primarily to the abundant buffering groups (particularly phenolic hydroxyl groups) present on the nanosheets. This enhanced colloidal stability in physiological environments is essential for long-term therapeutic applications, which establishes a reliable foundation for subsequent hydrogel encapsulation and biological effect modulation.

We examined the photothermal conversion efficiency of a nanosheet suspension exposed to 808 nm NIR laser irradiation at 1.5 W/cm². **Figure 1N** demonstrates that after 5 min of NIR irradiation on day 0, all the samples consistently reached a temperature of approximately 52 °C, indicating that the photothermal properties of the MXene remained stable following the incorporation of TA and ZIF-8. The MTZ heterojunction demonstrated a notable photothermal conversion efficiency (η) of 32.8%. This efficiency is relatively high compared with that of several previously reported photothermal agents, including Au nanorods (21%), Cu_9_S_5_ nanocrystals (25.7%), and Cu_2-x_Se nanocrystals (22%) 54, confirming its outstanding photothermal effect. After 30 days of incubation, MXene@TA and MTZ exhibited significantly higher maximum temperatures than MXene, demonstrating enhanced photothermal stability due to the incorporation of TA and ZIF-8. These findings confirm the successful synthesis and TA coating of the MXene nanosheets, which enabled the *in situ* growth of the ZIF-8 nanoparticles, leading to the formation of the MTZ heterojunction. The results demonstrate that MTZ heterojunctions with improved structural stability, dispersion, anti-degradation, and photothermal properties were successfully developed, making them promising candidates for sustained mild photothermal therapy and drug delivery systems in biomedical applications.

### Assessment of the biological activities of MTZ nanosheets

2D Ti_3_C_2_T_x_ MXene nanosheets have shown considerable promise in tissue engineering and regenerative medicine, particularly for bone defect healing and wound repair, owing to their outstanding biocompatibility and functionalities 55. Due to the potential toxicity of MXene nanosheets, high doses may lead to adverse effects.

**Figure 2:**
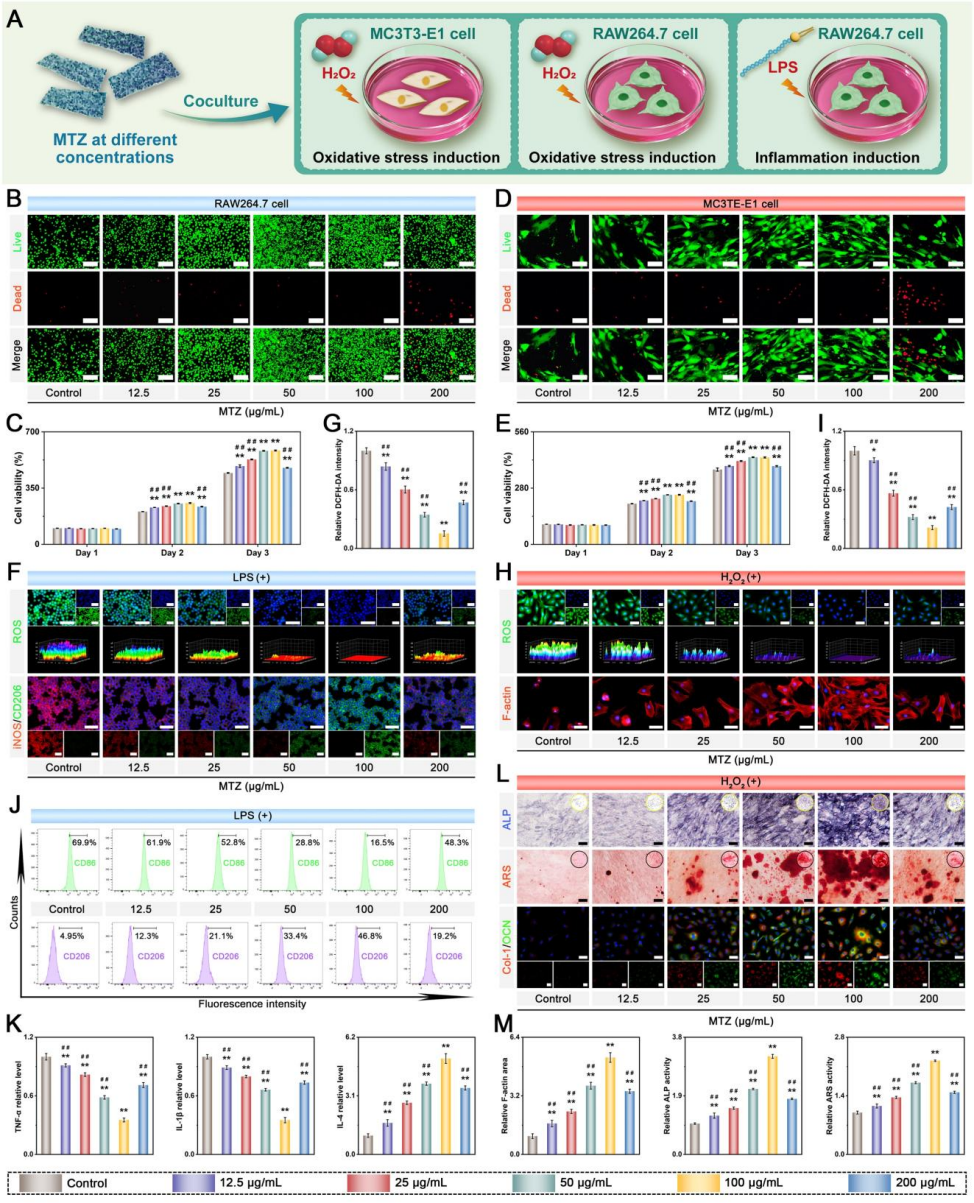
** Biological characterization of MTZ. (A)** Schematic illustration of the experimental design. **(B-C)** Assessment of macrophage viability and live/dead staining following exposure to varying nanosheet concentrations. Scale bar: 100 μm. **(D-E)** Assessment of osteoblast viability and live/dead staining following treatment with varying nanosheet concentrations. Scale bar: 100 μm. **(F-G)** Immunofluorescence images of ROS, CD86, and CD206 in macrophages after different treatments and the corresponding fluorescence intensity of DCFH-DA. Scale bar: 50 μm. **(H-I)** Immunofluorescence images of ROS and F-actin in osteoblasts after different treatments and the corresponding fluorescence intensity of DCFH-DA. Scale bar: 50 μm.** (J)** Flow cytometry was used to analyze CD86 and CD206 expression in macrophages after various treatments. **(K)** ELISA was used to analyze the secretion of proinflammatory cytokines (TNF-α and IL-1β) and the anti-inflammatory cytokine (IL-4) by macrophages following various treatments. **(L)** Images of ALP, ARS, and immunofluorescence staining for Col-1 and OCN in osteoblasts following various treatments. Scale bar: 50 μm. **(M)** Quantitative analysis of F-actin staining, ALP staining, and ARS staining. Data are presented as the mean ± SD (n = 3). *P < 0.05 and **P < 0.01 indicate significant differences compared with the control group. ^#^P < 0.05 and ^# #^P < 0.01 indicate significant differences compared with the 100 μg/mL-treated group.

To identify the optimal concentration of functionalized MXene nanosheets for further study, we first performed *in vitro* cytotoxicity assessments, as nanomaterial compatibility is crucial for tissue regeneration applications. In this work, MTZ nanosheets at different concentrations were cocultured with both RAW264.7 macrophages and MC3T3-E1 pre-osteoblasts for several days **(Figure 2A)**. **Figure 2B-E** reveals enhanced cell growth and survival with increasing nanosheet concentrations, suggesting minimal toxicity within the 0-100 µg/mL range. The increase in cell proliferation is primarily due to the phenolic hydroxyl groups in TA and the bioactive Zn^2+^ in ZIF-8, which significantly promote cell growth and survival, resulting in a notable increase in RAW264.7 cells and BMSCs. The high specific surface area and heterogeneous structure of MTZ provide abundant active sites, enhancing cell-material interactions and facilitating the adsorption and release of bioactive molecules, thereby boosting its cellular activity. MTZ at 200 µg/mL exhibited significant cytotoxicity, reducing cell viability, although tolerance levels varied among different cells **(Figure 2C and Figure 2E)**. No significant difference in cell viability was observed between the 50 µg/mL and 100 µg/mL MTZ treatment groups. After 3 days of coculture, cell viability significantly increased in the 50 μg/mL and 100 μg/mL MTZ groups compared with the other groups. Increasing the MTZ concentration to 200 μg/mL significantly reduced the viability and proliferation of both cell lines, as demonstrated by live/dead staining and CCK-8 assays. Nanosized materials are readily engulfed by cells and accumulate, potentially posing a cytotoxic risk 56. The cytotoxicity observed in this study may be attributed to oxidative stress induced by elevated TA and Zn^2+^ levels, as well as cytomembrane damage caused by lamellar Ti_3_C_2_T_x_ MXene nanosheets.

Under diabetic conditions, increased ROS production and the resulting chronic inflammation at sites of bone injury impede tissue repair and regeneration. This dysfunction, driven by exacerbated oxidative stress, disrupts cellular metabolism and causes irreversible tissue damage 14. To effectively alleviate oxidative stress and inflammation in the pathological microenvironment, we designed natural polyphenol-functionalized MXene nanosheets with abundant phenolic hydroxyl groups and intrinsic enzyme-like activities. The antioxidant effects of MTZ on both RAW264.7 cells and BMSCs in response to H_2_O_2_ or lipopolysaccharide (LPS) stimulation were subsequently investigated. The cells were exposed to oxidative stress via H_2_O_2_ (500 µM) or LPS (1 μg/mL) before nanosheet treatment. The ability of the nanosheets to scavenge intracellular ROS was assessed via the use of 2′,7′-dichlorofluorescin diacetate (DCFH-DA) as a fluorescent probe. **Figure 2F-I** demonstrates that H_2_O_2_ and LPS stimulation elevated intracellular ROS levels, whereas MTZ nanosheets effectively reduced oxidative stress and eliminated intracellular ROS, confirming their role in maintaining the microenvironmental redox balance. The antioxidant activity of the reactive groups and active sites in the MTZ nanosheets accounts for this trend. Both polyphenols (e.g., TA) and MXene nanosheets can react with free radicals, forming stable intermediates and achieving free radical scavenging, further supporting the ROS-scavenging performance of MTZ. Notably, overproduction and accumulation of ROS can induce cytoskeletal crumpling and deformation. As depicted in **Figure 2H**, MC3T3-E1 cells with high ROS levels exhibited poorly organized skeletons and a shrunken morphology. MTZ nanosheet treatment significantly decreased intracellular ROS levels and normalized the cytoskeletal structure. Compared with other nanosheet concentrations, 100 µg/mL MTZ nanosheets resulted in enhanced ROS scavenging and cytoskeleton restoration, as evidenced by notable elongation and F-actin filament extension **(Figure 2M)**. High concentrations of MTZ nanosheets, particularly at 200 μg/mL **(Figure 2C and Figure 2E)**, decreased cell viability and elevated oxidative stress. This effect is likely due to mitochondrial dysfunction and ROS production promoted by high TA concentrations in cell cultures. These findings imply that MTZ heterojunctions can efficiently remove excess ROS and prevent oxidative stress-related cellular damage, highlighting their potential for treating diabetic bone defects.

Substantial evidence indicates that persistent chronic inflammation and high oxidative stress induce macrophages to retain the M1 proinflammatory phenotype, which is considered detrimental to long-term bone repair. M1 macrophages release inflammatory factors such as IL-6, IL-1β, and TNF-α, creating a proinflammatory microenvironment that harms cells at bone injury sites and hinders bone defect repair 57. The uptake of nanosheets is anticipated to lower ROS levels in M1 macrophages, facilitating their transition from a proinflammatory M1 phenotype to an anti-inflammatory M2 phenotype, thereby aiding in bone defect treatment. Consequently, additional studies were performed to examine the anti-inflammatory and immunomodulatory effects of MTZ. RAW264.7 cells were exposed to 1 μg/mL LPS for 24 h to simulate an inflammatory microenvironment, followed by a 2-day coculture with either PBS or nanosheets. The expression of macrophage phenotype markers was investigated via immunofluorescence staining. **Figure 2F** illustrates that LPS treatment promoted macrophage polarization toward the proinflammatory M1 phenotype, as indicated by increased iNOS expression and reduced CD206 expression. MTZ treatment notably suppressed iNOS expression and significantly increased CD206 expression, with the most substantial effect at 100 μg/mL. This is due to the combined anti-inflammatory and immunomodulatory effects of TA and ZIF-8, which significantly enhance the local inflammatory environment and facilitate tissue regeneration. Research has indicated that TA and ZIF-8 exhibit potent antioxidant and anti-inflammatory effects, effectively reducing oxidative stress and suppressing abnormal inflammatory responses 23. Moreover, the strong ROS-scavenging capacity of MTZ may facilitate the transition from the M1 to the M2 phenotype, potentially reducing the duration of diabetes mellitus-induced inflammation. Flow cytometry analysis corroborated the immunofluorescence findings, which revealed that MTZ treatment decreased the percentage of LPS-induced M1 macrophages (CD86^+^) and promoted M2 macrophage polarization** (Figure 2J)**. Meanwhile, the levels of inflammatory cytokines were measured by ELISA **(Figure 2K)**. MTZ dose-dependently suppressed the expression of the proinflammatory cytokines TNF-α and IL-1β while enhancing IL-4 expression. This study demonstrated that MTZ nanosheets successfully induce macrophage reprogramming to the anti-inflammatory M2 phenotype *in vitro*, thereby disrupting the inflammatory response and ROS feedback loop and boosting anti-inflammatory factor expression. The immunomodulatory properties of MTZ nanosheets are beneficial for restoring the immune microenvironment and facilitating early-stage diabetic bone healing.

Given the beneficial effects of MTZ heterojunctions on antioxidant, anti-inflammatory, and immunomodulatory functions, we investigated whether MTZ could enhance the osteogenic differentiation of MC3T3-E1 cells under oxidative stress. MC3T3-E1 cells were cultured in osteogenic medium supplemented with 500 μM H_2_O_2_, with or without MTZ, for 7 and 14 days to mimic the oxidative stress conditions associated with diabetes mellitus. The osteogenic potential of MTZ was evaluated through an alkaline phosphatase (ALP) activity assay, alizarin red S (ARS) staining, and immunofluorescence staining **(Figure 2L)**. After 7 days, the ALP staining assay showed that MTZ increased ALP expression in MC3T3-E1 cells, with the most significant effect at 100 μg/mL. Quantitative analysis confirmed that ALP expression declined with increasing concentration. Consistent results were also obtained via ARS staining, with MTZ at 100 μg/mL significantly increasing calcium deposition in MC3T3-E1 cells. The findings indicated that ECM mineralization was considerably greater in the 50 μg/mL and 100 μg/mL MTZ-treated groups than in the control and other MTZ-treated groups, with the 100 μg/mL group showing the highest levels **(Figure 2M)**. This study suggested that MTZ nanosheets can enhance the osteogenic potential of MC3T3-E1 cells by reducing ROS levels, facilitating cell recovery, and leveraging the osteogenic properties of Zn^2+^ ions. Similarly, an immunofluorescence staining assay was further employed to corroborate the findings of ALP activity and ARS staining. **Figure 2L** shows that the control group exhibited minimal fluorescence. In contrast, the MTZ group showed a concentration-dependent increase in osteogenic protein expression (Col-1 and OCN), with the most pronounced increase at 100 μg/mL. The MTZ concentration of 100 μg/mL was selected for subsequent experiments, as it exhibited low cytotoxicity and satisfactory biological activity. These findings indicate that MTZ significantly enhances both early osteogenic differentiation and late calcium nodule formation in MC3T3-E1 cells while increasing the expression of osteogenic markers under oxidative stress.

Osteoinductive capacity and immunomodulatory functionality represent critical determinants of the therapeutic efficacy of bone repair biomaterials 58. In this study, MXene functions as a nanozyme with peroxidase (POD)- and catalase (CAT)-like activities, effectively catalyzing the breakdown of high-concentration H₂O₂ into H₂O and O₂ via surface redox cycles involving Ti³⁺/Ti⁴⁺ transformations, thereby providing a primary catalytic defense barrier 54. TA acts as a broad-spectrum radical scavenger and forms stable Ti-coordination bonds with MXene 59, creating an antioxidant-enriched nanointerface that guarantees the immediate neutralization of secondary radicals during catalysis. Within the acidic bone microenvironment of diabetes mellitus, TA is released in a pH-responsive manner and internalized by macrophages, where it directly suppresses NF-κB signaling to facilitate M2 macrophage polarization 25. ZIF-8 primarily functions as a protective carrier and sustained-release platform, encapsulating MXene@TA to prevent aggregation and ensure localized retention. Its gradual degradation under acidic conditions enables the controlled release of both the MXene@TA complex and Zn²⁺ ions. The released Zn²⁺ exerts a synergistic effect with TA to potentiate M2 macrophage polarization via complementary signaling cascades, thereby further enhancing immunomodulatory activity and constructing a dual-signal regulatory machinery that enables sustained pro-regenerative immune remodeling.

**Figure 3:**
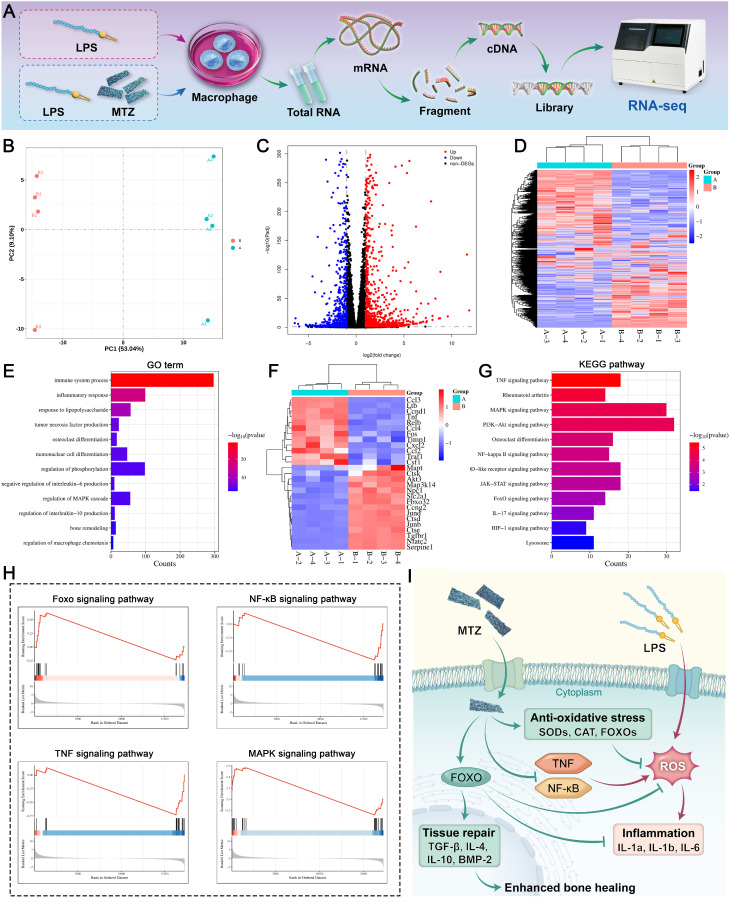
** Transcriptome sequencing analysis of macrophages in an inflammatory microenvironment, with and without MTZ coculture. (A)** Schematic diagram of the establishment of the LPS-induced inflammatory model and the subsequent experimental design. **(B)** PCA analysis. **(C)** Volcano plot of DEGs in macrophages. **(D)** Heatmap of DEGs between the control and MTZ groups. **(E)** GO enrichment analysis of DEGs.** (F)** Heatmap analysis of DEGs across various pathways. **(G)** KEGG enrichment analysis of DEGs. **(H)** GSEA analysis of significant DEGs in the control and MTZ groups. **(I)** Schematic diagram illustrating the potential mechanism by which MTZ modulates the immune microenvironment and promotes bone defect repair. A: Control group. B: MTZ group.

To clarify how MTZ regulates immune responses during inflammation, RNA-seq was performed on RAW264.7 macrophages. The LPS-treated group alone was selected as the control group, which is beneficial for elucidating the biological effects of MTZ **(Figure 3A)**. Principal component analysis (PCA) revealed significant differences between the LPS and MTZ groups **(Figure 3B)**. The volcano plots and heatmaps revealed extensive differentially expressed genes (DEGs) induced by MTZ, with 1002 upregulated genes and 618 downregulated genes **(Figure 3C-D)**. Gene Ontology (GO) enrichment analysis indicated that the differentially expressed genes (DEGs) were mainly associated with immune system processes, the inflammatory response, osteoclast differentiation, and bone remodeling, highlighting a significant correlation between these aspects and the effect of MTZ **(Figure 3E-F)**. Kyoto Encyclopedia of Genes and Genomes (KEGG) enrichment analysis indicated that the DEGs linked to inflammation were concentrated in the TNF, MAPK, PI3K/AKT, IL-17, NOD-like receptor, FOXO, JAK/STAT, and NF-κB signaling pathways **(Figure 3G)**. Furthermore, gene set enrichment analysis (GSEA) showed that MTZ promoted activation of the FOXO signaling pathway, which is closely associated with antioxidant activity, while inhibiting the TNF, MAPK, and NF-κB pathways involved in inflammatory responses **(Figure 3H)**. Western blot analysis corroborated the KEGG enrichment data **(Figure S5A)**, demonstrating that MTZ intervention suppressed TNF signaling while simultaneously upregulating FOXO signaling. Notably, the TNF signaling pathway acts as a core proinflammatory axis that exacerbates inflammatory responses, disrupts immune homeostasis, and ultimately impairs tissue repair and bone regeneration 60. Conversely, FOXO signaling serves as a key regulator of antioxidant effects, anti-inflammatory processes, and tissue regenerative activities, which play an indispensable role in maintaining cellular redox balance and facilitating effective bone defect healing 61. The simultaneous regulation of these two critical pathways by MTZ is therefore highly conducive to reshaping the immune microenvironment and promoting tissue regeneration. Subsequently, genes associated with antioxidation, tissue repair, and inflammation were chosen for additional analysis. As shown in the heatmap **(Figure S5B)**, MTZ significantly upregulates antioxidant- and tissue repair-related gene expression while notably inhibiting proinflammatory factor expression, thereby remodeling the inflammatory microenvironment and creating favorable immunomodulatory conditions for bone tissue regeneration. In conclusion, the multifunctional MTZ therapeutic nanoplatform demonstrated potent anti-inflammatory and antioxidant properties and suppressed the exaggerated inflammatory response by reshaping the immune microenvironment and modulating multiple key signaling pathways, suggesting its potential benefits for diabetic bone defect treatment.

### Preparation and characterization of hybrid hydrogels

**Figure 4:**
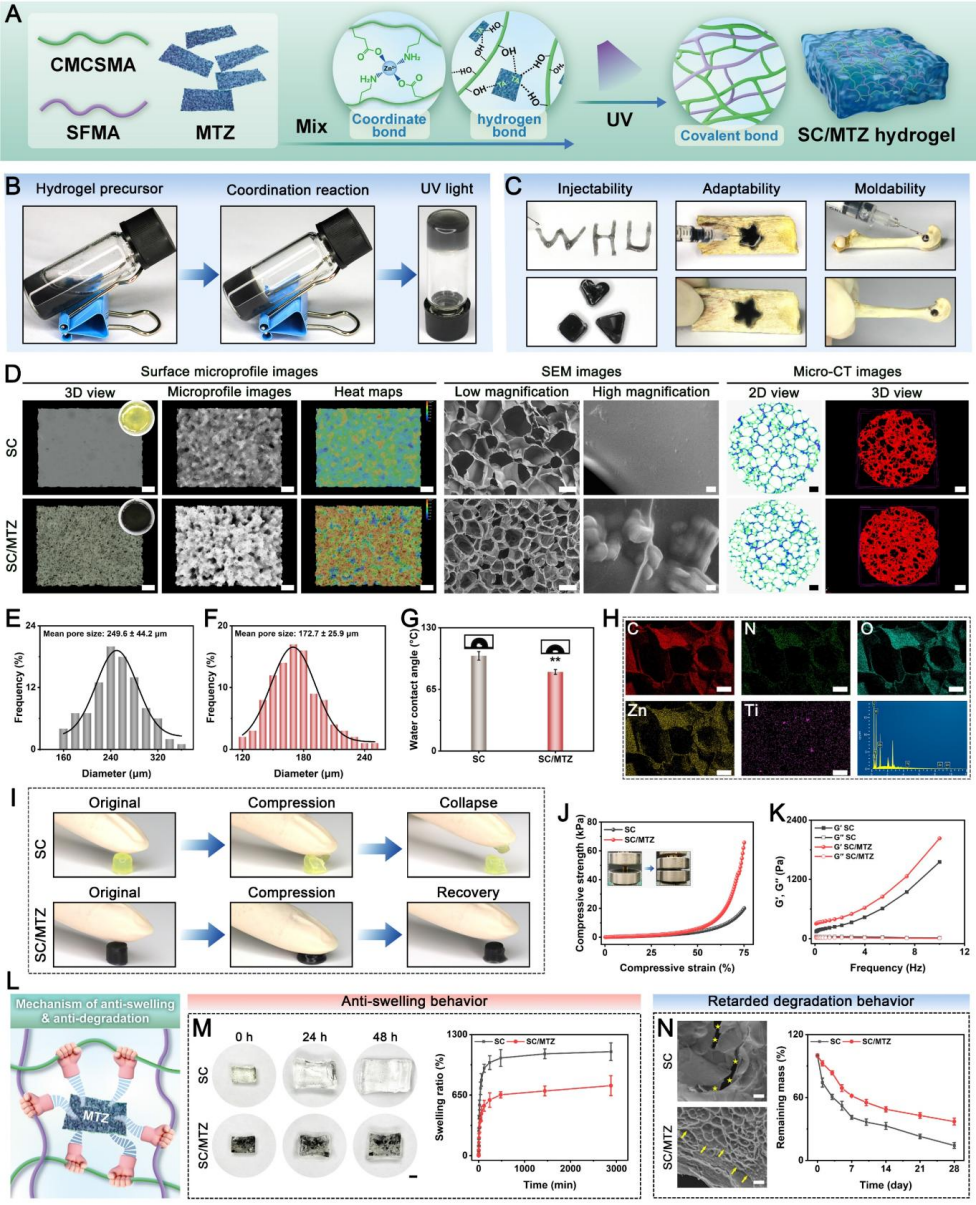
** Characterization of the hybrid hydrogels. (A)** Schematic illustration of the SC/MTZ preparation process. **(B)** Photograph showing the hydrogel gelation process of the SC/MTZ hydrogel, including the coordination reaction and photo-crosslinking process. **(C)** Photographs illustrating the hydrogel's injectability, adaptability, and moldability. **(D)** Surface microprofile, SEM, and micro-CT images of the hydrogels. Scale bar: 200 μm (surface microprofile images, low-magnification SEM images, and 2D micro-CT images), 1 μm (high-magnification SEM images), and 400 μm (3D micro-CT images). **(E-F)** Pore size distributions of the SC and SC/MTZ hydrogels. **(G)** Water contact angles of the hydrogels. **(H)** EDS elemental mapping images of the SC/MTZ hydrogel. Scale bar: 100 μm.** (I)** Digital photos of the hydrogels for standing compression of deformation. **(J-K)** Compressive stress-strain curves and rheological properties of the hydrogels. **(L)** Schematic diagram illustrating the enhanced mechanical, swelling, and degradation properties of the hydrogels. **(M)** Photographs before and after swelling of the hydrogels and the corresponding swelling curves. Scale bar: 1 mm. **(N)** Photographs before and after degradation of the hydrogels and corresponding degradation curves. Scale bar: 25 μm. Data are expressed as the mean ± SD (n = 3). *P < 0.05 and **P < 0.01 indicate significant differences compared with the SC group.

The direct application of nanomaterials to bone defect sites often results in poor retention and variable therapeutic outcomes due to their *in vivo* diffusion and instability. To increase the therapeutic effectiveness of MTZ heterojunctions and support diabetic bone regeneration, we employed an injectable, photocurable hydrogel system. This optimized platform integrates MTZ heterojunctions to counteract the adverse pathological microenvironment and facilitate tissue regeneration **(Figure 4A)**. Selecting suitable hydrogel matrices is crucial for treating bone defects, as non-biodegradable materials can hinder bone healing and increase inflammation. In this work, SFMA and CMCSMA were prepared and functioned as part of the hydrogel matrix to create a biomimetic 3D environment owing to their exceptional biocompatibility and biodegradability 62, 63. In both SFMA and CMCSMA, C=C bonds are introduced to the polymer chain via the grafting of methacrylate groups, which can serve as macromolecular cross-linkers to form C-C bonds through free radial polymerization in the presence of the photoinitiator LAP and UV light (405 nm). Proton nuclear magnetic resonance (^1^H NMR) analysis **(Figure S6)** confirmed the methacrylation reaction by identifying vinyl double bonds (-C=CH_2_) in both SFMA and CMCSMA. Thus, the functionalized SFMA and CMCSMA provided two polymeric matrices for hydrogel preparation. The biomimetic hydrogel system, named SC, was constructed by photo-crosslinking SFMA and CMCSMA. Nonetheless, the practical use of SC-based hydrogels is limited by their inadequate mechanical strength and biological performance. To enhance bone defect healing in diabetic conditions by modulating the inflammatory microenvironment, MTZ heterojunctions with multifunctional biological activity were integrated into a hydrogel network. This integration formed a dual-crosslinked network via photo-crosslinking and metal-ion coordination, resulting in a synergistic effect. During gelation, dynamic, reversible covalent crosslinking occurred via coordination interactions between the functional groups of CMCSMA (-COOH, -NH2, and -OH) and the Zn^2+^ ions in MTZ. The sol-to-gel transition of the injectable hydrogel was partially initiated by MTZ, which served as a crosslinking point via metal coordination between the carboxyl groups in the CMCSMA network and the Zn^2+^ in MTZ **(Figure 4A)**. The phenolic hydroxyl groups on the MTZ heterojunctions facilitate hydrogen bonding with CMCS amino groups, strengthening the hydrogel network.

During preliminary preparation of the SC/MTZ hydrogel, gelation and color transition occurred simultaneously and rapidly **(Figure 4B and Figure S7)**. The hydrogel was initially placed in a glass vial, allowed to gel at 37 °C, and then irradiated with UV light to form a double-network structure. The incorporation of MTZ heterojunctions altered the hydrogel precursor solution from colorless and transparent to black as it transitioned from a liquid to a gel state. The SC/MTZ hydrogel exhibited a rapid sol-gel transition, achieving gelation within 30 s, thereby enhancing its suitability for practical applications. The rapid formation of stable hydrogels is primarily due to the creation of photo-triggered covalent bonds and dynamic metal coordination bonds. Radical polymerization of SFMA and CMCSMA produced a stable gel state for the SC hydrogel. The hydrogel's stability and mechanical performance are enhanced by extensive hydrogen bonding of TA and coordination interactions between Zn^2+^ and CMCSMA, which form a dense dual-crosslinked network and increase crosslinking density. An optimal hydrogel for clinical bone defect treatment should adapt to irregular defect sites and integrate securely with bone tissue within a dynamic *in vivo* microenvironment to achieve therapeutic outcomes 64. **Figure 4C** illustrates that the SC/MTZ hydrogel can be easily extruded through a needle to create customizable patterns and injected into irregularly shaped bone defects, demonstrating its excellent injectability, machinability, and moldability, which aid in surgical procedures. Furthermore, the hydrogel was gelatinized *in situ* within the irregular bone defect. The ability of the hydrogel to be injected into irregular bone cavities and self-cure *in situ* supports personalized repair strategies for extensive bone defects. Even under wet conditions with strong water flushing, the in situ-formed hydrogel still maintained intimate contact with the adjacent bone tissue, preserving its structural integrity and functional adaptability for minimally invasive *in vivo* bone defect repair **(Movie S1)**. The excellent adhesion properties of the SC/MTZ hydrogel under wet conditions can be attributed to its abundant catechol moieties, similar to those in mussel adhesion proteins, which facilitate π-π stacking, cation-π interactions, hydrogen bonding, metal-ligand bonding, and Michael addition 62. This strong interfacial bonding ensures attachment to irregular tissue surfaces in bone defects, effectively preventing post-injection leakage or displacement, which is a critical feature for clinical applications requiring mechanical stability.

Following the successful preparation of the SC/MTZ hydrogel, a systematic investigation of its structural and physical characteristics was conducted. As illustrated in **Figure 4D**, both the SC and SC/MTZ hydrogels exhibited loose, porous, honeycomb-like structures after freeze-drying. The synergistic crosslinking of UV light and MTZ in the polymer matrix resulted in smaller pore sizes in SC/MTZ compared to SC **(Figure 4E-F)**. SEM images and statistical analysis indicate that increased crosslinking network density in the hydrogel results in smaller pore sizes by reducing the free water volume. Although the porosity of the SC/MTZ hydrogels was lower than that of the SC hydrogels, it still exceeded 75% **(Figure S8)**. Micro-CT analysis and pseudo-color images demonstrated that incorporating MTZ resulted in a denser microstructure in the synthesized SC/MTZ hydrogel, characterized by highly interconnected pores. Compared with the SC group, the SC/MTZ group showed denser pores and rougher surfaces, characterized by uniformly distributed, clustered protrusions that were likely composed of MTZ heterojunctions. The uniform and interconnected porous structure, along with appropriate porosity and a rough surface, provides an optimal 3D microenvironment for cell adhesion, proliferation, osteogenic differentiation, and new bone formation. Similarly, the addition of the MTZ heterojunction slightly increased the hydrophilicity of the hydrogel, which was due to the successful decoration of the hydrophilic TA coating **(Figure 4G)**. Enhancing hydrophilicity in tissue engineering materials benefits bone repair by promoting the adhesion and proliferation of osteogenic cells and facilitating interactions between the implant surface and surrounding host tissue 65. EDS elemental mapping images indicated a consistent distribution of C, O, N, Zn, and Ti within the SC/MTZ hydrogel network **(Figure 4H)**, confirming the even incorporation of MTZ heterojunctions. Hence, the prepared SC/MTZ hydrogels with well-defined porous microstructures, increased crosslinking density, surface roughness, and hydrophilicity hold great potential for tissue engineering applications.

In addition to the previously mentioned characteristics, the capacity of hybrid hydrogels to withstand compression and maintain structural integrity is crucial for bone repair following extended implantation 66. The uniform dispersion and crosslinking reinforcement of the MTZ heterojunction within the hydrogel facilitate stress transfer along the SC/MTZ interface **(Figure 4I)**. The incorporation of the MTZ heterojunction enhanced the compressive strength of the hydrogels from approximately 17.8 to 63.8 kPa, as shown in **Figure 4J and Figure S9**. The improved mechanical strength is crucial for ensuring the structural stability and support of the prepared hydrogels in bone repair. Moreover, the SC/MTZ hydrogel recovered quickly to its original shape without structural failure upon withdrawal of compression force, indicating stable elasticity and toughness. On the other hand, the SC hydrogel's single photo-crosslinking grid structure is consistently brittle and weak due to its uneven cross-linking density and numerous entanglements. Incorporating MTZ into the SC hydrogel enhances crosslinking, leading to greater mechanical stress in the SC/MTZ hydrogel than in the pure SC hydrogel. The improved structural stability of SC/MTZ hydrogels is crucial for bone repair, as it prevents their displacement due to intraoperative bleeding and diffusion beyond the defects 67. Despite SC's limited mechanical strength for load-bearing bone repair, the integration of inorganic MTZ nanosheets significantly improves its structural stability. The mechanical properties of the SC/MTZ hydrogel are adequate for temporary structural support in bone regeneration, as the diabetic bone defect model in this study represents a non-load-bearing or partially load-bearing scenario 64. The rheological properties of the hydrogels were examined to validate the compressive test data **(Figure 4K)**. The results indicated that, compared with SC hydrogels, SC/MTZ hydrogels exhibited higher G' values across the measured frequency range (0.1-10 Hz), suggesting greater hardness and improved structural integrity due to the inclusion of MTZ heterojunctions. This improvement in mechanical performance was attributed to multiple interactions, including metal-ion coordination bonds, photo-triggered covalent bonds, and hydrogen bonds. The introduced MTZ heterojunction could serve as a crosslinking site, ultimately improving the mechanical properties of the material.

The swelling behavior and degradation of different hydrogels were subsequently evaluated. **Figure 4M** shows that all composite hydrogels exhibited notable swelling, reaching equilibrium after 48 h in PBS. Owing to the dynamic crosslinking of Zn^2+^ or Ti^3+^ from MTZ with adjacent functional groups (-COOH, -NH_2_, and -OH), the SC/MTZ hydrogel reached equilibrium more quickly. It exhibited lower swelling ratios than did the single photo-crosslinked SC hydrogel. The SC/MTZ hydrogel achieves enhanced structural stability under physiological conditions through a dual crosslinked network formed by photo-crosslinking and MTZ-triggered ionic crosslinking **(Figure 4L)**. A lower swelling rate ensures that the SC/MTZ hydrogel volume remains relatively stable after water absorption, minimizing the risk of detachment due to expansion, which is essential for bone defect repair. Conversely, a reduced swelling rate helps preserve the structural integrity and mechanical strength of hydrogels, indicating an improvement in the hydrogel network 62. Biodegradable hydrogels are metabolized and absorbed by human tissues during bone healing, facilitating the ingrowth of blood vessels and bone tissue. This degradability eliminates the need for secondary surgery to remove the stent or implant, thereby minimizing additional surgical interventions for patients, which is crucial for clinical applications. More importantly, the use of biodegradable bone repair scaffolds can also reduce the incidence of adverse events, eliminate patient discomfort, and support tissue ingrowth and bone repair 68. Consequently, the biodegradation of synthesized hydrogels is crucial for bone healing and tissue regeneration. This study examined the degradation of all hydrogels in a PBS solution at pH 7.4, simulating a biological system with a collagenase solution **(Figure 4N)**. All hydrogels demonstrated notable degradation when exposed to collagenase, albeit at varying rates. Compared with the SC hydrogel, the SC/MTZ hydrogel has a slower degradation rate due to its high crosslinking density and single enzymatic hydrolysis mode. The rapid degradation of SC hydrogels is due to their low crosslinking density and weak single crosslinking from photo-polymerization, which make them more vulnerable to collagenase and hasten the breakdown of the hydrogel network. The SC/MTZ hydrogel initially degraded slowly due to its stable double crosslinking, but after 2 weeks, the degradation rate increased significantly as the hydrogel structure began to break down alongside the degradation of MTZ. Despite this, the SC/MTZ degradation rate remains significantly lower than that of SC hydrogels, effectively preserving structural integrity and ensuring subsequent therapeutic applications. Metal ions, specifically Ti^3+^ or Zn^2+^, in MTZ heterojunctions within the hydrogel can form coordination complexes with CMCSMA. This is demonstrated by the dense metal-phenolic network film on the SC/MTZ hydrogel surface, which inhibits medium solution permeation into the hydrogel network. In contrast, large cracks and fragmentation appeared on the pore walls of the SC hydrogel, as evidenced by SEM analysis. This degradation behavior is advantageous for bone regeneration, as it allows a temporary scaffold to preserve initial mechanical integrity while being progressively replaced by new bone tissue. Gradual degradation supports the dynamic creation of space for stem cell migration, tissue infiltration, and neovascularization 9. The SC/MTZ hydrogel exhibits promising potential for bone regeneration due to its advantageous properties, including delayed enzymatic degradation, controlled swelling, excellent injectability, and robust mechanical resilience. These characteristics make it suitable for minimally invasive *in situ* implantation at irregular defect sites, highlighting its significant clinical applicability.

### Photothermal, stimulus-responsive, antioxidant, and antimicrobial properties of hybrid hydrogels

**Figure 5:**
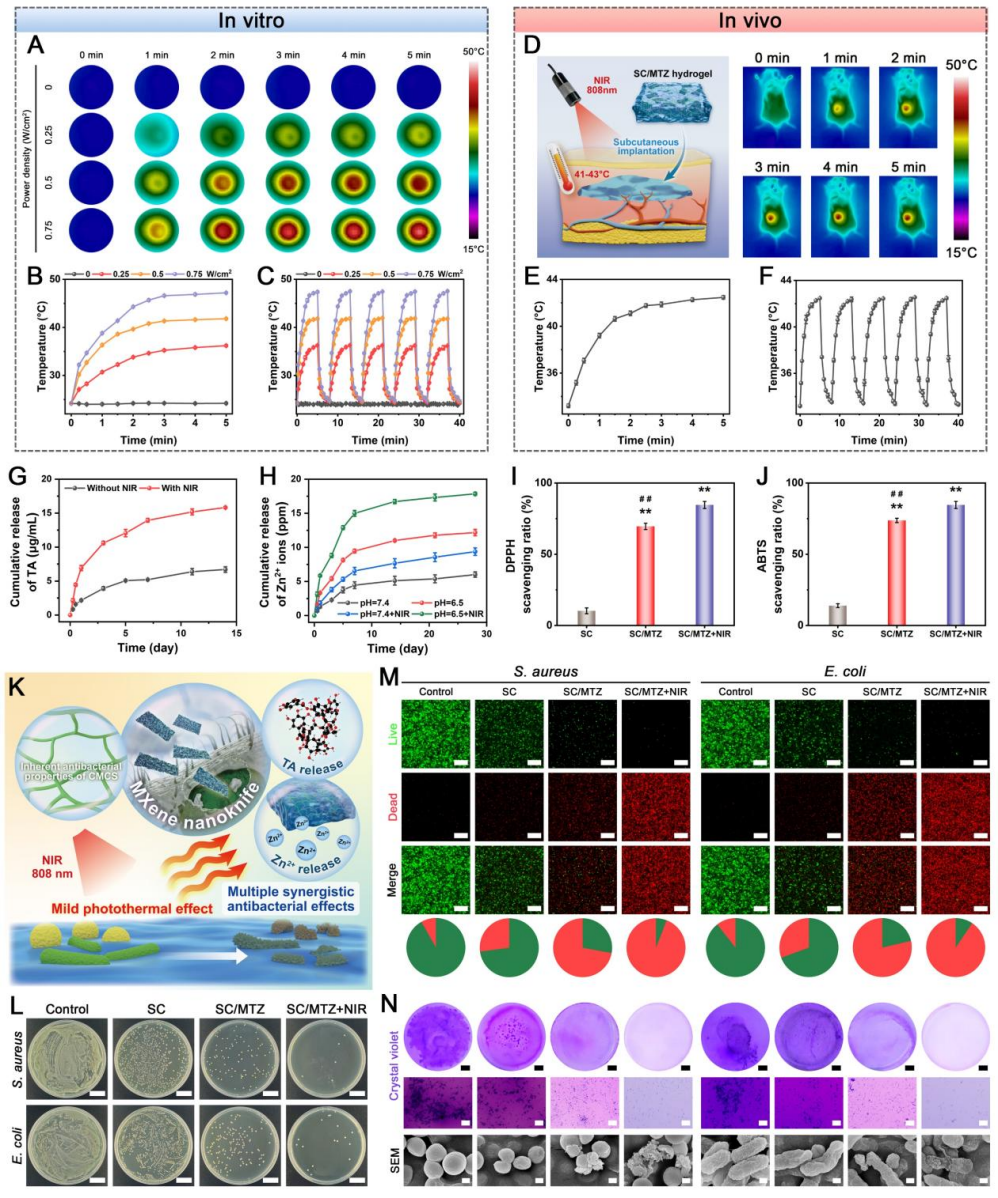
** Photothermal, antioxidant, and antibacterial performance of the hybrid hydrogels. (A-B)** Real-time heating curves and infrared thermographic images of SC/MTZ were recorded at varying power densities (0, 0.25, 0.5, and 0.75 W/cm²) over a duration of 5 min. **(C)** The photothermal stability of SC/MTZ was assessed across varying power densities (0, 0.25, 0.5, and 0.75 W/cm^2^) over five cycles of laser activation and deactivation. **(D-E)** Representative photographs and thermal infrared images of rat dorsal skin treated with SC/MTZ under 808 nm laser irradiation (0.5 W/cm^2^, 5 min). **(F)** The photothermal stability of SC/MTZ was evaluated under 808 nm NIR irradiation at 0.5 W/cm² over five cycles of laser on/off. **(G)** Cumulative TA release from SC/MTZ with or without periodic 808 nm NIR irradiation at 0.5 W/cm². **(H)** Cumulative Zn^2+^ ion release from SC/MTZ was evaluated at pH values of 6.5 and 7.4, both with and without periodic NIR irradiation (808 nm, 0.5 W/cm^2^). **(I-J)** Quantitative analysis of the DPPH and ABTS free radical scavenging efficiency of the hydrogels. **(K)** Schematic diagram of the photothermal antibacterial performance. **(L)** Photographs of surviving bacterial clones on agar plates in different treatment groups. Scale bar: 2 cm. **(M)** Images showing live/dead fluorescence staining of *S. aureus* and *E. coli* across various treatment groups. Scale bar: 50 μm. **(N)** Crystal violet and SEM images of *S. aureus* and *E. coli* across various treatment groups. Scale bar: 2 mm (low-magnification crystal violet staining images), 50 μm (high-magnification crystal violet staining images), and 250 nm (SEM images). Data are expressed as mean ± SD (n = 3). Significant differences compared to the SC group are indicated by *P < 0.05 and **P < 0.01. Significant differences compared to the SC/MTZ+NIR group are indicated by ^#^P < 0.05 and ^# #^P < 0.01.

Superior photothermal capabilities can precisely regulate local temperatures, eliminating bacteria while preserving healthy tissues. Local mild hyperthermia therapy (40-43 °C) enhances immune activation, boosts local blood flow and oxygen levels, and accelerates MSC differentiation, osteoblast maturation, and mineralization, facilitating new bone and blood vessel formation during bone repair 12. The photothermal conversion capability of the SC/MTZ hydrogel in PBS was assessed under 808 nm NIR laser irradiation for 5 min at varying power densities **(Figure 5A)**. The temperature changes in the samples were then monitored and recorded via an infrared thermal imager. With prolonged irradiation, the temperature increased rapidly to 35-50 °C. This phenomenon is due to the effective photothermal conversion capability of MTZ heterojunctions. Moreover, the temperature increase was found to be correlated with irradiation power and time, thereby enabling precise temperature regulation during hyperthermia therapy. The SC/MTZ hydrogel showed no notable temperature rise without NIR irradiation. Following 3 min of NIR light exposure at power densities of 0.25, 0.5, and 0.75 W/cm², the temperature of SC/MTZ quickly increased to 35.2 °C, 41.3 °C, and 46.6 °C, respectively, before stabilizing at a plateau **(Figure 5B)**. Moreover, the temperature cycle profiles did not change significantly after five cycles of NIR laser on/off irradiation **(Figure 5C)**, demonstrating good photothermal stability. The *in vivo* photothermal efficacy of the sample, which was implanted in rat skin for 14 days, was assessed under 808 nm NIR radiation at a power density of 0.5 W/cm². As shown in **Figure 5D-E**, the local temperature in the implanted area increased to 42 ± 1 °C within 5 min, similar to the trend observed *in vitro*. Additionally, the SC/MTZ hydrogel system demonstrated consistent photothermal efficacy across five consecutive laser on/off cycles **(Figure 5F)**, affirming its feasibility and stability for mild PTT *in vivo*. It has been demonstrated that the maintenance of an appropriate thermal environment for bone defect healing may be of critical importance with respect to the regulation of the autoimmune system and facilitation of bone tissue regeneration 69. However, excessive temperatures above 45 °C may also cause thermal damage to local tissues and induce potential thermotoxicity at the cellular level. To balance biosafety and photothermal characteristics, this study targeted a mild photothermal therapy temperature of 42 ± 1 °C using a laser with a power density of 0.5 W/cm². In summary, the SC/MTZ hydrogels exhibit excellent photothermal conversion efficiency, meeting the performance requirements for photothermal antibacterial therapy and mild hyperthermia during tissue repair and regeneration.

The stimuli-responsive release characteristics of the SC/MTZ hydrogel were evaluated under NIR irradiation. As depicted in **Figure 5G**, SC/MTZ exhibited rapid TA release under NIR irradiation. NIR-induced elevated temperatures disrupt the π-π stacking of noncovalent bonds between aromatic hydrocarbons in the substrate and the TA coating, leading to rapid TA release upon NIR laser stimulation 70. Considering the antioxidant and anti-inflammatory properties of TA, it is hypothesized that NIR-triggered TA release under diabetic conditions reduces ROS, alleviates oxidative stress and inflammation, and promotes macrophage reprogramming during the initial phase of the inflammatory response after bone injury. Bone injury creates a pathological microenvironment with a slightly acidic pH, which contrasts with the pH of healthy bone tissue, which is 7.3-7.4 71. The acidic microenvironment specifically suppresses ALP expression and increases osteoclast activity, hindering BMSC osteogenic differentiation and worsening bone resorption 72. Therefore, to improve the acidic microenvironment for optimal biological performance and tissue regeneration, an intelligent stimuli-responsive hydrogel platform is engineered and encapsulated with MTZ heterojunctions for NIR/pH-responsive Zn^2+^ release. **Figure 5H** illustrates the Zn^2+^ stimuli-responsive drug release behavior under varying pH conditions, both with and without NIR laser irradiation. The Zn^2+^ release rate from the SC/MTZ hydrogel was notably faster under acidic conditions (pH 6.5) and higher temperatures than in a neutral environment (pH 7.4). The most significant release occurred under combined acidic and NIR stimuli (pH 6.5 + NIR), demonstrating a controllable release mechanism responsive to both pH and NIR. After bone injury, the SC/MTZ intelligently released Zn^2+^ in response to both the weakly acidic environment and elevated temperature induced by the NIR light. This smart-responsive release mechanism was achieved by rupturing the metal coordination bonds between Zn^2+^ and CMCSMA under acidic conditions. Moreover, the pH-responsive release characteristics of ZIF-8 play a role in this process 20. The controlled release of Zn^2+^ and TA creates an anti-inflammatory, pro-healing environment that effectively induces macrophage M2 polarization and functional cytokine secretion. This process synergistically enhances the recruitment of endogenous BMSCs and promotes angiogenesis, which is supported by mild heat stimulation. The observed release profiles effectively ensure prompt inflammation suppression and immune regulation in the early stages of bone repair, facilitating accelerated bone regeneration under diabetic inflammatory conditions.

Excessive ROS accumulation and resulting oxidative stress damage are strongly linked to impaired bone regeneration. In the pathological state of diabetes, chronic hyperglycemia leads to excessive ROS production. This oxidative stress environment has many adverse effects on bone healing, including cellular dysfunction, osteogenic inhibition, vascular deterioration, and ECM disruption 3. Advanced bone repair scaffolds with antioxidant capabilities can efficiently neutralize ROS and reduce oxidative damage, thereby enhancing diabetic bone healing. This study employed DPPH and ABTS radical scavenging assays to assess the *in vitro* ROS scavenging capacity of the hydrogels. **Figure 5I** illustrates that the SC/MTZ hydrogel exhibits a higher DPPH scavenging ratio than the SC hydrogel, which is attributed to the antioxidative properties of MXene@TA. The abundant phenolic hydroxyl groups in TA and the excellent electron-conducting properties of MXene could impart outstanding antioxidant properties to the SC/MTZ hydrogel. Compared with the hydrogel alone, the SC/MTZ hydrogel, when combined with gentle NIR stimulation, exhibited enhanced antioxidative ability. This improvement is likely due to the accelerated disassembly of MTZ nanosheets, which exposes more active sites for interaction with free radicals via electron transfer reactions. The NIR-triggered release of TA from the SC/MTZ hydrogel provides hydrogen atoms, disrupting free radical chain reactions and providing antioxidant and free radical-scavenging effects. Compared with the SC and SC/MTZ hydrogel-treated groups, the SC/MTZ+NIR group presented a much greater DPPH scavenging efficiency (84.6 ± 2.5%). The photoactivated SC/MTZ hydrogel system consistently demonstrated excellent ABTS radical-scavenging activity. As shown in **Figure 5J**, the SC and SC/MTZ hydrogels had scavenging ratios of ≈13.9 ± 1.3% and 73.8 ± 1.5%, respectively. The SC/MTZ+NIR group demonstrated a significantly higher ABTS scavenging efficiency (84.9 ± 1.63%) due to mild photothermal effects, highlighting the exceptional radical scavenging capability of the hydrogel system. The SC/MTZ hydrogel exhibited enhanced radical scavenging under mild NIR irradiation, driven by the combined effects of mild heat stimulation and MTZ, making it a promising antioxidant platform for neutralizing oxidative stress at bone injury sites.

Bacterial infections during bone repair can intensify initial inflammation at the defect site, hindering tissue healing 46. The therapeutic SC/MTZ hydrogel system afforded localized delivery of the antimicrobials TA and Zn^2+^ as well as the functionalized MXene nanosheets, which is anticipated to confer antibacterial effects under NIR irradiation **(Figure 5K)**. Motivated by the promising NIR photothermal properties and stimuli-responsive release of TA and Zn^2+^, we investigated the antimicrobial effects of this combination therapy using *S. aureus* and *E. coli* as model organisms, given their relevance in bone injury-related infections. **Figure 5L** shows that the SC hydrogel-treated group exhibited antibacterial activity, attributed to chitosan's retention of amino groups, which have antibacterial properties. Doping multifunctional MTZ heterojunctions enhanced the antibacterial efficacy of SC/MTZ against *S. aureus* and *E. coli*, as evidenced by a significant increase in the antibacterial ratio **(Figure S10)**. This improvement is attributed to the sustained release of TA and Zn^2+^. Nonetheless, some bacteria persisted. Upon mild NIR irradiation, the SC/MTZ hydrogel exhibited enhanced inhibition ratios against both *S. aureus* and *E. coli*, demonstrating superior antibacterial activity compared with the other groups. While the NIR-induced mild photothermal effect alone does not fully eradicate bacteria, it can enhance the activity of metal ions (e.g., Zn^2+^) through the 'mild thermionic effect', leading to efficient, rapid, and sustained bacterial inhibition 73. The mild thermal effect on cell membrane permeability, combined with the nanoknife effect of MXene nanosheets, can enhance the uptake of ions and small molecules such as TA, thus promoting effective antibacterial therapy. The live/dead bacterial staining results aligned with the quantitative antibacterial findings **(Figure 5M)**, underscoring the enhanced antibacterial effectiveness of the SC/MTZ+NIR group. It is worth noting that combined with the results of the stimulus-responsive release test, this antibacterial activity was anticipated to be particularly significant in a diabetic, weakly acidic environment.

Following the initial antibacterial experiment results, SEM was used to examine bacterial morphology post-treatment. **Figure 5N** shows that the bacteria in the control group exhibited a smooth, complete surface morphology. The surface of bacteria treated with SC showed slight collapse and shrinkage, suggesting that the cationic amino groups disrupted the cell membrane's stability. In contrast, the bacteria treated with the SC/MTZ hydrogel exhibited significant cellular damage, especially in the SC/MTZ+NIR group. When combined with mild NIR irradiation, the SC/MTZ hydrogel caused substantial damage to and distortion of the cell membranes of *S. aureus* and *E. coli*, resulting in membrane rupture and leakage of the cellular contents. SEM observations confirmed that the hydrogel treatment exhibited antibacterial activity by damaging the membrane. Among them, the damage caused by SC/MTZ+NIR to bacteria was particularly significant, which verified the results of the antibacterial test. This explains the notably reduced bacterial count observed in the SC/MTZ and SC/MTZ+NIR groups. This result underscores the synergistic effect of MTZ and NIR light on bacterial eradication. This suggests that mild heat stimulation causes TA and Zn^2+^ release, thereby increasing the antibacterial ability of MTZ while simultaneously reducing the incidence of toxic side effects. Bacteria tend to accumulate on the implant surface and form biofilms, further leading to chronic infections that are difficult to eradicate 7. The effectiveness of the hydrogel in disrupting biofilms was assessed for both *S. aureus* and *E. coli* **(Figure 5N)**. Not surprisingly, the SC/MTZ+NIR group showed the lowest biofilm biomass, indicating its exceptional efficacy at disrupting bacterial biofilm structure. When *E. coli* was subjected to the same treatment method, a significant inhibitory effect on biofilm formation was also observed **(Figure S11)**, indicating the strongest biofilm-disrupting ability. Natural phenolic compounds (i.e., TA) and metal ions (i.e., Zn^2+^) have been identified as potent anti-biofilm agents with biofilm-penetrating capabilities and intrinsic bactericidal performance 24. Notably, in addition to TA and Zn^2+^, the Ti_3_C_2_T_x_ MXene nanosheets likely contributed to the robust antibacterial properties due to their well-established nanoknife effects 74. Moreover, mild-temperature PTT enhances cell membrane permeability by increasing lipid and protein fluidity and mobility at elevated temperatures (Figure 5K), thereby improving the antibacterial efficacy of these components 73. This synergistic sterilization mechanism enhances the antibacterial effectiveness of the material and minimizes the risk of antimicrobial resistance, offering crucial support for large-scale bone defect repair in diabetic conditions.

### *In vitro* biocompatibility and cell adaptability of the hybrid hydrogels

**Figure 6:**
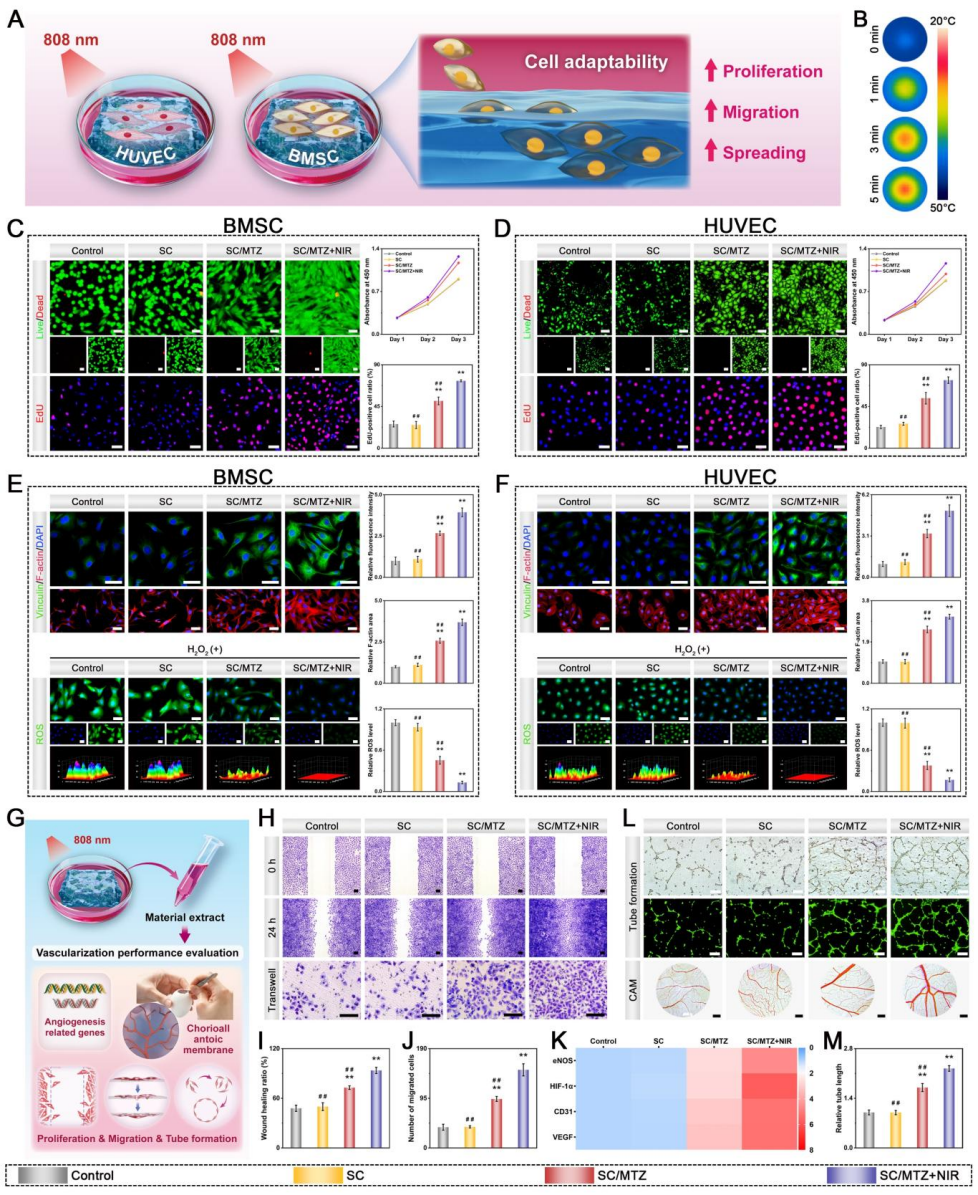
***In vitro* cytocompatibility and angiogenic evaluation of the hydrogels. (A)** Schematic illustration of the coculture system subjected to periodic NIR irradiation. **(B)** Real-time infrared thermal imaging of cell/hydrogel coculture complexes was conducted under NIR irradiation at 808 nm and 0.5 W/cm² for 5 min. **(C-D)** Representative images and quantitative analysis of live/dead staining and EdU assays across various treatment groups. Scale bar: 50 μm. **(E-F)** Representative images and quantitative analyses of Vinculin, F-actin, and DCFH-DA staining across various treatment groups. Scale bar: 50 μm.** (G)** Schematic diagram of the impact of the hydrogel on cell migration and angiogenesis. **(H-J)** Representative images and quantitative analysis of HUVEC migration and Transwell assays across various treatment groups. Scale bar: 100 μm.** (K)** Heatmap of angiogenesis-related marker gene expression.** (L)** Representative images of HUVEC tube formation and CAM assays in different treatment groups. Scale bar: 200 μm (tube formation assay) and 4 mm (CAM assay).** (M)** Relative tube length in other treatment groups. Data are expressed as the mean ± SD (n = 3). Significant differences compared to the SC group are indicated by *P < 0.05 and **P < 0.01. Significant differences compared to the SC/MTZ+NIR group are indicated by ^#^P < 0.05 and ^# #^P < 0.01.

The effectiveness of new biomaterial scaffolds in enhancing tissue regeneration is influenced by their antibacterial properties, cellular compatibility, and adaptability, which can be evaluated by measuring the cellular response to the engineered hybrid hydrogel **(Figure 6A)**. The *in vitro* cytocompatibility of the hydrogels was assessed, as shown in **Figure 6C-D**. After several days of incubation with the hydrogels, all groups demonstrated high cell viability and proliferation. The results of the CCK-8 assay showed that the proliferation of BMSCs and HUVECs cocultured with the hydrogels was similar on the first day across all groups. After coculturing for 3 days, cell proliferation rates were significantly higher in the MTZ-contained hydrogel groups than in the control and SC groups. The effects of mild heat stimulation on the cells were assessed separately. **Figure 6B-D** demonstrates that daily NIR irradiation (808 nm, 0.5 W/cm^2^, 5 min) significantly enhanced the growth and survival of the BMSCs and HUVECs cocultured with the SC/MTZ hydrogel. **Figure 6C-D** illustrates that the SC/MTZ+NIR group exhibited the highest degree of cell proliferation, followed by the SC/MTZ group, after 1 and 3 days of incubation. In contrast, the control and SC groups showed minimal changes in living cell density. An EdU staining assay showed that the SC/MTZ hydrogel group had a greater number of EdU-positive cells than the control and SC groups, particularly under mild thermal stimulation. The SC/MTZ+NIR group exhibited enhanced cell survival, growth, and proliferation, driven by the combined effects of MTZ heterojunctions and mild PTT on cellular function. Moreover, on-demand and periodic mild hyperthermia is recognized as a supportive therapy for bone healing, as numerous studies have confirmed its role in promoting the growth and osteogenic differentiation of osteogenesis-related cells 75. To further illustrate cell attachment and spreading on the hydrogels, immunofluorescence staining of focal adhesion protein (vinculin) and F-actin staining were conducted after 3 days.

As displayed in **Figure 6E-F**, the cells cultured on the pure SC surface exhibited restricted spreading with limited pseudopodia formation, suggesting a compromised cell adhesion capability. The cells adhering to the SC/MTZ hydrogel exhibited enhanced spreading morphology, with organized cytoskeletal structures and increased vinculin fluorescence intensity, suggesting improved cell spreading and focal adhesion formation. MTZ heterojunctions with catechol groups can bind to integrin receptors on cell membranes, providing numerous adhesion sites that enhance cell adhesion and proliferation. The controlled and sustained release of bioactive Zn^2+^ ions enhances cell adhesion, proliferation, and differentiation 21. This improvement was even more pronounced when the cells were simultaneously exposed to mild NIR irradiation on the SC/MTZ hydrogel. Similar results were observed in HUVEC assays, further corroborating the excellent bioactivity of the SC/MTZ hydrogel under NIR irradiation. Cell adhesion and affinity in the SC/MTZ hydrogel are due to catechol groups and Zn^2+^ release, which promote a well-developed cytoskeleton and numerous adhesive spots. Furthermore, this organized F-actin cytoskeleton and abundant organelles, called focal adhesions (FAs), have been shown to reflect a favorable microenvironment that supports osteogenic function 76. The SC/MTZ hydrogel significantly enhanced cell adhesion, proliferation, and spreading under optimized NIR parameters. The improved cell adhesion is due to the MTZ heterojunctions offering extra adhesion sites. Quantitative analysis confirmed that the combination of MTZ loading and mild heat enhanced the adhesion and spreading of BMSCs and HUVECs on the hydrogel **(Figure 6E-F)**. Outstanding cytocompatibility is essential for cell adhesion, proliferation, migration, differentiation, and other functional activities 77. These findings underscore the excellent cytocompatibility of the hydrogel, which is crucial for bone healing and tissue repair applications.

Following bone injury, especially under diabetic conditions, abnormal ROS accumulation typically occurs at bone defect sites, leading to a harmful cycle of inflammation and oxidative stress. This cycle results in damage and apoptosis of osteogenesis-related cells, such as BMSCs and HUVECs 78. Compromised neovascularization can severely hinder bone healing and worsen the bone microenvironment. The ability of the hydrogels to reduce excess ROS and enhance vascularization was assessed, as depicted in **Figure 6E-F**. To simulate oxidative stress *in vitro*, BMSCs and HUVECs were treated with H_2_O_2_, and the intracellular ROS levels were measured using DCFH-DA. **Figure 6E-F** shows that the H_2_O_2_-stimulated group without treatment exhibited the highest fluorescence intensity, suggesting elevated intracellular ROS production. The intracellular ROS levels in the SC hydrogel-treated group were comparable to those in the control group, indicating that the hydrogel matrix had a minimal impact on ROS scavenging. The fluorescence intensity was significantly lower in the MTZ-encapsulated hydrogel groups than in the control and SC groups because of the superior ROS scavenging capability of the MTZ nanosheets. Quantitative analysis indicated that cells exposed to the SC/MTZ hydrogel and mild NIR irradiation exhibited the lowest fluorescence intensity, implying that the combined antioxidant approach of MTZ and mild hyperthermia effectively reduced intracellular oxidative stress. These findings indicate that the SC/MTZ hydrogel exhibits strong antioxidant and ROS-scavenging properties, which are expected to reduce apoptotic cell death and dysfunction in diabetic bone defect areas upon mild NIR irradiation.

Revascularization is crucial for skeletal development and bone regeneration, as well as for maintaining intracellular redox balance. Functional vascular networks form around injured areas to transport oxygen and nutrients, aiding the recruitment of osteogenesis-related cells to defect sites 79. Zn^2+^ ions, essential trace elements, significantly contribute to bone formation by affecting immune homeostasis and osteoblast differentiation 77. Recent studies have reported the role of Zn^2+^ ions in promoting angiogenesis. Zn^2+^ ions enhance vascularization by stimulating endothelial cell proliferation and migration and increasing the expression of angiogenic markers like VEGF and Ang-1 20. HUVECs, the most extensively studied cell type for vascularization, were used to assess the angiogenic potential of the hydrogels. The potential of hydrogel extracts from various treatment groups to enhance cell migration and recruitment was assessed via scratch wound healing and Transwell migration assays **(Figure 6G)**. **Figure 6H-J** illustrates that cell migration and wound closure were significantly enhanced in the SC/MTZ hydrogel-treated groups, with the SC/MTZ+NIR group demonstrating the highest proangiogenic activity. Quantitative analysis confirmed that the migratory capacity of HUVECs was significantly enhanced in both the SC/MTZ and SC/MTZ+NIR groups, particularly in the latter, highlighting the superior proangiogenic potential of the SC/MTZ hydrogel with mild NIR irradiation. Immunofluorescence staining and quantitative analysis indicated that the angiogenic proteins CD31 and HIF-1α were expressed at significantly higher levels in the SC/MTZ and SC/MTZ+NIR groups than in the control and SC groups, with the SC/MTZ+NIR group showing the highest expression **(Figure S12)**. The qRT-PCR results corroborated these findings, showing that the SC/MTZ+NIR group had the highest expression levels of eNOS, HIF-1α, CD31, and VEGF **(Figure 6K)**. The increased expression of these markers in the SC/MTZ+NIR group suggests enhanced angiogenic potential, likely facilitating better nutrient and oxygen supply to regenerating bone tissue. The increased expression of HIF-1α and VEGF is essential for endothelial cell proliferation, migration, recruitment, and angiogenesis, underscoring the potential of SC/MTZ to enhance vascularization in bone repair. An *in vitro* tube formation assay was performed to assess the angiogenic potential of HUVECs after incubation with the hydrogel extracts. As shown in **Figure 6L-M**, only incomplete tube formation was observed in both the control and SC groups, indicating that the proangiogenic activity of HUVECs was inhibited. Compared with the other groups, the MTZ-encapsulated hydrogel groups developed a more extensive and denser vessel network **(Figure S13)**, which aligns with findings from the wound healing and Transwell migration assays. The SC/MTZ+NIR group demonstrated the strongest proangiogenic capacity, indicating a synergistic effect between periodic NIR exposure and bioactive components through on-demand mild thermal stimulation. Our findings demonstrate that, even without direct contact in the Transwell coculture system, incorporating MTZ nanosheets significantly enhances HUVEC angiogenesis, resulting in the formation of extensive, interconnected vascular networks. The SC/MTZ+NIR group exhibited superior proangiogenic potential due to the combined effects of sustained Zn^2+^ release from SC/MTZ, facilitated by NIR irradiation. Compared with the SC/MTZ group, the SC/MTZ+NIR group exhibited significantly increased tube numbers and lengths. The above experiments suggested that the SC/MTZ hydrogel could effectively increase the expression levels of angiogenic markers in HUVECs and promote cell migration and angiogenesis under gentle NIR irradiation, thereby potentially accelerating neovascularization during bone repair. To further assess the effects of hydrogel extracts on vascularization, a chorioallantoic membrane (CAM) assay was performed, focusing on the observation of embryonic blood vessels 46. The CAM model recapitulates *in vivo* implantation conditions while being more cost-effective and reproducible than animal experimentation. In this context, fertilized chicken eggs were incubated to develop CAM vasculatures, which were then exposed to various hydrogel extracts. As depicted in **Figure 6L**, in both the control and SC groups, many small-diameter blood vessels were intricately interconnected and converged, forming a dense microvascular network. The MTZ-contained hydrogel group, particularly the SC/MTZ+NIR group, exhibited numerous blood vessels with larger diameters, highlighting the effectiveness of the mild photothermal-assisted SC/MTZ hydrogel in enhancing neovascularization. Quantitative analysis of the blood vessel area revealed significant enhancements upon treatment with the SC/MTZ hydrogel in combination with gentle NIR irradiation **(Figure S13)**. The SC/MTZ+NIR group exhibited enhanced neovascularization, driven by multiple factors. Zn^2+^ ions released from MTZ heterojunctions promote endothelial cell proliferation, migration, and tube formation. Zn^2+^ acts as a cofactor for enzymes that play a role in angiogenesis and directly affects the expression of angiogenic markers. These results align with prior research 16, 75, suggesting that the combined effects of sustained bioactive ion release and mild thermal stimulation promote endothelial cell migration, recruitment, and vascularization. In the initial phase of bone repair, increased vascularization facilitates the influx of immune cells and the generation of proangiogenic cytokines. Achieving optimal neovascularization is crucial for effective tissue integration and regeneration in tissue engineering 80. Furthermore, the vascular system, consisting of large-diameter blood vessels, facilitates the attraction of progenitor cells to injury sites by supplying essential oxygen, nutrients, and growth factors necessary for bone biomineralization and regeneration.

*In vitro* experiments revealed that the photoactivated SC/MTZ hydrogel system significantly improved BMSC and HUVEC attachment, proliferation, spreading, and migration while reducing excess ROS and restoring redox balance, thereby enhancing vascularization and demonstrating substantial potential for bone repair.

### *In vitro* osteogenic potential under oxidative stress

**Figure 7:**
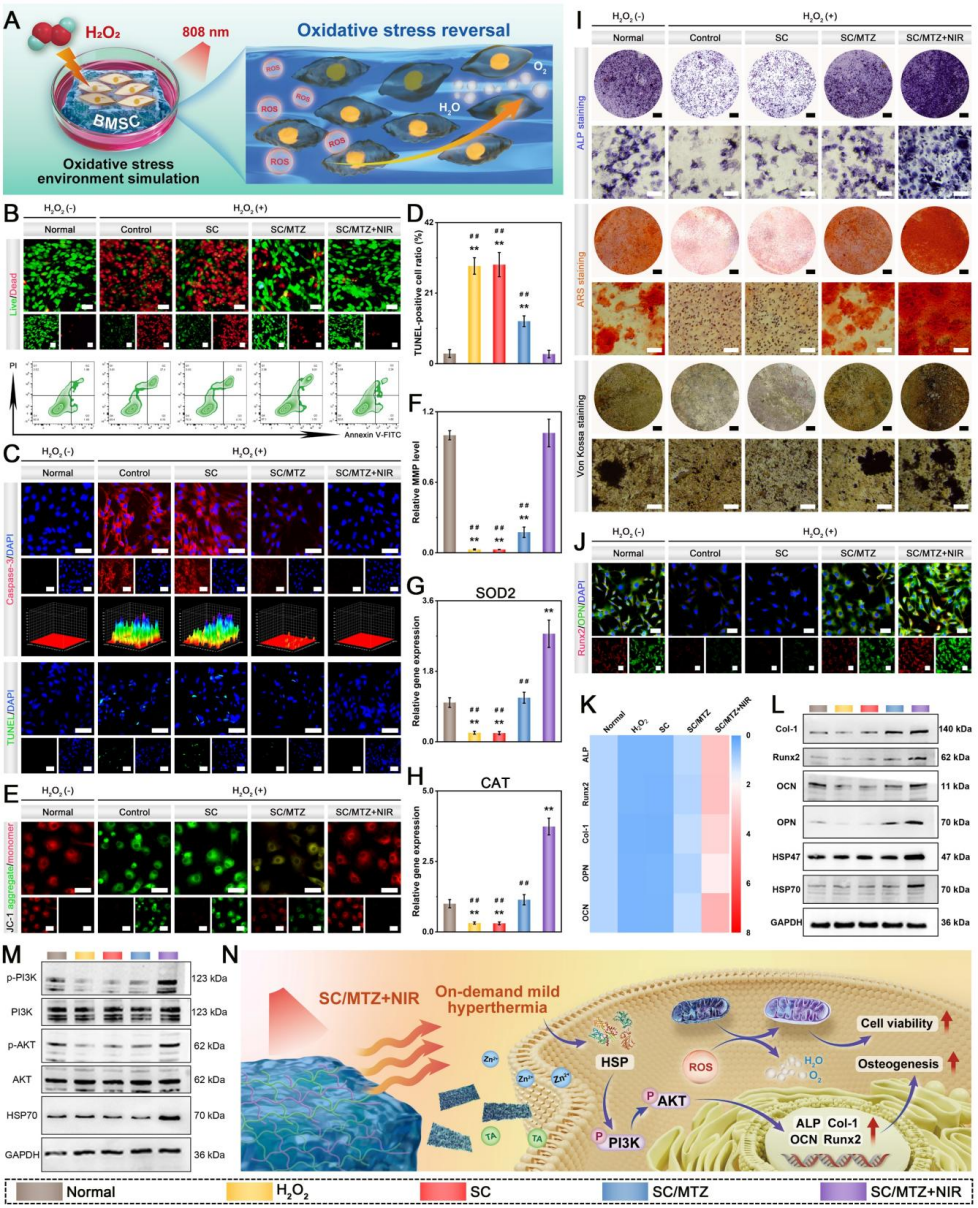
***In vitro* osteogenic ability under oxidative stress. (A)** Schematic illustration of ROS accumulation and osteogenic inhibition in BMSCs under oxidative stress. **(B)** Live/dead cell staining and flow cytometry analysis of the BMSCs in each group. Scale bar: 50 μm. **(C-D)** Representative images and quantitative analysis of caspase-3 and TUNEL staining in BMSCs following different treatments. Scale bar: 50 μm. **(E-F)** Representative images and quantitative analysis of JC-1 staining in BMSCs following different treatments. Scale bar: 50 μm. **(G-H)** Relative mRNA expression of antioxidant-related genes in BMSCs following different treatments. **(I)** Representative images of ARS, ALP, and Von Kossa staining of BMSCs following different treatments. Scale bar: 2 mm (macroscopic photos) and 100 μm (microscopic images). **(J)** Immunofluorescence staining of Runx2 and OPN in BMSCs following different treatments. Scale bar: 50 μm. **(K)** Heatmap of osteogenesis-related marker gene expression. **(L)** Western blot analysis was conducted to assess the expression of osteogenesis-related proteins in BMSCs following a 7-day coculture period. **(M)** Western blot analysis of PI3K/AKT signaling pathway-related proteins in BMSCs after different treatments for 7 days.** (N)** Schematic diagram of the mechanism by which SC/MTZ, combined with mild NIR irradiation, enhances osteoblast differentiation and mineralization. Data are expressed as mean ± SD (n = 3). Significant differences compared to the normal group are indicated by *P < 0.05 and **P < 0.01. Significant differences compared to the SC/MTZ+NIR group are indicated by ^#^P < 0.05 and ^# #^P < 0.01.

Previous research has suggested that excessive ROS production creates a detrimental microenvironment for bone regeneration by inducing oxidative stress and inflammation, which hinders BMSC survival and differentiation, accelerates apoptosis, and inhibits osteogenesis 14. Restoring redox balance and achieving effective antioxidation are essential for enhancing bone tissue repair under diabetic oxidative stress. After determining the cytocompatibility and antioxidant properties of the hydrogels, we investigated their protective effects on BMSCs in an H_2_O_2_-stimulated oxidative stress microenvironment **(Figure 7A)**. The results of the CCK-8 assay, live/dead staining, and flow cytometry analysis demonstrated that H_2_O_2_ stimulation significantly impaired cell function and induced cell death, resulting in reduced viability and increased apoptosis **(Figure 7B and Figure S14A)**. These effects were effectively mitigated by treatment with MTZ-incorporated hydrogels. The SC hydrogel minimally impacted H_2_O_2_-induced apoptosis in BMSCs. Compared with the SC/MTZ+NIR group, the SC/MTZ+NIR group presented greater cell viability, achieving levels comparable to those of cells not exposed to H_2_O_2_. The NIR-assisted SC/MTZ hydrogel system demonstrated significant *in vitro* cell protection in a ROS-induced environment, owing to its superior ROS-scavenging ability. An apoptosis assay confirmed that the SC/MTZ hydrogel combined with mild NIR irradiation protected cells from oxidative stress-induced apoptosis, reducing the apoptosis rate to levels similar to those observed in PBS-treated cells. TUNEL staining **(Figure 7C-D)** similarly demonstrated that both the SC/MTZ and SC/MTZ+NIR groups effectively reduced the degree of apoptosis caused by oxidative stress. The SC/MTZ+NIR group exhibited the fewest apoptotic cells, highlighting the combined anti-apoptotic effects of mild heat and MTZ nanosheets. Based on the aforementioned data, the photoactivated SC/MTZ hydrogel, with its outstanding antioxidant properties, can shield BMSCs from oxidative stress-induced damage by removing intracellular ROS, suggesting its great potential to enhance osteoblast proliferation and differentiation in an inflammatory setting.

Recent studies emphasize the importance of redox balance in bone regeneration, as dysregulation of oxidative stress and excessive ROS production can lead to lipid peroxidation, alter mitochondrial membrane potential (MMP), and cause mitochondrial dysfunction, thereby impeding bone healing 81. Therefore, not only should ROS scavenging and antioxidant capacity be evaluated, but mitochondrial function should also be investigated for bone defect repair under diabetic conditions. Given that mitochondria are the primary producers of ROS in cells, a JC-1 assay was performed to assess MMP in BMSCs following various treatments. **Figure 7E-F** illustrates that both the H_2_O_2_-stimulated and SC hydrogel groups exhibited significantly elevated levels of JC-1 monomers (green fluorescence) compared to those in the blank control group. In contrast, BMSCs treated with MTZ-incorporated composite hydrogels showed increased JC-1 aggregate fluorescence (red) and decreased JC-1 monomer fluorescence. The increase in caspase-3 expression, a key protease activated during early apoptosis, in the control group following H_2_O_2_ treatment **(Figure 7C)** corroborated the flow cytometry findings. Compared with the control group, the SC/MTZ group exhibited increased MMP levels and reduced caspase-3 activity. The application of gentle NIR irradiation enhanced the effect, with the SC/MTZ+NIR group showing the highest MMP and lowest caspase-3 activity. This is likely due to the synergistic ROS-scavenging effect of mild photothermal stimulation, which aids cellular functional recovery. This study indicated that the synergistic regulation of ROS and mild photothermal effects in the SC/MTZ+NIR group significantly improved mitochondrial function in BMSCs under oxidative stress, elucidating the role of the photoactivated hydrogel system in enhancing BMSC anti-apoptotic effects. We conducted qRT-PCR to examine the expression levels of antioxidant genes in BMSCs and to clarify the underlying antioxidant mechanism. **Figure 7G-H** illustrates that the SOD2 and CAT expression levels in the BMSCs were notably suppressed in both the H_2_O_2_-treated and SC groups. Treatment with the SC/MTZ hydrogels notably counteracted the inhibitory effects, with the SC/MTZ+NIR group showing the highest levels of antioxidant gene expression. These results suggest that the SC/MTZ hydrogel, combined with mild NIR irradiation, may enhance oxidative stress resistance in BMSCs by upregulating antioxidant gene expression. The photoactivated SC/MTZ hydrogel created a supportive microenvironment for BMSCs by disrupting the cycle of ROS overproduction, mitochondrial dysfunction, and the degradation of antioxidant defenses.

Given the exceptional ability of photoactivated SC/MTZ hydrogels to eliminate intracellular ROS and restore mitochondrial function, we evaluated the *in vitro* osteogenic differentiation and mineralization of BMSCs under H_2_O_2_-induced oxidative stress. **Figure 7I** shows that H_2_O_2_ exposure markedly diminished the osteogenic capacity of BMSCs compared with the negative control group, leading to reduced ALP activity and ECM mineralization. Elevated oxidative stress may impair osteogenic function by causing permanent cellular damage through the oxidation of nucleic acids and proteins 82. SC/MTZ hydrogel treatment combined with NIR-triggered mild thermal stimulation mitigated adverse effects and enhanced osteogenesis, outperforming the control group not exposed to H_2_O_2_. Compared with the H_2_O_2_ group, both the SC/MTZ and SC/MTZ+NIR groups presented substantially greater ALP expression levels and ECM mineralization, with the SC/MTZ+NIR group showing a more pronounced effect than the SC/MTZ group **(Figure S14B-C)**. These findings highlight the significant osteogenic potential of injectable hydrogels that combine mild hyperthermia and ROS elimination, effectively improving impaired osteogenesis and biomineralization of BMSCs under oxidative stress. Runx2, a key transcription factor in osteoblast differentiation, is essential for bone formation. OPN is a phosphorylated glycoprotein that plays a role in organizing and depositing the bone matrix. Runx2 serves as an early marker of osteogenic differentiation, whereas OPN is typically secreted during the middle to late stages of this process 83. **Figure 7J** illustrates that immunofluorescence staining showed enhanced fluorescence intensity of Runx2 and OPN in the SC/MTZ and SC/MTZ+NIR groups, while significant reductions were noted in the H_2_O_2_ and SC groups. Compared with those in the SC/MTZ+NIR group, the Runx2 and OPN expression levels in the SC/MTZ+NIR group were significantly greater** (Figure S15)**. A similar trend was observed in the osteogenic potential of pre-osteoblastic MC3T3-E1 cells **(Figure S16)**, indicating that the SC/MTZ hydrogel mitigated impaired osteogenesis and enhanced osteogenesis under gentle NIR irradiation. To explore the osteogenic potential of BMSCs under oxidative stress, qRT-PCR and western blot analyses were performed, as shown in **Figure 7K-L**. Runx2 expression was significantly increased in the SC/MTZ hydrogel-treated group, and the levels of the early osteogenic genes ALP and Col-1, as well as the late osteogenic markers OPN and OCN, increased. Notably, the SC/MTZ+NIR group showed a significant increase in osteogenic gene expression compared with the SC/MTZ and control groups. Western blot and qRT-PCR analyses demonstrated that H_2_O_2_ addition significantly reduced Runx2, Col-1, OPN, and OCN levels, while treatment with the SC/MTZ hydrogel combined with mild hyperthermia increased their expression **(Figure 7L and Figure S17)**. Numerous studies have shown that Zn^2+^, TA, and MXene nanosheets significantly promote osteogenic differentiation and mineralization, particularly under pathological inflammatory conditions 8, 51, 73. The application of mild photothermal stimulation could significantly increase ALP activity, accelerate calcium deposition, and upregulate the expression of osteogenesis-related markers and HSPs 84. These results demonstrate that combining on-demand mild hyperthermia with ROS scavenging significantly accelerates osteogenic differentiation at both early and late stages in an oxidative stress microenvironment.

To further investigate the significant role of SC/MTZ+NIR treatment in inducing osteogenesis under oxidative stress, the expression of the shock proteins HSP47 and HSP70 was evaluated. HSP70, an essential HSP family protein, enhances BMSC heat resistance, boosts ALP activity, and promotes bone mineralization 85. Western blot analysis revealed that the expression of the heat shock proteins HSP47 and HSP70, which are associated with osteogenesis, was elevated exclusively in the SC/MTZ+NIR treatment group **(Figure 7L)**, with no notable differences found between the control and other experimental groups **(Figure S17)**. This result further supports our previous finding that mild hyperthermia can modulate HSP expression and osteogenic differentiation of BMSCs *in vitro* 19. The expression of HSP70 and the phosphorylation of AKT enhance osteoblast MMP activity and inhibit caspase-3 activation, thereby reducing H_2_O_2_-induced apoptosis, mitigating mitochondrial dysfunction, and disrupting the cycle of excessive ROS and inflammation in the diabetic microenvironment 22, 55. Western blot analysis was conducted to confirm the principal proteins in the PI3K/AKT signaling pathway, as shown in **Figure 7M**. The SC/MTZ+NIR group showed increased HSP70 and p-AKT, along with decreased AKT, indicating that mild heat combined with MTZ loading synergistically activated the PI3K/AKT signaling pathway **(Figure S18)**, thereby contributing to osteogenic differentiation and bone metabolism. In summary, the SC/MTZ hydrogel system, designed for on-demand photothermal therapy, exhibited exceptional *in vitro* biological properties. The biomimetic ECM-like microstructure offers excellent biocompatibility, providing structural support for osteogenesis-related cell adhesion and facilitating signal transduction to regulate cell functions, including proliferation, migration, differentiation, and ECM deposition. Conversely, NIR-mediated mild PTT with ROS scavenging can improve the impaired regenerative microenvironment by boosting antioxidant activity, restoring mitochondrial balance, and facilitating osteoblast differentiation and mineralization under oxidative stress. HSP70, known for its crucial role in enhancing bone repair and osteogenic differentiation, was significantly upregulated after SC/MTZ+NIR treatment. This upregulation promotes the osteogenic differentiation and mineralization of BMSCs through the PI3K/AKT signaling pathway. **Figure 7N** illustrates the molecular mechanism by which the photoactivated SC/MTZ hydrogel system enhances the osteogenic differentiation of BMSCs under oxidative stress.

### *In vitro* anti-inflammatory and immunomodulatory effects

**Figure 8:**
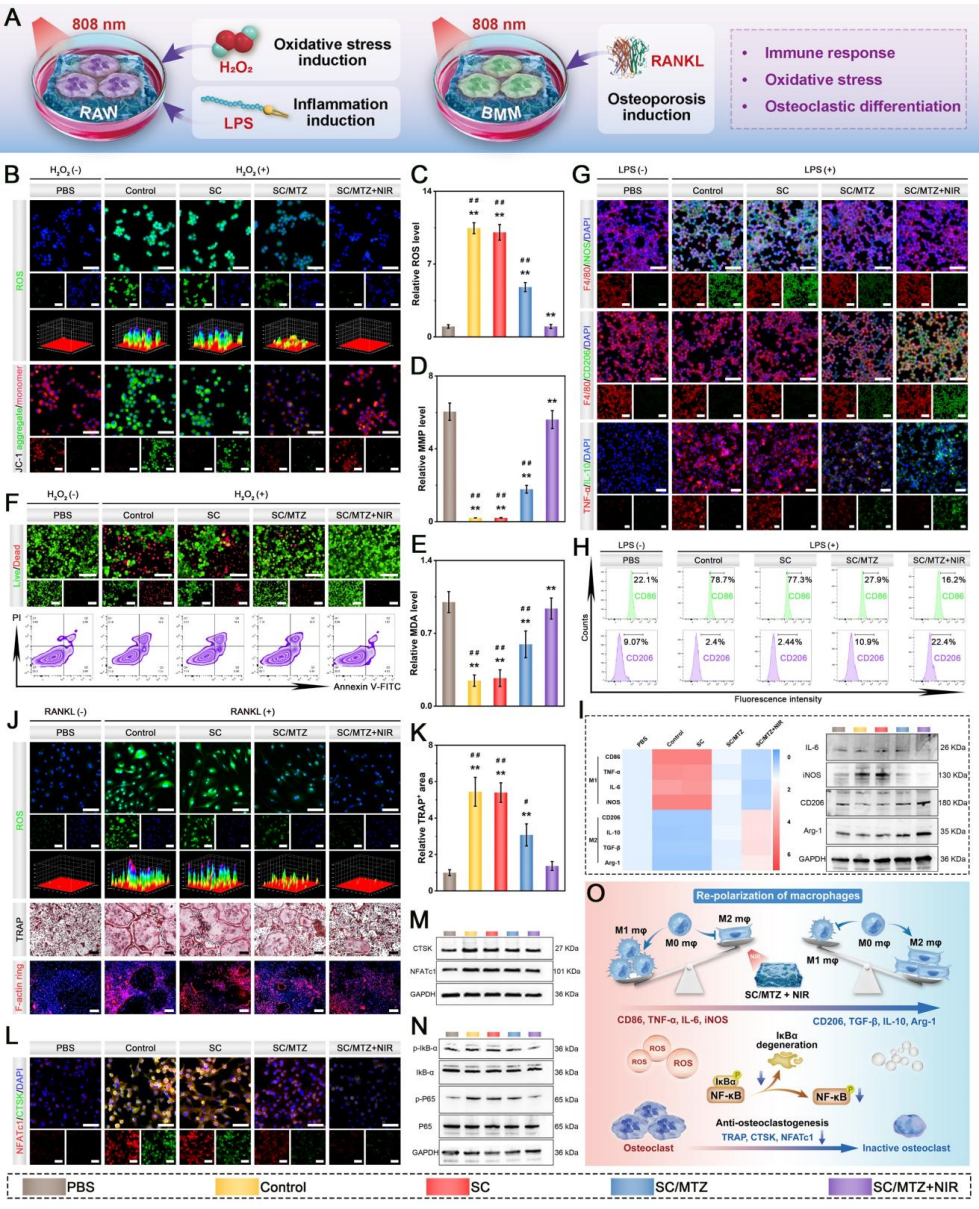
***In vitro* ROS-scavenging, immunomodulatory, and anti-osteoclastic abilities. (A)** Diagram depicting how SC/MTZ modulates macrophages and BMMs when exposed to gentle NIR irradiation. **(B-D)** Representative images and quantitative analysis of DCFH-DA and JC-1 staining in macrophages following different treatments. Scale bar: 50 μm. **(E)** Quantitative analysis of the MDA content in macrophages after various treatments.** (F)** Live/dead cell staining and flow cytometry analysis of macrophages in each group. Scale bar: 100 μm. **(G)** Immunofluorescence staining was conducted on macrophages to assess the expression of F4/80, iNOS, CD206, TNF-α, and IL-10 following different treatments. Scale bar: 50 μm. **(H)** Flow cytometry was used to analyze CD86 and CD206 expression levels in macrophages after various treatments.** (I)** Heatmap and western blot analyses were conducted to assess the expression of inflammation-related markers in macrophages subjected to various treatments. **(J)** Representative images of DCFH-DA, TRAP, and F-actin ring formation assays in BMMs post-treatment. Scale bar: 50 μm (DCFH-DA staining) and 200 μm (TRAP staining and F-actin ring formation assay). **(K)** Quantitative analysis of TRAP staining in each group. **(L)** Immunofluorescence staining of NFATc1 and CTSK in BMMs after various treatments. Scale bar: 50 μm. **(M-N)** Western blot analysis of osteoclastogenesis- and NF-κB pathway-related protein expression in BMMs after various treatments. **(O)** Schematic representation of how SC/MTZ+NIR influences macrophage polarization and osteoclast activity to promote bone regeneration. Data are expressed as mean ± SD (n = 3). Significant differences compared to the PBS group are indicated by *P < 0.05 and **P < 0.01. Significant differences compared to the SC/MTZ+NIR group are indicated by ^#^P < 0.05 and ^# #^P < 0.01.

After bone injury, the timely conversion of macrophages from a proinflammatory M1 phenotype to an anti-inflammatory M2 phenotype is critical for resolving inflammation and facilitating tissue regeneration 86. Proinflammatory M1 macrophages contribute to chronic inflammation and bone destruction by elevating the secretion of inflammatory cytokines, including iNOS, IL-6, and TNF-α. M2 macrophages, known for their anti-inflammatory and pro-regenerative roles, aid in tissue remodeling and repair primarily by secreting beneficial cytokines such as IL-10, TGF-β, VEGF, and BMP-2 70. Unfortunately, among individuals with diabetes, especially when infections co-occur, a heightened inflammatory response results in elevated ROS levels, which cause oxidative damage to cells and mitochondrial dysfunction, further hindering macrophage phenotype transition and maintaining tissues in a prolonged inflammatory state 87. Effective healing of diabetic bone defects requires anti-inflammatory strategies to transition from the inflammatory phase to the proliferative and reparative phases. These strategies should focus on reducing excess ROS with antioxidants, restoring mitochondrial function, and promoting M2 macrophage polarization. To examine how hydrogels regulate inflammation, macrophages were exposed to LPS, a bacterial endotoxin, to trigger inflammation and increase ROS production **(Figure 8A)**. The results of DCFH-DA staining showed intense green fluorescence in both the control and SC groups **(Figure 8B)**, indicating significant cellular oxidative stress induced by LPS. Conversely, the SC/MTZ group showed minimal ROS-related fluorescence, which disappeared following mild photothermal treatment. Compared with the SC/MTZ hydrogel-treated group, the SC/MTZ+NIR group demonstrated superior ROS-scavenging capabilities. This improvement is attributed to enhanced MTZ decomposition, facilitated by efficient, mild thermal stimulation, resulting in increased ion and TA release rates. Compared with those in the H_2_O_2_-treated positive control and SC groups, the intracellular ROS levels in the SC/MTZ+NIR group were significantly lower, with a fluorescence intensity similar to that in the PBS group **(Figure 8C)**. The malondialdehyde (MDA) assay results indicated that SC/MTZ+NIR significantly mitigated the LPS-induced rise in MDA levels, highlighting its efficacy in reducing intracellular ROS **(Figure 8E)**. JC-1 and live/dead cell staining indicated that SC/MTZ hydrogel treatment stabilized MMP and reduced LPS-induced endogenous ROS, confirming its efficacy in scavenging and protecting against intracellular ROS **(Figure 8B, 8D, 8F)**. Flow cytometry and CCK-8 assay results **(Figure 8F and Figure S19A)** confirmed that the SC/MTZ hydrogel effectively reduced apoptosis and enhanced macrophage viability under oxidative stress. In conclusion, this study's results indicate that the photoactivated SC/MTZ hydrogel system effectively protects cells from radical damage and improves mitochondrial function, demonstrating its promise for diabetic tissue regeneration. Moreover, the appreciable ROS-scavenging/antioxidant properties of SC/MTZ+NIR support immune regulation, aiding in the reversal of macrophage inflammation and promoting regeneration.

Considering the crucial impact of macrophage polarization on immunomodulation and bone regeneration, we examined how hydrogels influence macrophage phenotype transition via immunofluorescence staining. **Figure 8G** illustrates that, compared with the LPS, PBS, and SC groups, the SC/MTZ group exhibited notably reduced expression of iNOS, an M1 macrophage marker, and elevated expression of CD206, an M2 macrophage marker. The SC/MTZ hydrogel successfully promoted macrophage polarization toward the M2 phenotype under LPS-induced inflammation, which was attributed to the significant antioxidant and anti-inflammatory properties of MTZ heterojunctions. Notably, the highest CD206 fluorescence intensity observed in the SC/MTZ+NIR group was attributed mainly to the synergistic amplification of the immunomodulatory capacity induced by MTZ heterojunctions and mild heat stimulation. TNF-α is a crucial mediator of inflammation, while IL-10 serves as a primary regulator of immune suppression. Modulating these inflammation-related cytokines reflects the anti-inflammatory potential of the hydrogels 88. **Figure 8G** demonstrates that SC/MTZ hydrogel treatment significantly decreased TNF-α levels while elevating IL-10 levels in LPS-induced macrophages. This anti-inflammatory effect was further enhanced upon exposure to gentle NIR irradiation. These results suggest that SC/MTZ+NIR induced M1-to-M2 macrophage polarization and alleviated inflammation, potentially benefiting bone defect repair. The effects of the hydrogels on macrophage polarization were subsequently explored using flow cytometry. **Figure 8H** illustrates that the SC/MTZ+NIR group exhibited the lowest ratio of CD86^+^ cells (M1 macrophages). In contrast, the control and SC/MTZ groups showed higher ratios, and the LPS group displayed the highest. An inverse trend was observed for CD206^+^ cells (M2 macrophages), with the highest ratio in the SC/MTZ+NIR group. Compared with SC treatment, SC/MTZ+NIR treatment markedly increased the expression of anti-inflammatory factors (IL-10 and IL-4) while significantly reducing the expression of proinflammatory factors (TNF-α and IL-6) **(Figure S20)**. These findings validate the ability of SC/MTZ+NIR to inhibit LPS-induced M1 polarization, promote M2 polarization, and regulate cytokine secretion in response to inflammation. We expect that applying the SC/MTZ hydrogel with mild NIR irradiation to bone defect areas will create an anti-inflammatory microenvironment conducive to osteogenesis. This is facilitated by the SC/MTZ hydrogel's ability to deliver TA and Zn^2+^ in a targeted manner under gentle NIR irradiation, which possesses anti-inflammatory properties. Additionally, prior research has indicated that implants with porous structures and hydrophilic surfaces can promote an anti-inflammatory response in immune cells 46. These findings underscore the *in vitro* immunomodulatory capacity of SC/MTZ+NIR, which is essential for tissue repair and regeneration. **Figure 8I** illustrates that both qRT-PCR and immunofluorescence staining revealed elevated expression of proinflammatory markers (TNF-α, IL-6, iNOS, and IL-β) in the LPS group, which was significantly reduced by SC/MTZ hydrogel treatment combined with gentle NIR irradiation. SC/MTZ hydrogel treatment increased the levels of the anti-inflammatory markers IL-10, IL-4, CD206, and Arg-1, and mild PTT enhanced this effect. The western blot analysis results were consistent with the trends observed in the immunofluorescence and qRT-PCR results **(Figure 8I and Figure S21)**. These findings indicate that the combination of the SC/MTZ hydrogel and mild hyperthermia synergistically reprograms macrophages from the M1 phenotype to the M2 phenotype.

### *In vitro* osteoclast inhibition effects

Compared with normal bone tissue, diabetic bone defect sites exhibit higher levels of ROS, which hinder osteogenic differentiation and promote osteoclast maturation, thereby disrupting bone homeostasis and leading to significant bone resorption 15. Excessive ROS generation activates NF-κB and NFATc1, promoting their nuclear translocation and the expression of osteoclast-related genes, thereby enhancing bone resorption 10. In this work, an *in vitro* RANKL-induced osteoclast differentiation model was established that simulates the inflammatory bone environment. Before conducting osteoclast differentiation experiments, we assessed the effect of the composite hydrogel on intracellular ROS levels in BMMs using DCFH-DA staining. The intracellular ROS levels in the BMMs significantly increased following RANKL stimulation, as indicated by the increase in the intensity of the green fluorescence signals **(Figure 8J)**. Following SC/MTZ hydrogel treatment, the ROS fluorescence intensity decreased significantly, closely matching that of the control group, particularly under mild heat stimulation. To effectively mitigate the adverse effects of oxidative stress-induced osteoclast hyperactivation on bone healing, the utilization of antioxidants has become an important therapeutic strategy for inflammation-related bone diseases. Recent research has shown that biomaterial scaffolds with excellent antioxidant activity can inhibit ROS overproduction induced by RANKL in macrophages, fostering macrophage reprogramming to the M2 phenotype and markedly hindering their differentiation into osteoclasts 89. An increasing body of research suggests that proinflammatory M1 macrophages may aggravate inflammation and facilitate osteoclast formation, whereas M2 macrophages may secrete various anti-inflammatory factors to inhibit the inflammatory response and promote osteo-/angiogenesis 90. To further investigate the potential impacts of SC/MTZ+NIR on osteoblast differentiation, the expression of osteoclast-specific genes was examined via qRT-PCR during osteoclastogenesis following RANKL treatment. Our results demonstrated significant downregulation of osteoclastogenesis-related genes (CTSK, MMP9, NFATc1, and c-Fos) following SC/MTZ hydrogel treatment **(Figure S22)**. These results align with earlier studies indicating that removing ROS hinders osteoclast formation triggered by RANKL signaling 91, as confirmed by immunofluorescence staining and western blot analysis **(Figure 8L-M)**. Upon RANKL stimulation, the expression of NFATc1 and CTSK was significantly increased, whereas the stimulatory effects of RANKL were suppressed by SC/MTZ hydrogel treatment. The inhibitory effect of SC/MTZ on osteoclastogenesis was particularly pronounced following gentle NIR laser irradiation, underscoring the synergistic regulatory role of mild thermal stimulation in preventing osteoclastogenesis. The downregulation of osteoclastogenesis-related marker expression observed with SC/MTZ+NIR treatment *in vitro* might be partially attributed to the inhibition of ROS generation. Besides, NIR-triggered release of Zn^2+^ and TA has been shown to suppress osteoclastogenesis by effectively interfering with RANKL-induced signaling pathways 21.

We performed TRAP staining and F-actin ring formation assays to assess the effects of the composite hydrogels on BMM osteoclastic differentiation **(Figure 8J)**. TRAP, an indicator of the osteoclast phenotype, is present in osteoclast cytoplasm and is essential for osteoclast differentiation. RANKL stimulates mononuclear macrophages to proliferate and differentiate, leading to their fusion into multinucleated osteoclasts, which are key agents of bone resorption in osteoporosis. Following 6 days of osteoclastic induction, the SC/MTZ+NIR group exhibited a marked decrease in the TRAP^+^ cell area and F-actin ring formation **(Figure 8K and Figure S19B)**, indicating significantly suppressed osteoclast formation and activity. Given the elevated ROS levels in BMMs following RANKL treatment and the critical role of the NF-κB pathway in osteoclast differentiation, this study further investigated how SC/MTZ+NIR inhibits BMM osteoclastogenesis. The NF-κB signaling pathway acts as a central hub that orchestrates both inflammatory responses and osteoclastogenesis. Excessive activation of NF-κB drives the expression of proinflammatory mediators and key osteoclastogenic transcription factors, thereby exacerbating inflammation and promoting abnormal osteoclast differentiation, both of which severely disrupt bone remodeling and impair diabetic bone repair 92. Protein expression associated with the NF-κB signaling pathway was characterized via western blot analysis. **Figure 8M-N** illustrates that SC/MTZ+NIR treatment significantly reduced the expression of p-IkB-α and p-P65, while the P65 and IkB-α levels remained unchanged. This treatment also notably decreased CTSK and NFATc1 protein levels **(Figure S23)**. The SC/MTZ hydrogel appears to regulate bone regeneration bidirectionally by promoting M2 macrophage polarization and suppressing osteoclastogenesis, which is achieved through NF-κB pathway inhibition and ROS scavenging **(Figure 8O)**. This dual functionality underscores its promise as a therapeutic approach for bone regeneration in diabetic conditions linked to redox imbalance.

### *In vitro* macrophage reprogramming-mediated osteogenic behavior

**Figure 9:**
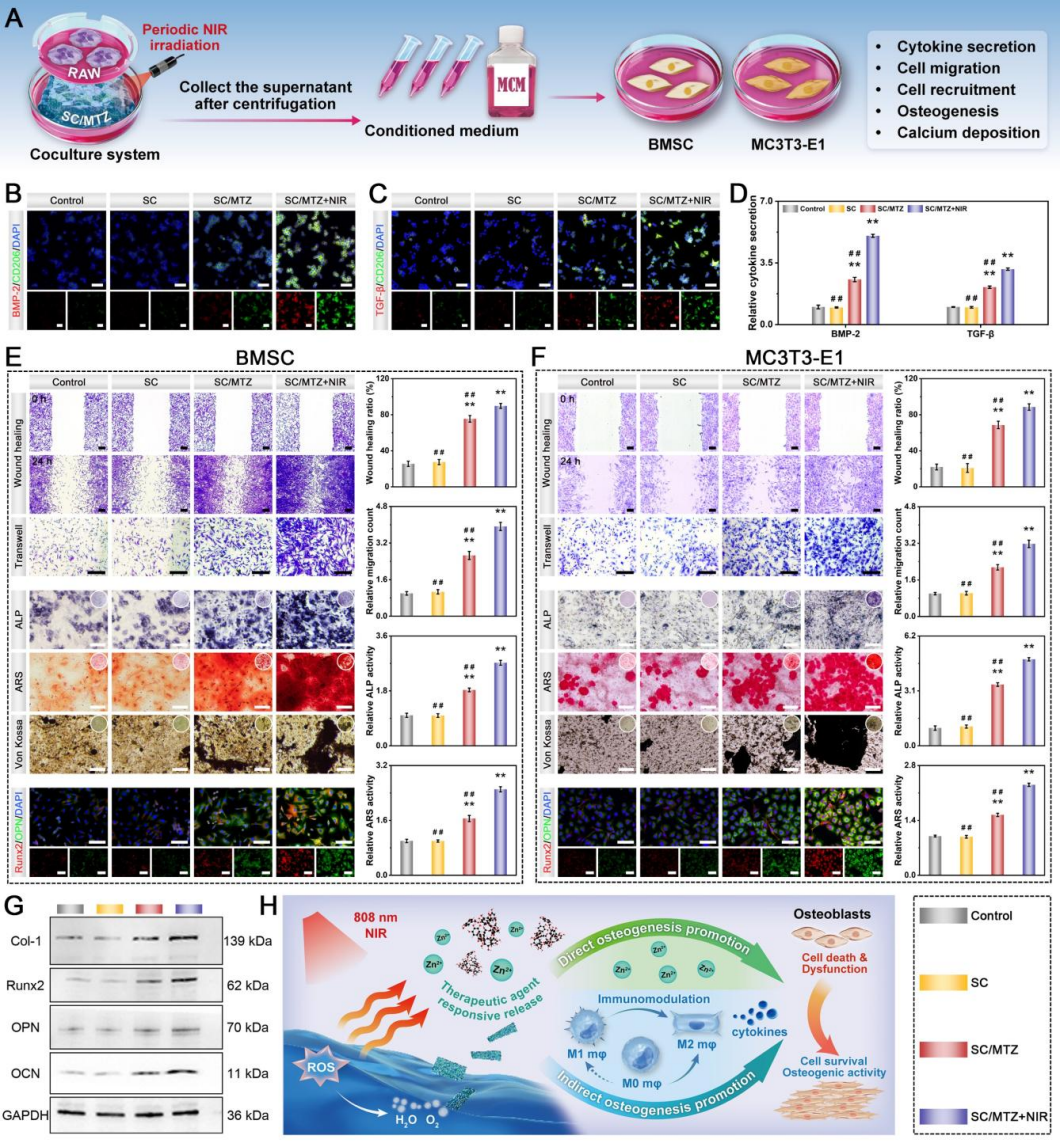
***In vitro* immuno-modulated osteogenesis. (A)** Diagram illustrating the cell culture process using conditioned medium. **(B-C)** Immunofluorescence staining was conducted to analyze CD206, BMP-2, and TGF-β expression in macrophages following different treatments. Scale bar: 50 μm. **(D)** ELISA analysis of macrophage-secreted BMP-2 and TGF-β following different treatments. **(E)** Representative images and quantitative analyses of wound healing, Transwell migration, ALP, ARS, and Von Kossa staining, and immunofluorescence staining for Runx2 and OPN in BMSCs post-treatment. Scale bar: 50 μm (Transwell migration assay) and 100 μm (wound healing assay, ALP staining, ARS staining, Von Kossa staining, and immunofluorescence staining).** (F)** Representative images and quantitative analyses of wound healing, Transwell migration, ALP, ARS, and Von Kossa staining, and immunofluorescence staining for Runx2 and OPN in MC3T3-E1 cells post-treatment. Scale bar: 50 μm (Transwell migration assay) and 100 μm (wound healing assay, ALP staining, ARS staining, Von Kossa staining, and immunofluorescence staining). **(G)** Analysis of osteogenic marker expression in BMSCs post-treatment was conducted using Western blot and qRT-PCR. **(H)** Schematic depiction of the endogenous bone regeneration process, where initial immunomodulation triggers the recruitment and differentiation of BMSCs. Data are expressed as mean ± SD (n = 3). Significant differences compared to the control group are indicated by *P < 0.05 and **P < 0.01. Significant differences compared to the SC/MTZ+NIR group are indicated by ^#^P < 0.05 and ^# #^P < 0.01.

Recent research indicates that prompt, efficient macrophage reprogramming from the M0/M1 to the M2 phenotype mitigates acute inflammation by releasing anti-inflammatory mediators and beneficial cytokines. This process creates a supportive osteoimmune microenvironment conducive to the migration, recruitment, and differentiation of osteogenesis-related cells, such as BMSCs and osteoblasts 64. These *in vitro* findings consistently indicate that the macrophage-modified immune microenvironment can trigger osteogenesis by upregulating the expression of pro-regenerative growth factors and cytokines. Therefore, to validate whether SC/MTZ+NIR-mediated M2 macrophage activation can exert osteoimmunomodulatory effects on BMSCs and MC3T3-E1 cells, we explored the crosstalk among these three types of bone healing-related cells via conditioned medium experiments, as shown in **Figure 9A**. Numerous studies have indicated that M2 macrophage reprogramming significantly reduces inflammation and facilitates bone regeneration by enhancing the secretion of osteogenesis-related factors, thereby linking initial inflammation with subsequent tissue regeneration and repair 93. In this study, BMP-2 and TGF-β were selected as representative osteogenic cytokines to assess M2 macrophage activation by the hydrogels **(Figure 9B-C)**. BMP-2 and TGF-β are key regulators of osteogenesis, with BMP-2 playing a particularly significant role in promoting new bone ingrowth and stimulating bone formation 79. Immunofluorescence staining indicated a substantial presence of CD206^+^ macrophages expressing BMP-2 and TGF-β in the SC/MTZ group, with further enhancement in the SC/MTZ+NIR group. ELISA confirmed the presence of growth factors in the conditioned medium** (Figure 9D)**. The SC/MTZ hydrogel-treated samples exhibited elevated BMP-2 and TGF-β levels, which further increased with gentle NIR irradiation, potentially enhancing the osteo-supportive microenvironment for *in situ* bone defect healing. Additionally, the supernatant concentrations of TA and Zn^2+^ were assayed via UV-vis spectrophotometry and ICP-OES, respectively. **Figure S24** illustrates that the supernatant levels of TA and Zn^2+^ in the SC/MTZ group are lower than those in the SC/MTZ+NIR group, likely due to the NIR-responsive release behavior of the composite system. These findings align with prior results 33, suggesting that changes in macrophage type influence bone regeneration by increasing levels of pro-regenerative growth factors and cytokines, such as TGF-β and BMP-2, thus offering a robust theoretical basis for improving bone repair. Functional cytokines play important roles in chemotaxis, guiding osteogenesis-related cells to injury sites, which are essential for effective bone repair.

Functional cytokines play important roles in chemotaxis, guiding osteogenesis-related cells to injury sites, which are essential for effective bone repair 83. The study assessed the impact of different conditioned media on the migration of BMSCs and osteoblasts. **Figure 9E-F** shows that, compared with the SC and control groups, the SC/MTZ group demonstrated increased migration and recruitment of BMSCs and osteoblasts. This effect is likely due to the release of anti-inflammatory cytokines (IL-10 and IL-4) and growth factors (TGF-β and BMP-2), highlighting the potential of SC/MTZ to facilitate bone regeneration. The promotion effect is based on SC/MTZ-induced M2 macrophage activation, resulting in the generation of functional growth factors and chemokines, as documented in the literature 80. It is further amplified by mild NIR irradiation. In addition, the NIR-triggered intelligent release of Zn^2+^ ions from the SC/MTZ hydrogel also synergistically accelerates cell migration and recruitment. Studies indicate that the swift migration and recruitment of BMSCs and osteoblasts are essential for initiating osteogenesis and biomineralization in bone repair 83. These findings highlight the efficacy of SC/MTZ+NIR in enhancing cell migration and recruitment, which are essential for the accumulation of bone progenitor cells at the defect site and facilitating bone regeneration.

Macrophage cytokines not only facilitate the migration and recruitment of osteogenesis-related cells but also potentially enhance the osteogenic differentiation and mineralization of BMSCs, fostering a pro-regenerative microenvironment that supports bone healing 16. We subsequently investigated the osteogenic effects of M2 macrophage activation on BMSCs and MC3T3-E1 cells. **Figure 9E-F** demonstrates a notable increase in ALP activity in both the SC/MTZ and SC/MTZ+NIR groups, with the SC/MTZ+NIR group showing the most tremendous osteogenic potential. ARS staining demonstrated a significant enhancement in mineralized matrix formation in the SC/MTZ+NIR group. Compared with those in the other groups, the ALP and ARS activities in the SC/MTZ+NIR group were significantly greater, highlighting the crucial role of M2 macrophage activation and mild heat stimulation in enhancing osteogenic mineralization. The results of Von Kossa staining indicated a notable increase in mineralized nodule formation in the SC/MTZ+NIR group, followed by the SC/MTZ group, aligning with the trends observed via ALP staining. Moreover, immunofluorescence staining revealed that Runx2 and OPN levels in the SC/MTZ group were higher than those in the control and SC groups, with the highest expression observed in the SC/MTZ+NIR group. qRT-PCR and western blot analyses **(Figure 9G and Figure S25)** demonstrated that the osteogenic markers Col-1, Runx2, OPN, and OCN were expressed at significantly higher levels in the SC/MTZ and SC/MTZ+NIR groups than in the control and SC groups. Notably, the SC/MTZ+NIR group exhibited the highest expression levels **(Figure S26)**. The SC/MTZ+NIR group showed a marked increase in early- and late-stage osteogenic marker expression, indicating that the mild hyperthermia-assisted hydrogel combination fostered an optimal osteoimmune microenvironment, promoting improved osteogenic differentiation and mineralization of BMSCs and osteoblasts.

As reported in the literature, macrophages are instrumental in biomaterial-induced osteogenesis and orchestrate the complex interplay among various cell types, including osteoblasts, fibroblasts, and angioblasts, to facilitate bone healing 34. The established effects of Zn^2+^ and mild thermal stimulation on immune modulation involve both innate and adaptive responses. Specifically, zinc-enriched biomaterials are recognized for their regulatory effects on innate immunity, particularly by modulating macrophage activity. On the continuum of bone defect healing, the initial influx of proinflammatory M1 macrophages marks the onset of the healing cascade, which subsequently shifts toward a predominance of anti-inflammatory M2 macrophages as healing progresses 94. Zn-based materials are essential for this transition, fostering an environment conducive to osteogenesis and the comprehensive restoration of tissue integrity 95. This biological effect can be further amplified by mild thermal stimulation, which synergistically promotes M2 macrophage polarization. Our previous study demonstrated that combining Zn-based biomaterials with mild hyperthermia therapy promotes macrophage polarization toward a pro-healing M2 phenotype, thereby creating an immune microenvironment that accelerates endogenous bone regeneration 22. Catechol-rich TA enhances the antioxidant and anti-inflammatory properties of the SC/MTZ hydrogel by increasing the levels of IL-10, CD206, and IL-4, facilitating macrophage M2 polarization, reducing inflammation, and promoting the recruitment and osteogenic differentiation of endogenous stem cells. By utilizing the M2-dominant immune microenvironment, the SC/MTZ+NIR group enhanced the migration and osteogenic differentiation of MC3T3-E1 cells and BMSCs by releasing pro-regenerative growth factors and cytokines, such as TGF-β and BMP2, thereby promoting angiogenesis and bone regeneration. As reported in the literature, the intrinsic characteristics of biomaterials, including their surface morphology, hydrophobicity, and chemical composition, are crucial for directing bone repair processes 96. These properties influence the local immune microenvironment, highlighting that SC/MTZ+NIR orchestrates a well-timed shift in the inflammatory/immune response. This shift significantly impacts stem cell behavior and osteogenic differentiation, promoting active involvement in tissue regeneration** (Figure 9H)**.

### *In vivo* evaluation of the foreign-body reaction and biological performance of the hydrogels

**Figure 10:**
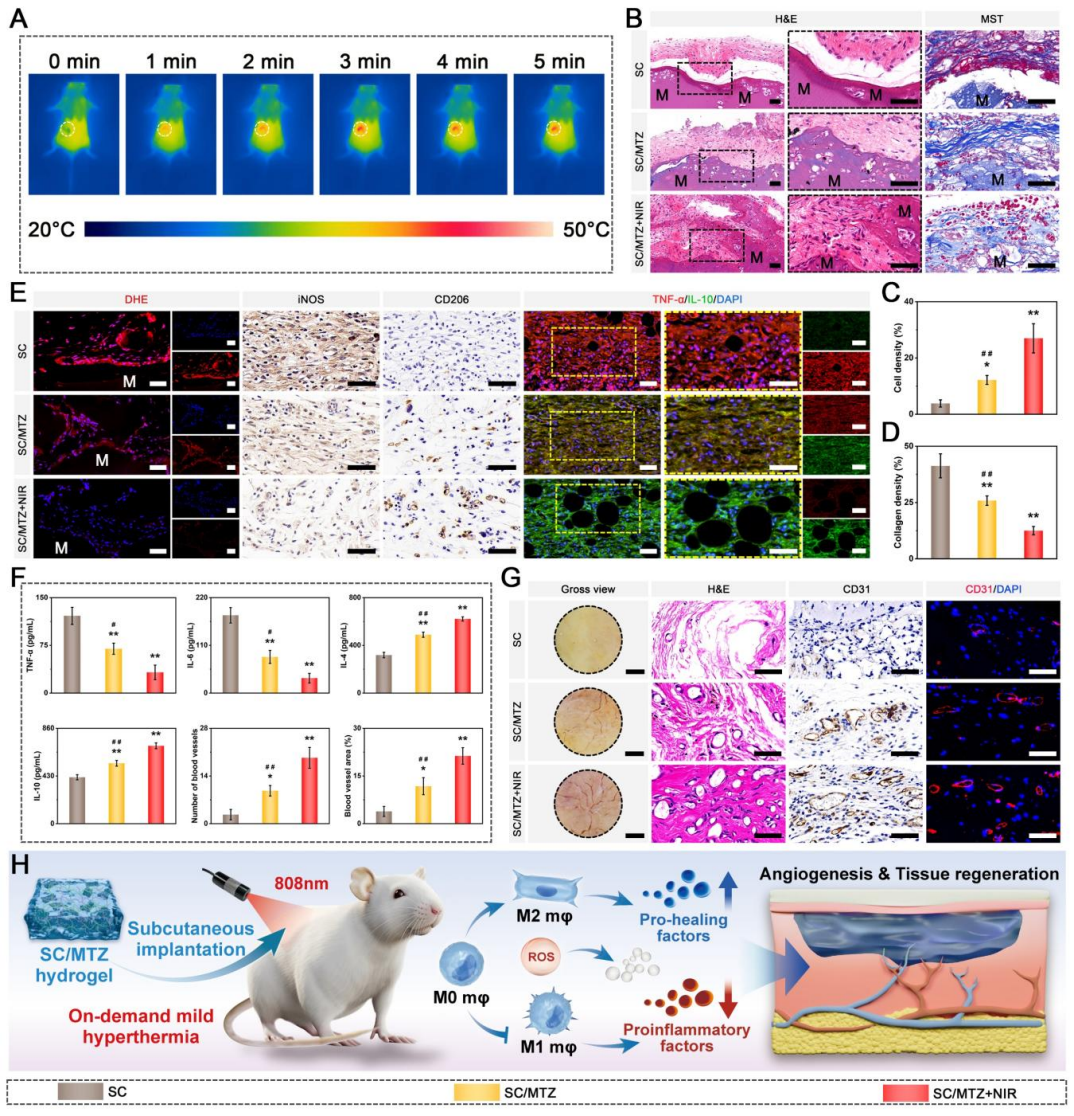
***In vivo* immune response and neovascularization. (A)** Representative photographs and thermal infrared images depict the dorsal skin of BALB/c mice treated with SC/MTZ and irradiated with an 808 nm laser (0.5 W/cm², 5 min). **(B)** H&E and MST staining of the hydrogel and adjacent tissue at 14 days post-implantation. Scale bar: 50 μm. **(C-D)** Quantitative analysis of the cell density and collagen density inside the hydrogels after implantation. **(E)** Representative images of DHE, iNOS, and CD206 immunohistochemical staining, along with TNF-α and IL-10 immunofluorescence staining, at 14 days post-implantation. Scale bar: 50 μm.** (F)** Quantification of inflammatory markers *in vivo* via ELISA, as well as blood vessel density and area quantified via CD31 staining. **(G)** Representative images of the implanted hydrogels, H&E staining, and immunohistochemical and immunofluorescence staining of CD31 after implantation for 14 days. Scale bar: 1 mm (digital photos) and 50 μm (H&E staining, immunohistochemical, and immunofluorescence staining of CD31). **(H)** Schematic illustration of microenvironmental enhancement and immune modulation in tissue regeneration and repair. Data are expressed as mean ± SD (n = 3). Significant differences compared to the SC group are indicated by *P < 0.05 and **P < 0.01. Significant differences compared to the SC/MTZ+NIR group are indicated by ^#^P < 0.05 and ^# #^P < 0.01.

Building on the observed *in vitro* cytocompatibility, anti-inflammatory, and immunomodulatory properties, we investigated the foreign-body reactions and biological effects of the hydrogels in a mouse subcutaneous implantation model. First, the photothermal performance of the SC/MTZ hydrogel was evaluated, as illustrated in **Figure 10A**. The temperature change curve and infrared thermal images showed that the surface temperature of the mice in the SC/MTZ+NIR group reached 42 ± 1 °C after 5 min of NIR exposure, confirming the *in vivo* photothermal conversion of the hydrogel.

It has been demonstrated that biomaterial implantation is typically associated with foreign body responses, triggering inflammation, fibrous encapsulation, and compromised tissue function. The development of fibrous capsules at the material-tissue interface significantly hinders integration, potentially leading to implant loosening or failure 67. Two weeks post-treatment, both H&E and MST staining indicated fibrous capsule formation around subcutaneous tissue in the SC hydrogel-treated group, which was attributed to immune rejection **(Figure 10B)**. In comparison, the SC/MTZ and SC/MTZ+NIR hydrogels induced much less collagen deposition than the SC hydrogels. The SC/MTZ+NIR group exhibited sparse collagen fibers, whereas dense collagen fibers encased the SC hydrogel. Additionally, significant host cell infiltration was observed in the SC/MTZ hydrogel-treated group, which became more pronounced following mild photothermal stimulation **(Figure 10C-D)**. The SC/MTZ hydrogel's interconnected porous structure, increased surface roughness, hydrophilicity, and structural stability facilitate early cell infiltration post-implantation. The SC/MTZ+NIR group showed the highest host cell infiltration into the hydrogel matrix, facilitated by spatiotemporal, mild, local hyperthermia induced by gentle NIR irradiation. These findings indicate that combining the SC/MTZ hydrogel with mild heat treatment effectively reduces the foreign body response and enhances tissue-hydrogel integration *in vivo*.

**Figure 10E** illustrates the inflammatory status of the surrounding tissues, as assessed through immunofluorescence and immunohistochemical staining. The MTZ heterojunctions demonstrated outstanding antioxidant capabilities, leading to a significant decrease in intracellular ROS levels in both the SC/MTZ and SC/MTZ+NIR groups. Consequently, the expression of the proinflammatory markers iNOS and TNF-α was markedly decreased, which was attributed to diminished oxidative stress-induced damage** (Figure S27)**. Consistent with the *in vitro* findings, SC/MTZ+NIR treatment reduced iNOS expression and increased CD206 expression, indicating a shift from the M1 to the M2 phenotype. TNF-α, a proinflammatory marker, was prominently expressed in the SC hydrogel group. Its expression decreased progressively with the incorporation of MTZ heterojunctions and the application of mild NIR irradiation in the SC/MTZ and SC/MTZ+NIR groups. Conversely, IL-10 expression, an anti-inflammatory marker, was highest in the SC/MTZ+NIR group, suggesting a notable decrease in inflammation and an increase in anti-inflammatory activity. Monocytes are recruited to bone injury sites during tissue repair and differentiate into M1 and M2 macrophages. These macrophages facilitate angiogenesis, MSC recruitment, and osteogenic differentiation by releasing various inflammatory cytokines 97. Therefore, surrounding tissues are collected to assess the effects of hydrogels on inflammatory cytokine secretion within the repair microenvironment. **Figure 10F** illustrates that both the SC/MTZ and SC/MTZ+NIR groups presented significantly lower levels of proinflammatory cytokines (TNF-α and IL-1β) and greater levels of anti-inflammatory cytokines (IL-10 and IL-4) than the SC hydrogel group, demonstrating the strong capacity of the SC/MTZ hydrogel to modulate inflammation under NIR irradiation. Continuous secretion of proinflammatory cytokines like TNF-α and IL-1β disrupts the osteoblast-osteoclast balance, promoting osteoclast activity and increasing bone resorption 98. TNF-α suppresses BMSC osteogenic differentiation via NF-κB pathway activation, whereas IL-6 enhances Notch signaling to increase MMP-13 synthesis, promoting osteoclast differentiation and maturation. IL-10 and IL-4 are key cytokines that promote macrophage polarization toward the M2 phenotype, suggesting significant anti-inflammatory and tissue repair potential 99. M2 macrophages enhance bone repair by secreting anti-inflammatory cytokines, immunosuppressive agents, and osteogenesis-promoting factors like BMP-2 and TGF-β, which collectively reduce inflammation and support bone regeneration 45. This result aligns with increased macrophage polarization and decreased inflammation at the bone defect site. These findings suggest that the mild photothermal-assisted SC/MTZ hydrogel effectively reduces the inflammatory response, encourages M2 macrophage polarization, and shortens the early post-implantation inflammatory phase.

The revascularization of the surrounding tissue was subsequently evaluated through macroscopic observation, H&E staining, and immunohistochemical and immunofluorescence staining. As shown in **Figure 10G**, the SC/MTZ and SC/MTZ+NIR groups presented well-formed vascular networks, with a greater number of blood vessels observed in the SC/MTZ+NIR group. Quantitative analysis revealed that the SC/MTZ+NIR group promoted the development of a dense vascular network in the injured regions, supplying crucial nutrients and oxygen for tissue repair. Continuous blood flow is vital for cell survival, nutrient transport, and metabolite clearance, thereby facilitating proliferation, differentiation, and osteogenesis 72. In contrast, the SC group presented the lowest degree of vascularization, primarily due to acute inflammatory reactions and limited bioactivities. Immunohistochemistry and immunofluorescence staining revealed that the SC/MTZ+NIR group had the highest number of CD31-positive cells **(Figure 10F)**, indicating that the SC/MTZ hydrogel effectively reconstructed the microvascular network and enhanced vascular formation in the injured areas under mild NIR irradiation. In numerous studies, mild thermal stimulation has been shown to increase local blood flow and oxygen tension, improve metabolism, and promote angiogenesis and cell migration, thereby combating local inflammation after bone injury 100. The combined effects of the SC/MTZ hydrogel and local mild hyperthermia significantly contributed to macrophage reprogramming from the M1 to the M2 phenotype, potentially enhancing endothelial cell migration, recruitment, and vascularization. These outcomes confirmed that the SC/MTZ hydrogel promoted the formation of new vascular networks in the implanted areas under NIR irradiation. In conclusion, the results of the *in vivo* subcutaneous implantation study indicate that SC/MTZ+NIR treatment effectively reduces fibrous capsule formation, adjusts the M2/M1 macrophage ratio, and enhances the expression of the anti-inflammatory factors IL-10 and IL-4. This process reduces inflammation, promotes blood vessel formation, and facilitates tissue regeneration, offering significant potential for bone defect repair **(Figure 10H)**. We conducted initial *in vivo* biosafety evaluations to examine the short-term biocompatibility of the hydrogel system. Four weeks post-implantation, the heart, liver, spleen, lung, and kidney were harvested for H&E staining. The results indicated that there were no significant adverse effects on major internal organs following material implantation or NIR exposure **(Figure S28)**. Moreover, there was no apparent difference in the biochemical indicators of the collected blood samples, indicating good biocompatibility of the composite hydrogel with/without mild-temperature photothermal treatment.

### *In vivo* assessment of the effects on ROS scavenging and immune modulation

**Figure 11:**
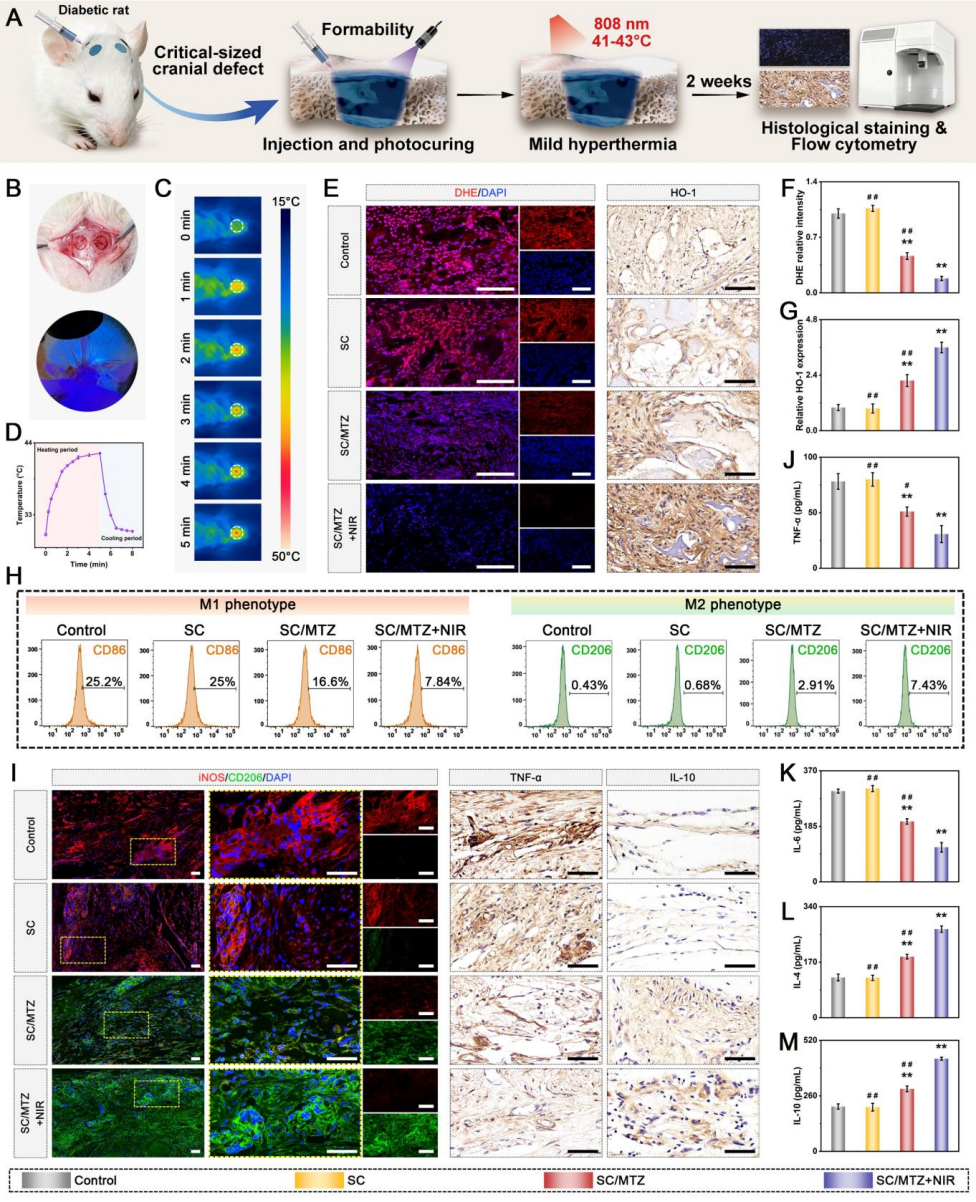
** Immune modulation in the initial phase of bone healing. (A)** Diagram depicting the therapeutic procedure applied in the animal studies. **(B)** Photographs depicting the surgical process for creating a critical-size cranial defect and administering the hydrogel precursor solution. **(C-D)** Real-time infrared thermal imaging and temperature profiles of rats with SC/MTZ hydrogel implants subjected to NIR irradiation (808 nm, 0.5 W/cm²) for 5 min. **(E-G)** Representative images and quantitative analysis of DHE and HO-1 immunohistochemical staining for each group. Scale bar: 100 μm (DHE staining) and 50 μm (immunohistochemical staining of HO-1). **(H)** Flow cytometry was used to measure the expression of CD86 and CD206, markers specific for M1 and M2 macrophages, respectively. **(I)** Representative immunofluorescence images of iNOS and CD206, along with immunohistochemical staining for TNF-α and IL-10, are shown for each group. Scale bar: 50 μm. **(J-M)** Expression of inflammatory factors in regenerated tissues was assessed by ELISA. Data are expressed as mean ± SD (n = 3). Significant differences compared to the control group are indicated by *P < 0.05 and **P < 0.01. Significant differences compared to the SC/MTZ+NIR group are indicated by ^#^P < 0.05 and ^# #^P < 0.01.

These findings indicate that the photoactivated SC/MTZ hydrogel system efficiently reduces intracellular ROS, shields cells from oxidative stress damage, and enhances cell adhesion, proliferation, migration, osteogenic differentiation, and angiogenesis. Diabetic rats were created by administering streptozotocin (STZ) intraperitoneally at 55 mg/kg, maintaining fasting blood glucose levels above 16.7 mmol/L for two weeks. **Figure 11A** illustrates the detailed procedure and schematic of the *in vivo* treatment.

The persistent inflammatory environment at diabetic injury sites induces oxidative stress, which can be mitigated by lowering ROS levels, thereby restoring the balance of the body's antioxidant system 99. To investigate the *in vivo* effects of hydrogels on ROS scavenging and immunomodulation in diabetes, diabetic rats were induced by intraperitoneal STZ injection, and critical-sized cranial defects (Φ = 5 mm) were established as outlined in prior research 101. **Figure 11B** demonstrates that the hydrogel, characterized by its superior injectability and shape adaptability, can be minimally invasively injected into defect sites. The subsequent UV light-induced *in situ* gelation highlights its promising application for irregular bone defects of various sizes and locations. **Figure 11C-D** illustrates that the temperature at the cranial defect sites in rats in the SC/MTZ+NIR group rose to 42 ± 1 °C within 5 min, as shown by real-time infrared thermal images and photothermal curves. This aligns with the initial assessment of the photothermal effects, confirming the remarkable photothermal conversion capability *in vivo*. Local warming therapy at approximately 42 °C enhances blood circulation, increases oxygen availability, and accelerates osteoblast differentiation and bone mineralization 76.

Diabetic conditions increase oxidative stress and excessive ROS accumulation, fostering localized inflammation and hindering bone regeneration. Furthermore, compromising the natural antioxidative defense system does not mitigate the rise in oxidative stress 4. Therefore, addressing ROS elimination, inflammation suppression, and oxidative stress balance is essential for treating diabetic bone defects. Two weeks post-implantation, DHE staining was performed to assess the ROS levels at the defect sites **(Figure 11E)**. In the control group, prominent fluorescence signals appeared, indicating increased ROS content at the defect site, which was primarily due to severe oxidative stress caused by diabetes mellitus *in vivo*. The SC group showed ROS levels similar to those of the control group, suggesting limited ROS scavenging capacity. The SC/MTZ hydrogel significantly reduced oxidative stress in diabetic rats, with the SC/MTZ+NIR group showing the lowest local ROS signal intensity **(Figure 11F)**. These findings suggest that combining mild photothermal stimulation with MTZ loading results in the most effective alleviation of oxidative stress.

Moreover, the level of MDA, a natural product of lipid oxidation, was measured to assess oxidative stress in the defect area. Consistent with the control group, the SC/MTZ group showed reduced MDA levels, particularly after NIR treatment **(Figure S29)**, suggesting significant *in vivo* inhibition of the oxidative stress response. Aligned with our *in vitro* findings, the photoactivated SC/MTZ hydrogel demonstrated strong antioxidant capabilities, effectively reducing oxidative stress during bone healing in diabetic conditions. This study also investigated the activity of the antioxidant enzyme HO-1, which is part of the innate defense system. **Figure 11E** demonstrates that HO-1 expression was significantly greater in the MTZ-incorporated hydrogel-treated groups than in the control and SC groups. The quantitative analysis confirmed that among the three hydrogel-treated groups, the SC/MTZ+NIR group showed the most significant increase in HO-1 expression **(Figure 11G)**. Hou *et al.* reported similar findings, suggesting that NIR-mediated mild hyperthermia combined with polyphenol-modified bone scaffolds could upregulate HO-1 expression, leading to reduced ROS levels and mitigated inflammatory responses during bone repair 28. The concurrent changes in HO-1 expression, ROS, and MDA levels suggest that MTZ nanosheets combined with mild thermal stimulation enhance antioxidant activity and reduce oxidative stress *in vivo*, supporting tissue repair and regeneration.

Chronic inflammation and disrupted immune balance significantly impede the healing of bone defects, especially in patients with diabetes mellitus. In the initial phase of bone repair, M1 macrophages (proinflammatory) convert to M2 macrophages (anti-inflammatory), facilitating the release of anti-inflammatory factors and pro-healing cytokines, thereby supporting MSC recruitment, revascularization, and osteogenic differentiation. Considering the predominant role of macrophages in immune modulation and bone repair, especially during the initial healing period of bone defects, we conducted flow cytometry to quantify M1 and M2 macrophages and assess macrophage reprogramming **(Figure 11H)**. SC/MTZ hydrogel treatment reduced the number of CD86^+^ cells and increased the number of CD206^+^ cells, particularly when combined with mild heat stimulation, which may help alleviate inflammation. These findings suggest that our proposed combined treatment strategy effectively facilitates macrophage transition from M1 to M2, thereby reducing the inflammatory response. This effect may result from the ROS-scavenging and immunomodulatory properties of MTZ heterojunctions and mild photothermal stimulation. Immunofluorescence staining for iNOS (M1 macrophage marker) and CD206 (M2 macrophage marker) was performed at the defect sites post-implantation to investigate the *in vivo* immunomodulatory effects of the hydrogels** (Figure 11I)**. Both the control and SC groups presented a high number of M1 macrophages and a low number of M2 macrophages. The macrophages were predominantly M1, with a significant increase in their proportion under diabetic conditions. Compared with the other groups, the SC/MTZ and SC/MTZ+NIR groups showed a notably reduced number of M1 macrophages and an increased number of M2 macrophages, indicating significant polarization toward the M2 phenotype **(Figure S30)**. Our findings showed that while the defect sites in the control and SC groups remained in the inflammatory stage, those in the SC/MTZ and SC/MTZ+NIR groups progressed to the subsequent healing stage. These findings align with *in vitro* experimental data, further confirming the synergistic effects of MTZ heterojunctions and mild hyperthermia on immunomodulatory activity. The SC/MTZ+NIR group exhibited the highest M2 macrophage percentage and M2 to M1 macrophage ratio, suggesting that SC/MTZ+NIR treatment may mitigate inflammation and enhance M2 macrophage polarization. These findings align with earlier conclusions, highlighting the translational potential of NIR-driven SC/MTZ hydrogels in advanced bone tissue engineering.

To confirm the transition between healing stages, the expression levels of inflammation-related markers, including TNF-α and IL-10, associated with macrophage polarization, were assessed by immunohistochemistry and immunofluorescence. **Figure 11I and Figure S30** demonstrate that TNF-α expression levels are significantly lower in the SC/MTZ and SC/MTZ+NIR groups than in the control and SC groups, with the SC/MTZ+NIR group exhibiting the lowest levels. Additionally, both SC/MTZ-treated groups showed increased IL-10 expression, with the highest levels in the SC/MTZ+NIR group **(Figure S31)**, suggesting an alleviated inflammatory response and reduced oxidative stress. The photoactivated SC/MTZ hydrogel demonstrated potent antioxidant and anti-inflammatory effects, effectively promoting M2 macrophage polarization and reducing proinflammatory cytokine expression. The potent immunomodulatory activity of SC/MTZ+NIR appears to modulate inflammatory cytokine production, promoting rapid bone tissue regeneration by reducing the proinflammatory response. We assessed cytokine levels in the samples via ELISA two weeks after implantation** (Figure 11J-M)**. Consistent with the *in vivo* immunofluorescence and immunohistochemical findings, the MTZ-contained hydrogel-treated groups presented significant reductions in the levels of the proinflammatory factors TNF-α and IL-6, whereas the levels of the anti-inflammatory factors IL-10 and IL-4 increased, especially in the SC/MTZ+NIR group. The enhanced anti-inflammatory effect is mainly due to the targeted release of TA and Zn^2+^ under acidic conditions and under photothermal stimuli, which promote rapid ROS clearance, reduce inflammation, and promote macrophage transition from the M1 to the M2 phenotype. These findings confirmed our research outcomes and hypotheses, indicating that the diabetic defect site entered the inflammation-suppression stage after SC/MTZ+NIR treatment, promoting bone healing. The photoactivated SC/MTZ hydrogel enhanced the metabolism of excess oxidation products in diabetic bone defect areas, promoted M2 macrophage polarization, and reduced inflammation, thereby creating a supportive immune microenvironment for tissue regeneration.

### *In vivo* evaluation of endogenous stem cell recruitment and revascularization

**Figure 12:**
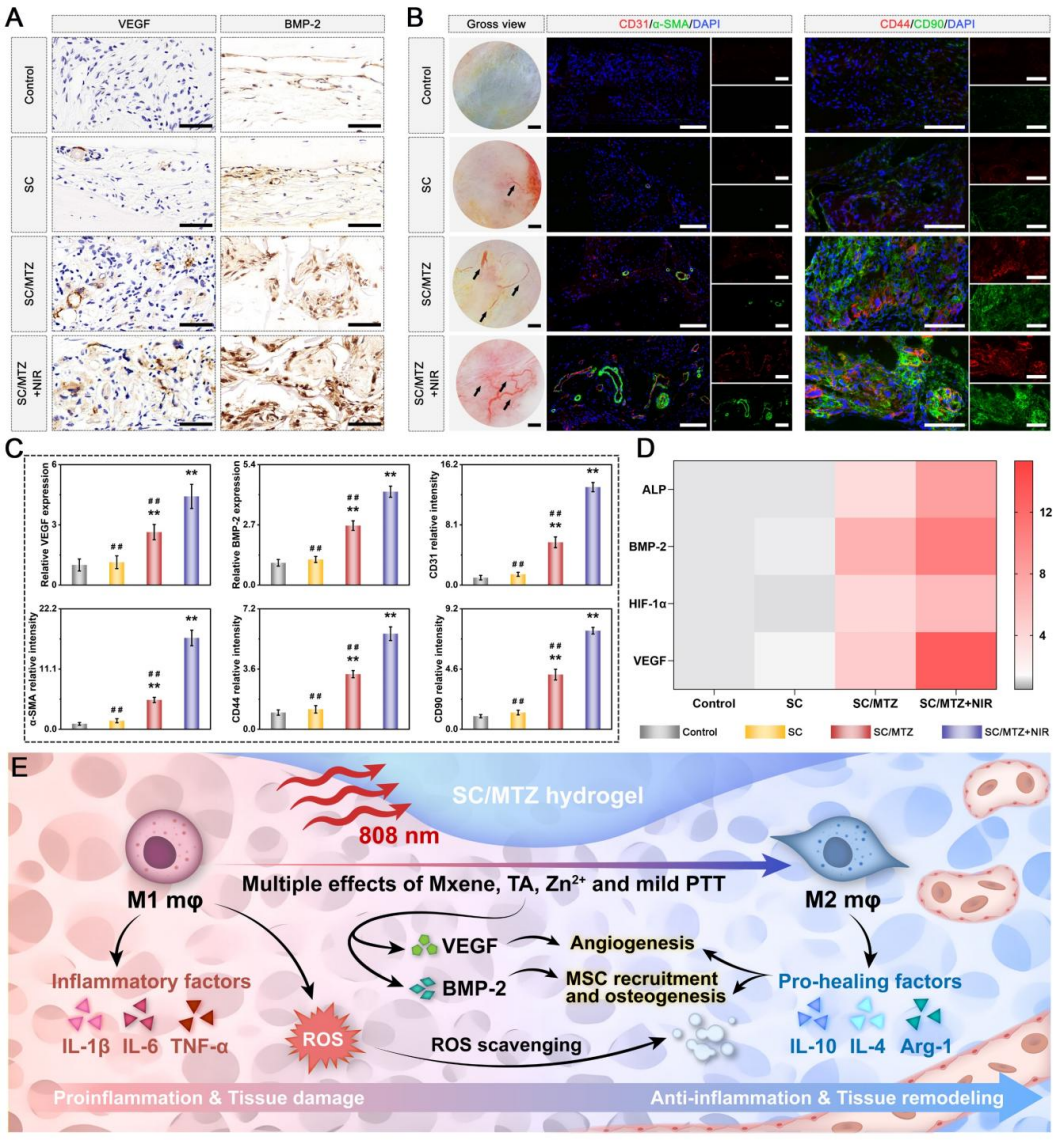
** Bone repair in the intermediate phase involves both osteogenesis and angiogenesis. (A)** Representative images of immunohistochemical staining for VEGF and BMP-2 in each group. Scale bar: 50 μm. **(B)** Photographs of the defect area and immunofluorescence staining for CD31, α-SMA, CD44, and CD90 across all groups. Scale bar: 500 μm (gross view) and 100 μm (immunofluorescence staining). **(C)** Quantitative evaluation of immunohistochemical and immunofluorescence staining. **(D)** Heatmap analysis of osteogenic and angiogenic markers in different experimental groups. **(E)** Diagram depicting how SC/MTZ+NIR enhances osteogenesis and angiogenesis within the diabetic bone microenvironment. Data are expressed as mean ± SD (n = 3). Significant differences compared to the control group are indicated by *P < 0.05 and **P < 0.01. Significant differences compared to the SC/MTZ+NIR group are indicated by ^#^P < 0.05 and ^# #^P < 0.01.

Bone is a richly vascularized tissue, and its vascular network plays a vital role in supplying nutrients and oxygen, removing waste products, and maintaining tissue metabolism 97. Effective bone healing necessitates a conducive microenvironment enriched with proangiogenic and pro-osteogenic factors to facilitate endogenous stem cell recruitment and revascularization. BMP-2 and VEGF are key inducers of osteogenesis and angiogenesis, facilitating blood vessel formation and bone regeneration *in vivo* 79. Following reductions in excessive inflammation and oxidative stress, we examined the potential of mild hyperthermia-assisted SC/MTZ hydrogels to enhance the regenerative microenvironment at bone defect sites in diabetic rats during the initial phase of bone repair. **Figure 12A** demonstrates that immunohistochemical staining indicated significantly elevated BMP-2 and VEGF expression in the SC/MTZ and SC/MTZ+NIR groups, while the control and SC groups exhibited the lowest expression levels. The SC/MTZ+NIR group, aided by mild NIR irradiation, exhibited the highest BMP-2 and VEGF levels among all the groups** (Figure 12C)**, indicating significant potential to enhance osteogenic differentiation and vascularization in bone repair. This could be due to the beneficial immune microenvironment created by the combined effects of the SC/MTZ hydrogel and mild heat stimulation. Research indicates that a prompt shift from the M1 to the M2 phenotype generates beneficial cytokines and triggers rapid angiogenesis and osteogenesis, establishing a solid basis for the *in situ* restoration of cranial defects 98. Research indicates that the recruitment and migration of endogenous stem cells, specifically BMSCs, to bone defect areas are vital for bone healing. Revascularization supports cellular infiltration and tissue remodeling by supplying nutrients and directing tissue ingrowth into the implanted scaffold 102. **Figure 12B** illustrates minimal blood vessel invasion in both the control and SC groups, suggesting inadequate revascularization. In contrast, blood vessels were obvious around the bone defect regions in the MTZ-incorporated hydrogel groups. Immunofluorescence staining confirmed dense populations of CD31- and α-SMA-positive cells in the SC/MTZ and SC/MTZ+NIR groups **(Figure 12B-C)**, indicating more mature vascular structures than in the other groups. The SC/MTZ+NIR group showed increased vascularization, as indicated by increased microvessel density **(Figure 12C)**, demonstrating that the SC/MTZ hydrogel combined with mild heat stimulation enhances angiogenesis in diabetic bone defect areas. The mildly acidic conditions at diabetic bone defect sites, along with initial NIR exposure, increased the release of bioactive Zn^2+^ ions from the hydrogel, promoting faster angiogenesis. On-demand mild thermal stimulation can increase local blood flow and oxygen tension, thereby improving metabolism and facilitating neovascularization at the site of ischemia 12. These data indicate that the anti-inflammatory microenvironment positively influences revascularization, underscoring the photoactivated SC/MTZ hydrogel's enhanced capacity to facilitate the regeneration of functional blood vessels. In addition to exceptional proangiogenic effects, the SC/MTZ hydrogel demonstrated excellent performance in guiding the recruitment and homing of endogenous MSCs. **Figure 12B-C** illustrates that at 4 weeks post-surgery, the SC/MTZ group exhibited a significantly higher presence of MSCs (CD44^+^ and CD90^+^) in the bone defect area than the other groups, particularly after mild photothermal treatment. These results align with *in vitro* evidence showing that SC/MTZ+NIR promotes the migration and recruitment of BMSCs and osteoblasts, potentially aiding in the accelerated bone formation observed with SC/MTZ+NIR treatment. Modulating macrophage phenotypes to alter the immune microenvironment can accelerate stem cell migration to damaged areas and promote osteogenic differentiation, thereby assisting bone regeneration 30.

These *in vivo* results underscore the exceptional ability of SC/MTZ+NIR to rapidly initiate early angiogenesis and osteogenesis in rat cranial defects via *in situ* recruitment of MSCs to the injury site and subsequent acceleration of osteo-differentiation under diabetic conditions. The rapid formation of capillaries enhanced the recruitment of endogenous stem cells to the defect site, aiding bone repair. Gene expression levels of early-stage osteogenesis markers (ALP and BMP-2) and angiogenesis markers (HIF-1α and VEGF) were assessed using qRT-PCR. **Figure 12D** shows that while the SC/MTZ hydrogel group exhibited higher gene expression than the control and SC groups, the SC/MTZ+NIR group demonstrated the highest relative gene expression, indicating that on-demand mild hyperthermia enhanced the osteoinductive and angiogenic potential. **Figure 12E** outlines the potential mechanisms for inflammation regression and its subsequent impact on angiogenesis and osteogenesis. These findings align with those of *in vitro* experiments, demonstrating that the combination of MTZ heterojunctions and mild photothermal stimulation effectively promotes revascularization and osteoinduction, potentially facilitating new vascular and bone formation.

### *In vivo* evaluation of diabetic bone defect repair *in situ*

**Figure 13:**
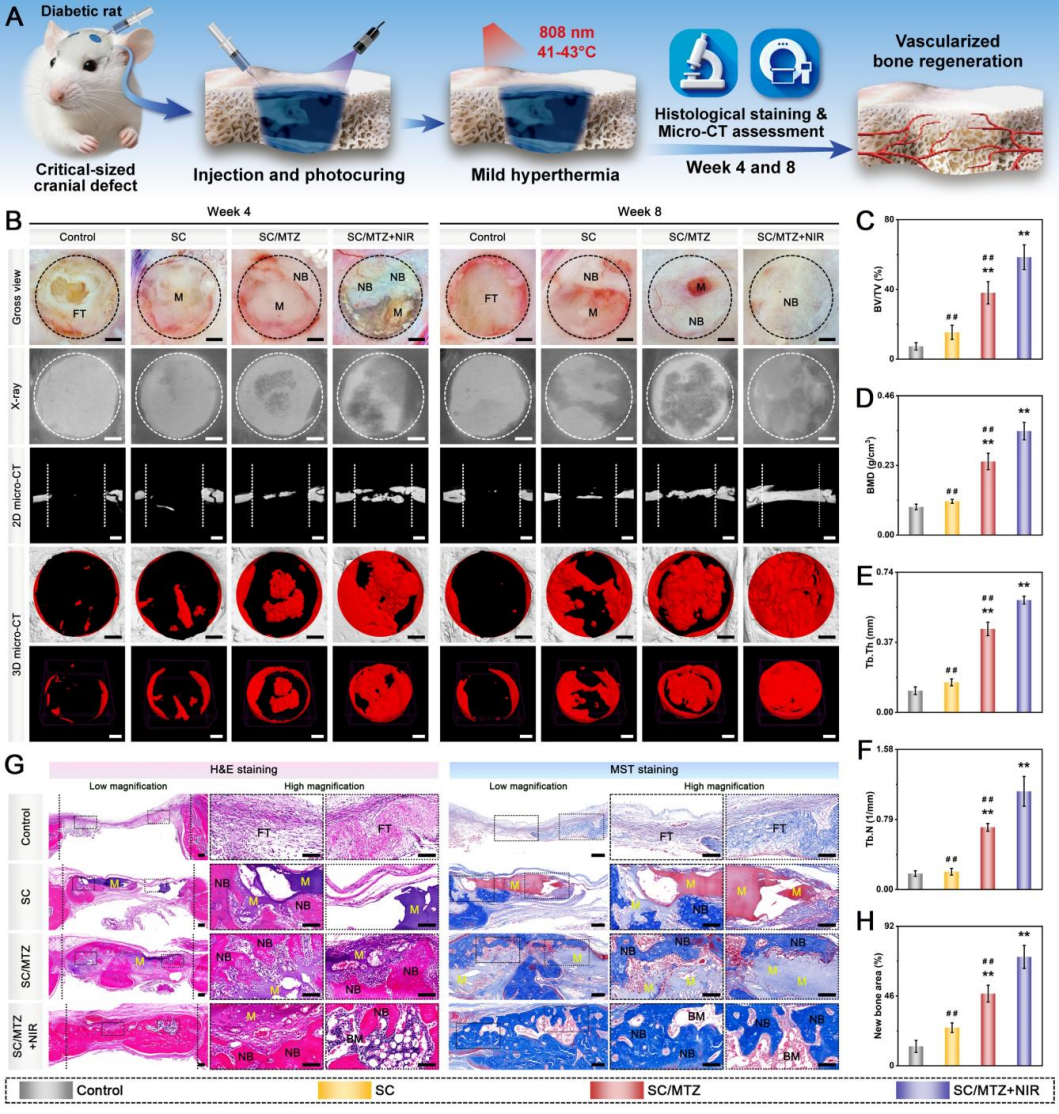
***In vivo* bone repair. (A)** Diagram depicting the creation and management of diabetic skull bone defects. **(B)** Macroscopic examination, 3D reconstruction, and 2D micro-CT imaging of rat cranial defects post-implantation. The white and black dotted lines represent the boundaries between the nascent bone and host bone. Scale bar: 1 mm. **(C-F)** Quantitative statistics of new bone from micro-CT analysis. **(G)** H&E and MST staining images of rat calvarial defect areas. **(H)** Measurement of new bone area using H&E staining conducted 8 weeks post-implantation. M: material residue. FT: fibrous connective tissue. NB: new bone. BM: bone marrow. Scale bar: 200 μm (low-magnification images) and 100 μm (high-magnification images). Data are expressed as mean ± SD (n = 3). Significant differences compared to the control group are indicated by *P < 0.05 and **P < 0.01. Significant differences compared to the SC/MTZ+NIR group are indicated by ^#^P < 0.05 and ^# #^P < 0.01.

This section examines bone regeneration through macroscopic observations and micro-CT analyses at 4 and 8 weeks post-implantation, building on *in vitro* cell experiments, subcutaneous implantation, and *in vivo* immunomodulatory functions. **Figure 13A** illustrates that the hydrogel successfully filled the bone defect, achieving seamless integration with the surrounding host tissue. The hydrogels degraded over time and were replaced by new bone tissue, demonstrating suitable compatibility and biodegradability. Under mild NIR irradiation, the SC/MTZ group demonstrated substantial new bone formation and ingrowth into the hydrogel, effectively covering the defect site and achieving commendable bone regeneration and reconstruction. Similar visualization results were presented in X-ray images and 3D reconstructions via micro-CT analysis **(Figure 13B)**. The findings indicated negligible new bone formation in the control group, with no radiographic evidence of healing during the bone repair process. Defects treated with the SC/MTZ hydrogel showed increased new bone growth and satisfactory integration, with these effects becoming more pronounced over time. These findings indicate that SC/MTZ implantation under diabetic conditions significantly reduces inhibition and enhances bone regeneration. The SC/MTZ+NIR group showed significantly better repair outcomes at both time points, with complete new bone formation in the defect area by 8 weeks. In contrast, the SC and SC/MTZ groups still had visible deficiencies in the central defect areas. 3D-reconstructed micro-CT images provided enhanced visualization of new bone formation across the groups. Compared with the SC and control groups, the MTZ-incorporated hydrogel groups presented better osteoinductive properties, and a substantial amount of new bone tissue was observed in the SC/MTZ+NIR group. Compared with the control group, the SC group showed superior bone regeneration, likely due to the SC hydrogel's biomimetic microstructure and tissue-filling properties, which enhanced its osteoconductivity beyond that of the control group. The findings indicate that combining NIR-mediated on-demand mild hyperthermia with MTZ nanosheets may synergistically enhance bone regeneration. The analysis of BV/TV, BMD, Tb.Th, and Tb.N corroborated the progressive increase in new bone formation within the defect area over time. After 8 weeks, the SC/MTZ+NIR group exhibited significantly higher BV/TV, BMD, Tb.Th, and Tb.N than all other groups, suggesting enhanced bone density and structure **(Figure 13C-F and Figure S32)**. The analysis of Tb.Sp consistently yielded congruent results, indicating that the SC/MTZ hydrogel, combined with gentle NIR irradiation, has beneficial effects on bone regeneration and reconstruction. The SC/MTZ hydrogel demonstrated long-term retention and localized mild thermal stimulation, alongside remarkable antioxidant, anti-inflammatory, and pro-osteo-/angiogenic properties, making it effective for treating diabetic bone defects. Numerous animal and clinical studies have demonstrated that both on-demand and periodic mild hyperthermia can enhance bone regeneration by synergistically promoting immunomodulation and maintaining bone homeostasis 103, 104. These findings indicate that the photoactivated SC/MTZ hydrogel system, by modulating the unbalanced microenvironment, effectively enhances bone repair in defects, achieving optimal mineral density and structural integrity in newly formed bone.

To assess *in vivo* bone repair effects, cranial samples were decalcified, embedded in paraffin, and sectioned. H&E and MST staining were used to confirm the histopathological structures of regenerated bone at the defect sites **(Figure 13G)**. Coronal sections of the cranium, along with partially magnified images at 4 and 8 weeks, revealed newly formed bone, fibrous tissue, and residual materials. H&E staining revealed that the control group primarily formed fibrous tissue in the defects at 4 weeks, with minimal new bone tissue, displaying scattered, discontinuous structures at the defect periphery after 8 weeks. Conversely, progressive bone tissue regeneration was observed in the groups treated with the composite hydrogels over time. Both the SC/MTZ and SC/MTZ+NIR groups showed significant bone regeneration, with the SC/MTZ+NIR group exhibiting superior new bone tissue ingrowth, as indicated by the larger new bone area **(Figure 13H)**. These findings suggest that SC/MTZ+NIR enhances the repair process and promotes substantial mineralized bone formation. Compared with the other groups, the SC/MTZ+NIR group exhibited a dense, orderly lamellar bone distribution within the defects, suggesting enhanced new bone tissue and matrix deposition at 8 weeks. MST staining revealed sparse blue-stained collagen fibers in the original defects of the control group. In contrast, the composite hydrogel-treated groups presented significant collagen fibers, indicating localized new bone formation. The SC/MTZ+NIR groups exhibited abundant, regularly arranged collagen deposits with continuous structures, indicating that the composite hydrogel combined with mild heat stimulation promoted the maturation and mineralization of regenerated bone in the diabetic microenvironment. The SC group exhibited mature bone at the edges, with predominantly fibrous tissue occupying the central region of the bone defects. These findings confirm that SC/MTZ+NIR effectively promotes new bone formation by counteracting diabetes-related osteogenesis inhibition *in vivo*. Eight weeks post-implantation, the MTZ-incorporated hydrogels, characterized by ECM-mimicking structures and bioactive components such as TA and Zn^2+^, gradually degraded and were enveloped by newly formed bone tissue, providing a supportive framework and biochemical cues for bone ingrowth. The bone repair scaffold not only mechanically supports the bone defect site but also promotes cell accommodation, migration, and nutrient exchange due to its appropriate biodegradability. This finding suggests that the photoactivated SC/MTZ hydrogel system effectively promotes endogenous cell ingrowth and improves biomaterial-bone integration, thus expediting bone regeneration and maturation. The combined effects of mild thermal stimulation, ROS scavenging, and M2 macrophage polarization fostered a regenerative microenvironment that regulated cellular activities and enhanced bone healing. In conclusion, the SC/MTZ hydrogel demonstrated superior biocompatibility and degradability *in vivo*, effectively enhancing diabetic bone defect repair and regeneration with mild NIR irradiation.

### *In vivo* evaluation of bone mineralization and remodeling

**Figure 14:**
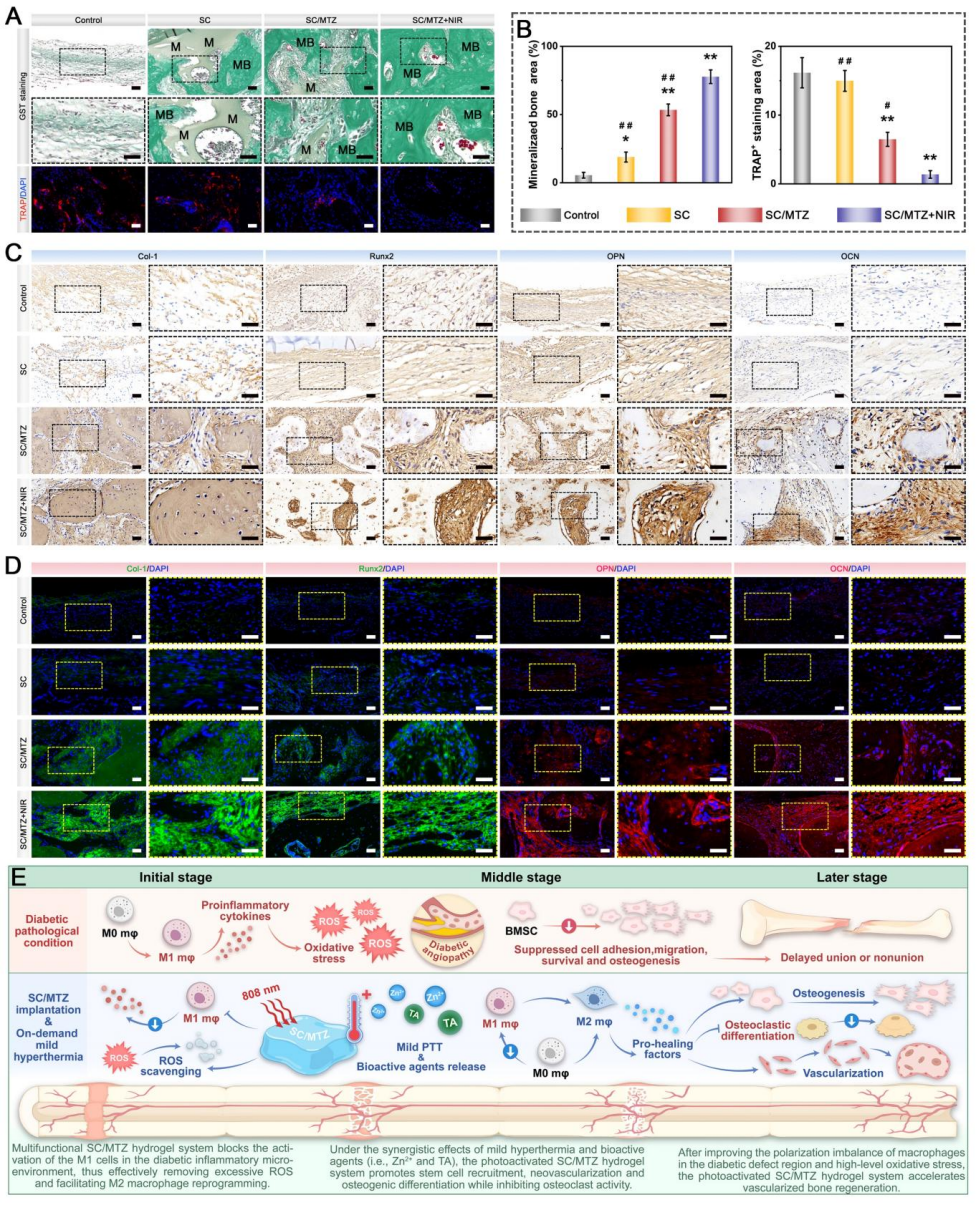
** Bone mineralization and bone resorption during the later stage of bone repair. (A)** Images depicting GST and TRAP staining 8 weeks post-surgery. M: material residue. MB: mature tissue. Scale bar: 50 μm. **(B)** Quantitative analysis of the mineralized bone area via GST staining and TRAP staining. **(C-D)** Immunohistochemical and immunofluorescence analyses of Col-1, Runx2, OPN, and OCN were conducted in the defect region. Scale bar: 50 μm. **(E)** Schematic representation of the mechanism through which SC/MTZ+NIR enhances bone defect healing in diabetic inflammatory environments. Data are presented as the mean ± SD (n = 3). Significant differences compared to the control group are indicated by *P < 0.05 and **P < 0.01. Significant differences compared to the SC/MTZ+NIR group are indicated by ^#^P < 0.05 and ^# #^P < 0.01.

Restoring the osteoclast/osteoblast balance is crucial for maintaining the functional structure of bone during mineralization and remodeling. However, excessive inflammation in the diabetic environment can result in hyperactive osteoclast formation and bone resorption, which impedes functional bone repair 2. Following the correction of macrophage polarization imbalance and elevated oxidative stress in diabetic bone defect areas, it is essential to address the disrupted osteoblast/osteoclast balance and the resulting bone defects to achieve effective tissue regeneration. The results of Goldner's trichrome (GST) staining and TRAP immunofluorescence staining demonstrated the presence of denser mature bone tissue and higher calcification deposition, along with a significant reduction in TRAP-positive osteoclasts in the SC/MTZ group under gentle NIR irradiation **(Figure 14A-B)**. TRAP is a standard marker of osteoclasts, indicating their activity and the extent of bone resorption. The SC/MTZ+NIR group recreated a favorable microenvironment for balancing bone formation and resorption by reducing inflammation and inhibiting osteoclast activity, which is crucial for effective bone regeneration and remodeling. This observation confirmed the synergistic effects of spatiotemporal local mild hyperthermia and MTZ heterojunctions in enhancing mineral deposition, accelerating bone maturation, and inhibiting osteoclast differentiation and formation. The therapeutic efficacy of the composite hydrogels in treating diabetic bone defects was assessed via immunohistochemistry and immunofluorescence staining. **Figures 14C-D** demonstrate that the SC/MTZ hydrogel treatment led to prominent, uniform expression of key osteogenic markers, including Col-1, Runx2, OPN, and OCN, in the defect areas, highlighting its superior osteoinductive properties and capacity to enhance mineralized matrix deposition. In contrast, the expression levels of these osteogenic markers in both the control and SC groups were relatively weak, loose, and unevenly distributed, indicating that osteogenesis and mineralization were severely inhibited in the diabetic inflammatory environment. Compared with the other groups, the SC/MTZ+NIR group presented the highest expression levels of osteogenic markers, suggesting superior osteogenic activity and mineralization. Additionally, immunofluorescence staining revealed that Col-1, Runx2, OPN, and OCN expression levels in the SC/MTZ+NIR group were markedly higher than those in all other groups, consistent with the immunohistochemical results. Quantitative analysis demonstrated significantly larger positive staining areas for Col-1, Runx2, OPN, and OCN in the SC/MTZ+NIR group **(Figure S33)**, indicating improved osteogenic differentiation and mineralization potential of the SC/MTZ hydrogels under mild NIR irradiation, both *in vitro* and *in vivo*. Eight weeks post-implantation, the SC/MTZ+NIR group exhibited restored bone resorption and formation balance, likely due to extended modulation of osteoclast-osteoblast coupling signals. The mild photothermal-assisted SC/MTZ hydrogel system, with integrated ROS-scavenging and immunomodulatory properties, effectively restored the bone regeneration microenvironment. This enhancement promotes angiogenesis-osteogenesis coupling, inhibits osteoclast function, and reduces bone resorption, thereby accelerating bone tissue repair in diabetic inflammatory conditions.

Studies have demonstrated that tissue healing efficiency is closely associated with local inflammatory responses. The immune system undoubtedly plays a critical regulatory role in this complex process. Excessive or dysregulated immune responses can worsen tissue damage and hinder healing, as shown in diabetic bone defect models. In diabetes-related tissue healing, the shift of macrophages from the M1 to the M2 phenotype is disrupted, resulting in prolonged inflammation and slower recovery. Given the pivotal role of immune responses in this context, the SC/MTZ hydrogel platform developed in this study, combined with NIR-triggered mild hyperthermia, exerts exceptional immunomodulatory effects by reprogramming macrophage polarization, thereby promoting tissue healing. On the basis of the literature, multiple plausible mechanisms may account for the observed *in vivo* therapeutic effects, as illustrated in **Figure 14E**. The programmed SC/MTZ hydrogel achieves intelligent, on-demand release of Zn^2+^ ions and TA in response to the combined effects of NIR irradiation (external stimuli) and weakly acidic diabetic conditions (internal stimuli), thereby enabling control of bacterial infection and coordination of improvements in the bone microenvironment. The spatiotemporal local mild hyperthermia-assisted SC/MTZ hydrogel platform enhanced the immune microenvironment by increasing anti-inflammatory cytokines in macrophages and inhibiting osteoclastogenesis. It also directly promoted BMSC osteogenic differentiation and HUVEC angiogenesis, supporting vascularized bone regeneration. Additionally, the SC/MTZ hydrogel, with excellent injectability, suitable mechanical strength, and degradation, can provide bioactive functional support and chemical cues for endogenous cell recruitment, migration, and differentiation, ensuring subsequent angiogenesis and osteogenesis. Altogether, these characteristics synergistically improve the bone defect environment, regulate macrophage polarization, and relieve inflammation, thereby significantly potentiating integrated bone regeneration under diabetic pathological conditions. This versatile hydrogel platform provides valuable insights and practical applications for the treatment of complex bone defects and for advanced bone tissue engineering, facilitating future clinical translation. Notably, in contrast to the majority of MXene-based or ZIF-8 composite scaffolds/hydrogels that are pre-fabricated and rigid, necessitating surgical implantation 59, 105, the SC/MTZ hydrogel developed herein possesses intrinsic injectability. This distinctive characteristic stems from its double-dynamic network, which comprises covalent bonds (formed via free-radial polymerization of double bonds within the SC backbone) and metal coordination bonds (formed between Zn²⁺ released from ZIF-8 and carboxyl/amino groups in CMCS). Such injectability enables minimally invasive delivery and precise conformal filling of irregular bone defects, a pivotal advantage for clinical translatability. By comparison, as documented by Chen *et al.* 55, pre-fabricated scaffolds frequently encounter challenges in achieving gap-free intimate contact with defect sites. Furthermore, the ROS-scavenging capability of the MTZ heterojunction alleviates secondary thermal oxidative stress, a confounding issue that remains unaddressed in conventional photothermal hydrogels 18. Unlike previously reported systems featuring constant or inadequately controlled release profiles 51, the SC/MTZ system exhibits dual responsiveness to external NIR stimulation and internal pathological microenvironments (i.e., acidic pH), thereby conferring temporally and spatially controlled therapeutic effects. Moreover, in contrast to most reported MXene-based hydrogels (which predominantly focus on photothermal antibacterial or conductive functionalities) 106 or ZIF-8 composite scaffolds (which are often engineered for drug delivery or single-ion therapeutic effects) 107, the SC/MTZ platform is uniquely tailored for orchestrated multi-targeted therapy in the complex diabetic bone microenvironment. In conclusion, while existing advanced materials excel in one or two discrete functionalities, the SC/MTZ hydrogel integrates injectable adaptability, stable and intelligent photothermal responsiveness, and elaborately designed multi-component synergy into a single integrated platform. This integrated design aims to break the cycle of oxidative stress, chronic inflammation, and impaired regeneration in diabetic bone defects, suggesting promising advancements in material-based therapeutic strategies.

Despite the promising results above, translating scientific investigations of SC/MTZ hydrogels into clinical practice remains challenging due to regulatory approval requirements and long-term safety assessments. Although the study did not extensively detail the long-term degradation of the hydrogel, positive histological findings and the use of recognized biodegradable materials provide initial evidence of its biocompatibility. It is necessary to comprehensively evaluate its *in vivo* degradation kinetics, chronic biosafety, and translational potential for bone regeneration applications in future research plans. This study primarily validated the efficacy of the SC/MTZ hydrogel in a rat cranial defect model, a non-load-bearing bone site. While this model serves as the standard for initial conceptual validation in bone regeneration research, it does not comprehensively capture the complex biomechanical environment and healing challenges of weight-bearing bones like the femur and tibia. The performance of this material under sustained mechanical stress still needs to be elucidated. Thus, subsequent investigations should cover a broader range of animal models, particularly those involving vertical and irregular bone defects in rabbits, beagle dogs, and miniature pigs.

## Conclusion

In summary, we report a polyphenol-mediated *in situ* modification strategy for preparing a novel multifunctional MTZ heterojunction by depositing ZIF-8 on TA-modified Ti_3_C_2_T_x_ MXene nanosheets. Both *in vitro* studies and transcriptomic assessments have demonstrated that the MTZ heterojunction is a potential therapeutic nanoplatform for combating oxidative stress, immune dysregulation, and osteogenesis inhibition, elucidating its underlying molecular mechanism. To better counteract unfavorable diabetic bone healing conditions, the optimized MTZ heterojunction was incorporated into a physiochemical dual-crosslinked hydrogel network to control bacterial infections, promote revascularization, disrupt the ROS-inflammation feedback loop, and dynamically restore local immunity and bone homeostasis. Our study revealed that uniformly distributed MTZ nanosheets enhanced the physical properties and mechanical strength of the hydrogel, reduced its swelling ratio, and extended its degradation time. Additionally, they facilitate the development of an ECM-mimetic microstructure, support cell growth and adhesion, and promote neovascularization and osseointegration, ultimately improving tissue repair. When injected, the SC/MTZ hydrogel demonstrated controlled mild thermal effects under periodic NIR irradiation and stimuli-responsive drug release, effectively modulating the proinflammatory and oxidative stress microenvironments while maintaining the regenerative potential of reparative cells. The hydrogel, enhanced by mild PTT, demonstrated significant antibacterial and immunomodulatory properties, effectively promoting macrophage polarization toward the anti-inflammatory M2 phenotype. This process inhibits inflammation and the ROS feedback loop, facilitating a timely shift from a proinflammatory state to an anti-inflammatory and pro-healing state. The osteoimmune microenvironment, enhanced by M2 macrophage activation, indirectly supports the osteogenic differentiation and mineralization of BMSCs and pre-osteoblasts, thereby promoting bone regeneration. *In vivo* findings demonstrated the effectiveness of the photoactivated SC/MTZ hydrogel in reducing oxidative stress and inflammation, inducing M2 macrophage polarization, enhancing regenerative processes, including MSC recruitment, vascularization, osteogenesis, and biomineralization, and inhibiting osteoclastogenesis. NIR-driven multifunctional biodegradable hydrogels show significant promise for spatiotemporal control of dynamic tissue regeneration environments, making them strong candidates for treating complex tissue defects in future therapies.

## Supplementary Material

Supplementary figures, table, and movie legends.

Supplementary movie.

## Data Availability

The data underpinning this article are available in the text and supplementary information file. Supplementary data and plot-related data from this paper are accessible from the corresponding author upon reasonable request.
