# Supplementary material for: Photothermal-driven multifunctional injectable hydrogel platform for promoting diabetic bone defect repair through synergistic immunomodulation and bone homeostasis
Source: Theranostics. 2026 Mar 25;16(10):5630–84. doi: 10.7150/thno.130240 (PMC13080832; doi:10.7150/thno.130240)
Supplement: Supplementary file 1 — Supplementary figures, table, and movie legends. [file thnov16p5630s1.pdf]

Supporting Information

Photothermal-driven multifunctional hydrogel platform for promoting diabetic bone defect repair through synergistic immunomodulation and bone homeostasis

Yufan Zhu[#1, 2], Huifan Liu[#3], Zhiqiang Yang[#2], Yaxing He[#2], Ping Wu[4], Lin Cai[*2], Lufeng Yao[*5, 6], Xiaobin Zhu[*2], Minhao Wu[*2]

[1] Department of Radiation and Medical Oncology, Zhongnan Hospital of Wuhan University, 168 Donghu Street, Wuchang District, Wuhan 430071 Hubei, People's Republic of China
[2] Department of Spine Surgery and Musculoskeletal Tumor, Zhongnan Hospital of Wuhan University, 168 Donghu Street, Wuchang District, Wuhan 430071 Hubei, People's Republic of China
[3] Department of Anesthesiology, Research Centre of Anesthesiology and Critical Care Medicine, Zhongnan Hospital of Wuhan University, Wuhan, Hubei, China
[4] State Key Laboratory of Macromolecular Drugs and Large-scale Preparation, School of Pharmaceutical Science, Wenzhou Medical University, Wenzhou 325035, China
[5] Department of foot and ankle Surgery, Ningbo No.6 Hospital, Ningbo, 315040, China
[6] Ningbo Clinical Research Center for Orthopedics, Sports Medicine & Rehabilitation, Ningbo, Zhejiang, China.

[#] These authors contributed equally to this work
* Correspondence should be addressed to:

Dr. Minhao Wu. Email: wuminhao1991@whu.edu.cn
Department of Spine Surgery and Musculoskeletal Tumor, Zhongnan Hospital of Wuhan University, 168 Donghu Street, Wuchang District, Wuhan 430071 Hubei, People's Republic of China

Dr. Xiaobin Zhu. Email: xiaobinzhu@whu.edu.cn
Department of Spine Surgery and Musculoskeletal Tumor, Zhongnan Hospital of Wuhan University, 168 Donghu Street, Wuchang District, Wuhan 430071 Hubei, People's Republic of China

Dr. Lufeng Yao. Email: mdyaolf@163.com
Department of foot and ankle Surgery, Ningbo No.6 Hospital, Ningbo, 315040, China
Ningbo Clinical Research Center for Orthopedics, Sports Medicine & Rehabilitation, Ningbo, Zhejiang, China

Dr. Lin Cai. Email: orthopedics@whu.edu.cn
Department of Spine Surgery and Musculoskeletal Tumor, Zhongnan Hospital of Wuhan University, 168 Donghu Street, Wuchang District, Wuhan 430071 Hubei, People's Republic of China

Supplementary figures and tables

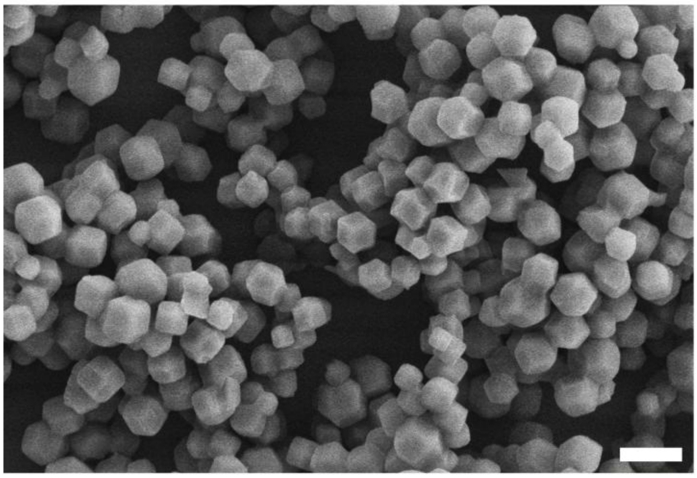

Figure S1. SEM image of ZIF-8. Scale bar: 500 nm.

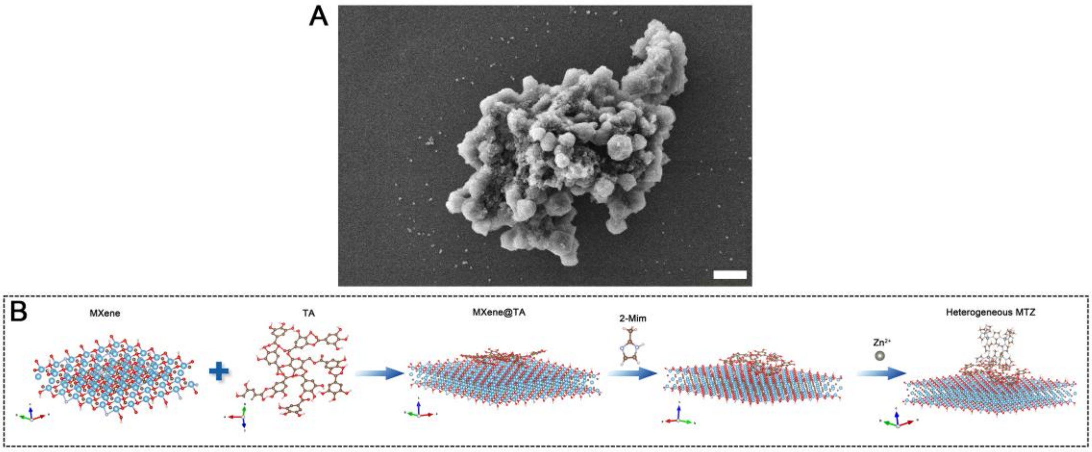

Figure S2. (A) SEM image of MTZ. Scale bar: 100 nm. **(B)** Schematic diagram of the preparation of MTZ.

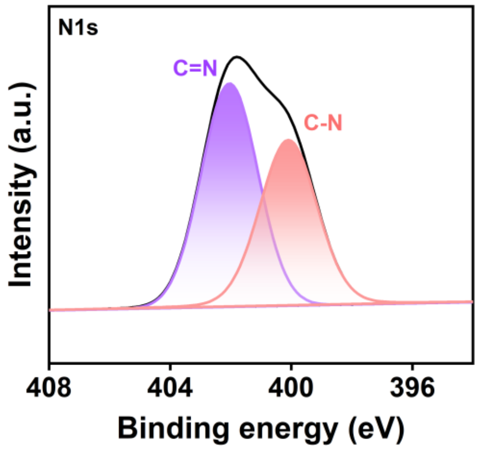

Figure S3. High-resolution XPS spectra of the N1s spectrum for MTZ.

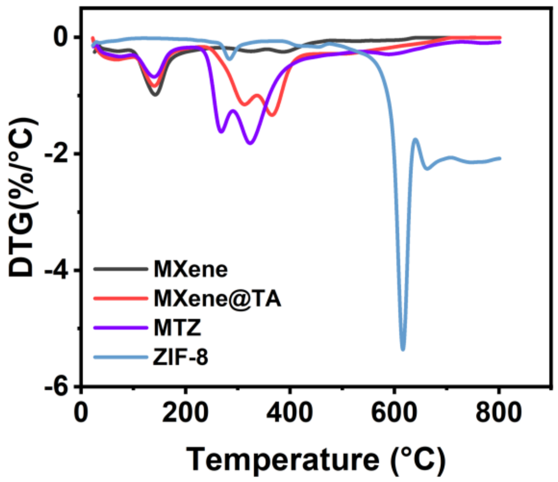

Figure S4. DTG curves of the different samples.

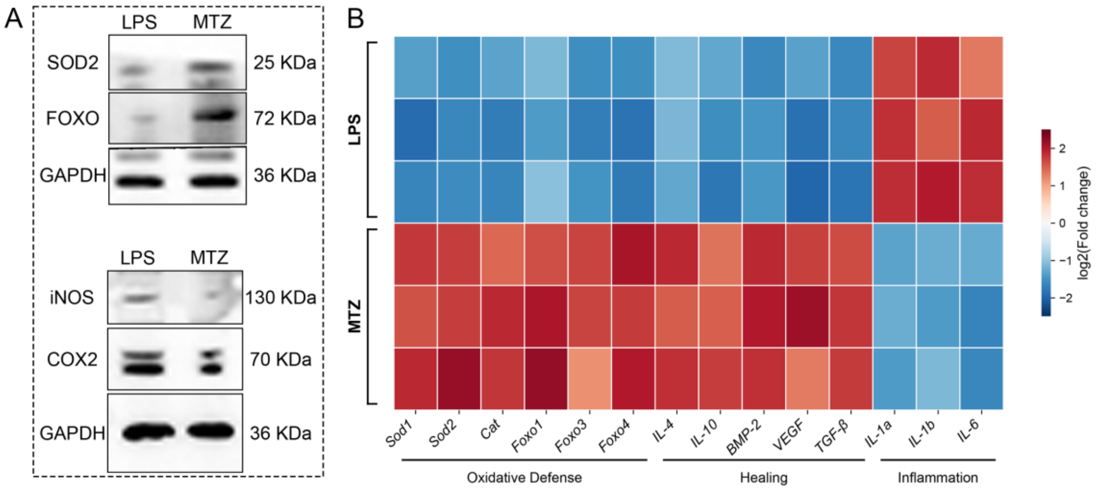

Figure S5. (A) Western blot analysis of the FOXO and TNF signaling pathways in LPS-treated macrophages cocultured with or without MTZ. **(B)** Heatmap analysis of DEGs

involved in anti-oxidative stress, tissue repair, and inflammation.

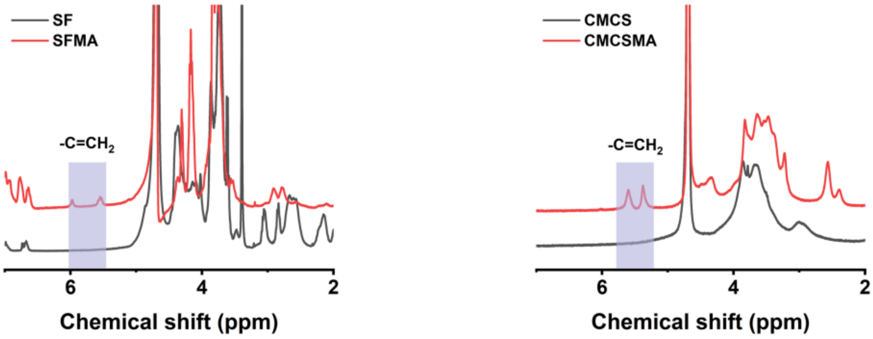

Figure S6. ^{1}H NMR spectra of SF, SFMA, CMCS, and CMCSMA.

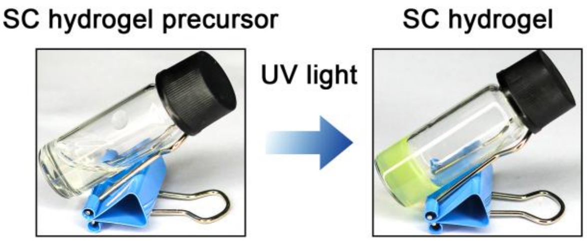

Figure S7. Photograph showing transition from SC prepolymer to hydrogels.

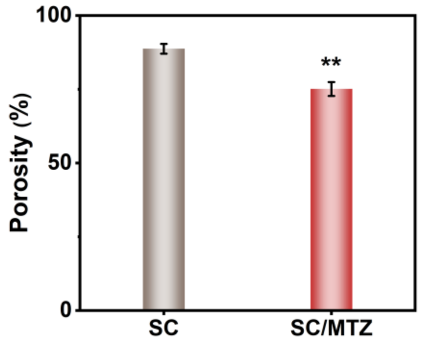

Figure S8. The porosity of SC and SC/MTZ, respectively. Data are presented as the mean ± SD (n = 3). *P < 0.05 and **P < 0.01 indicate significant differences compared with the SC group.

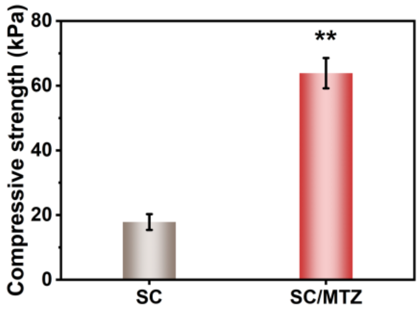

Figure S9. Compressive strength of the hydrogels. Data are presented as the mean ± SD (n = 3). *P < 0.05 and **P < 0.01 indicate significant differences compared with the SC group.

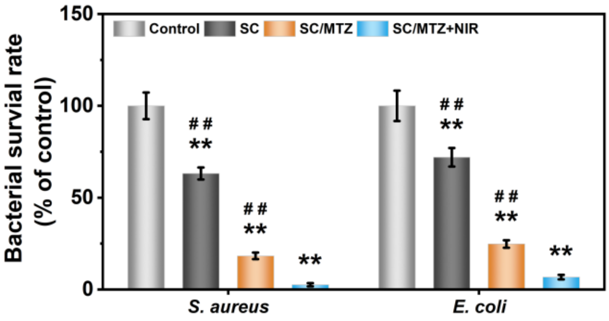

Figure S10. Survival ratios of *E. coli* and *S. aureus* in different groups. Data are presented as the mean ± SD (n = 3). *P < 0.05 and **P < 0.01 indicate significant differences compared with the control group. #P < 0.05 and ##P < 0.01 indicate significant differences compared with the SC/MTZ+NIR group.

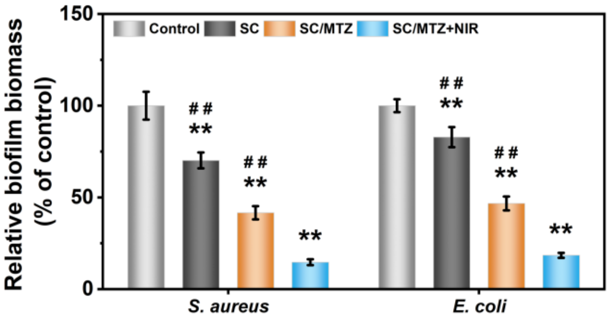

Figure S11. Quantification of biofilm mass. Data are presented as the mean ± SD (n = 3). *P < 0.05 and **P < 0.01 indicate significant differences compared with the control group. #P < 0.05 and ##P < 0.01 indicate significant differences compared with the SC/MTZ+NIR group.

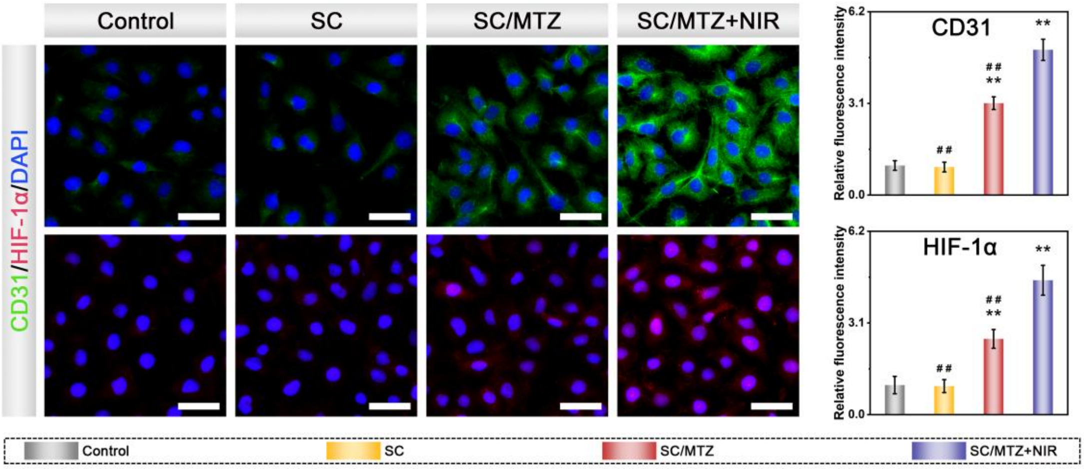

Figure S12. Representative images and corresponding quantitative analysis of immunofluorescence staining of CD31 and HIF-1α. Scale bar: 50 μm. Data are presented as the mean ± SD (n = 3). *P < 0.05 and **P < 0.01 indicate significant differences compared with the control group. #P < 0.05 and ##P < 0.01 indicate significant differences compared with the SC/MTZ+NIR group.

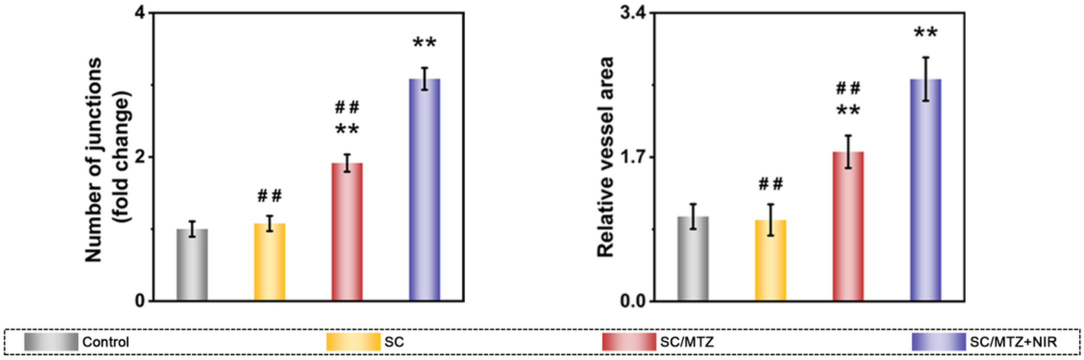

Figure S13. Relative number of tube junctions and blood vessel area in each group. Data are presented as the mean ± SD (n = 3). *P < 0.05 and **P < 0.01 indicate significant differences compared with the control group. #P < 0.05 and ##P < 0.01 indicate significant differences compared with the SC/MTZ+NIR group.

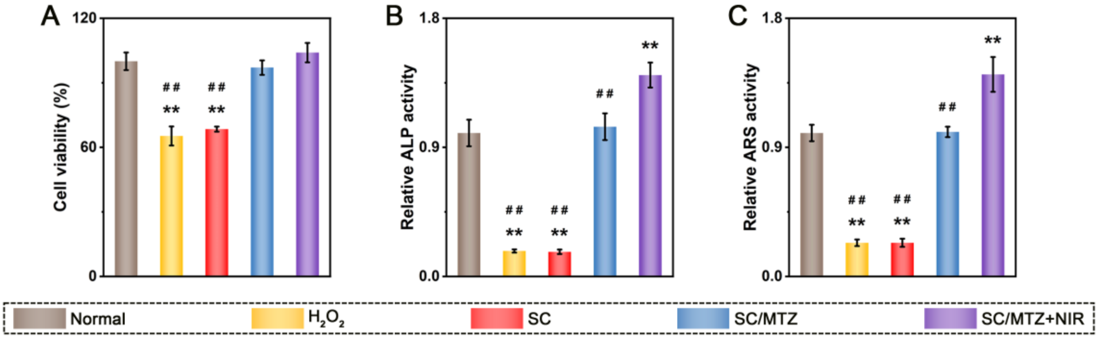

Figure S14. (A) CCK-8 assay of the BMSCs in each group. **(B-C)** Quantitative analysis of

ALP staining and ARS staining. Data are presented as the mean ± SD (n = 3). *P < 0.05 and **P < 0.01 indicate significant differences compared with the normal group. #P < 0.05 and ##P < 0.01 indicate significant differences compared with the SC/MTZ+NIR group.

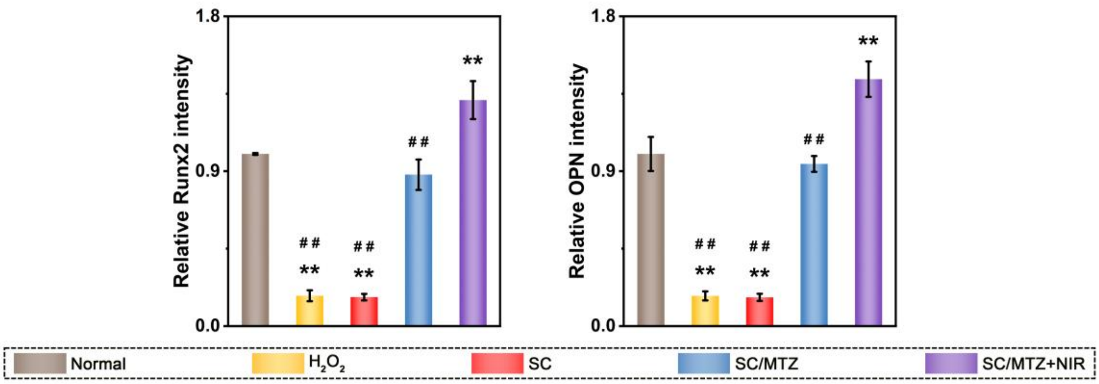

Figure S15. Quantitative analysis of immunofluorescence staining in each group. Data are presented as the mean ± SD (n = 3). *P < 0.05 and **P < 0.01 indicate significant differences compared with the normal group. #P < 0.05 and ##P < 0.01 indicate significant differences compared with the SC/MTZ+NIR group.

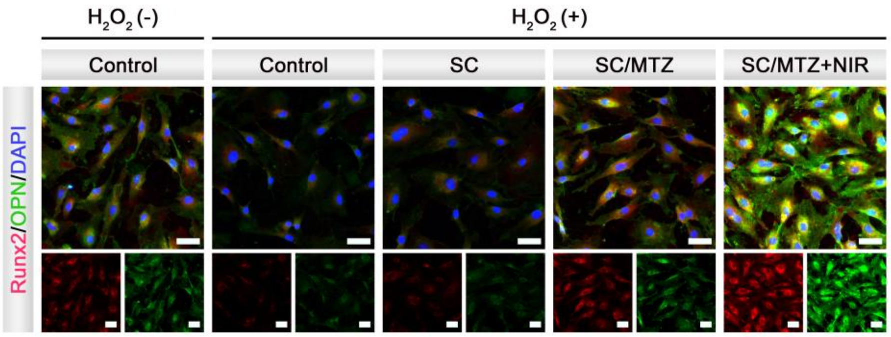

Figure S16. Immunofluorescence staining of Runx2 and OPN in MC3T3-E1 cells after various treatments. Scale bar: 50 μm.

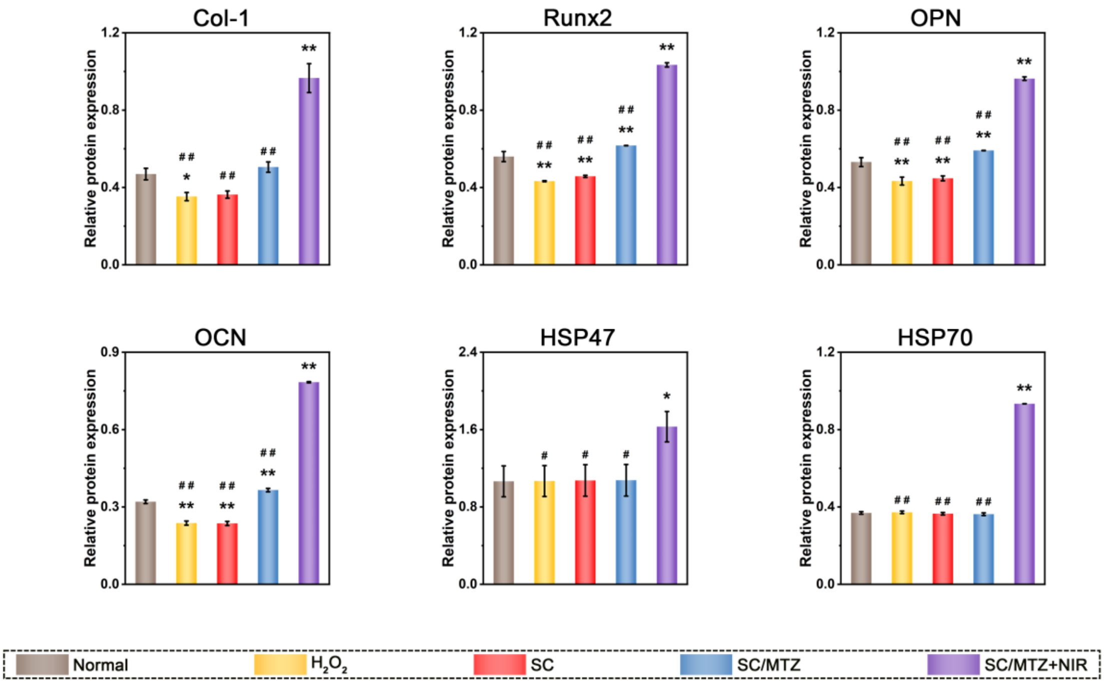

Figure S17. Quantitative analysis of osteogenesis-related protein expression in BMSCs after different treatments. Data are presented as the mean ± SD (n = 3). *P < 0.05 and **P < 0.01 indicate significant differences compared with the normal group. #P < 0.05 and ##P < 0.01 indicate significant differences compared with the SC/MTZ+NIR group.

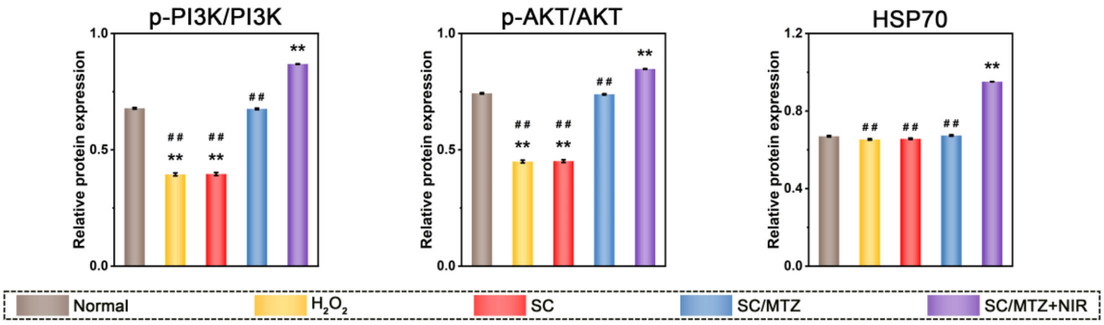

Figure S18. Quantitative analysis of PI3K/AKT signaling pathway-related proteins in BMSCs after different treatments. Data are presented as the mean ± SD (n = 3). *P < 0.05 and **P < 0.01 indicate significant differences compared with the normal group. #P < 0.05 and ##P < 0.01 indicate significant differences compared with the SC/MTZ+NIR group.

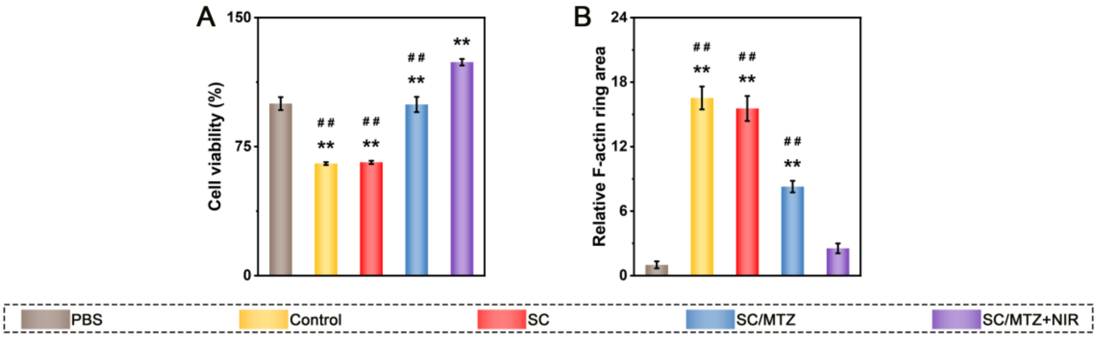

Figure S19. (A) CCK-8 assay of macrophages in each group. **(B)** Quantitative analysis of F-actin ring area in each group. Data are presented as the mean ± SD (n = 3). *P < 0.05 and **P < 0.01 indicate significant differences compared with the PBS group. $^{\#}$P < 0.05 and $^{\#\#}$P < 0.01 indicate significant differences compared with the SC/MTZ+NIR group.

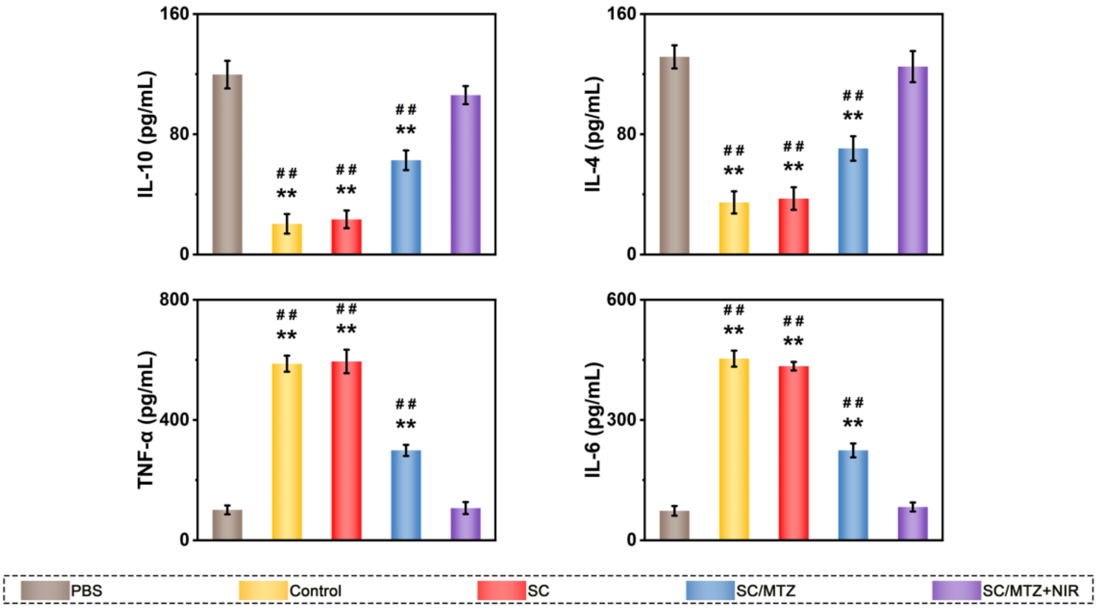

Figure S20. ELISA analysis of proinflammatory and anti-inflammatory cytokines (IL-10, IL-4, TNF-α, and IL-6) secreted by macrophages after various treatments. Data are presented as the mean ± SD (n = 3). *P < 0.05 and **P < 0.01 indicate significant differences compared with the PBS group. $^{\#}$P < 0.05 and $^{\#\#}$P < 0.01 indicate significant differences compared with the SC/MTZ+NIR group.

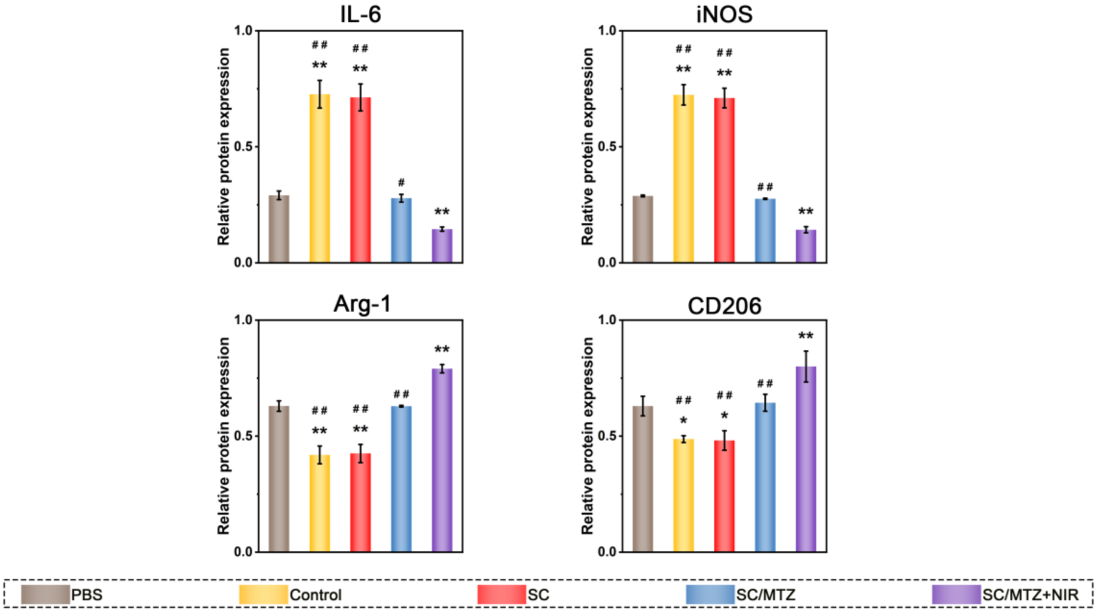

Figure S21. Quantitative analysis of inflammation-related protein expression in macrophages following different treatments. Data are presented as the mean ± SD (n = 3). *P < 0.05 and **P < 0.01 indicate significant differences compared with the PBS group. #P < 0.05 and # #P < 0.01 indicate significant differences compared with the SC/MTZ+NIR group.

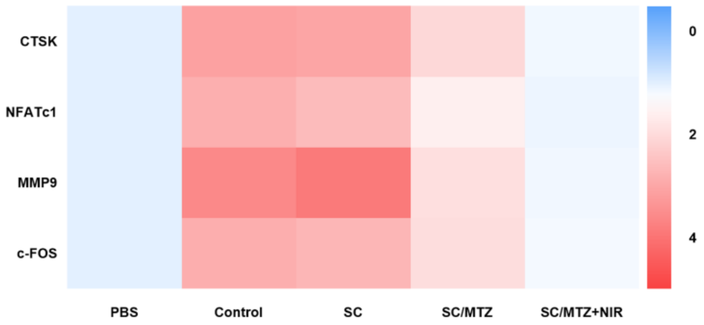

Figure S22. Heatmap of osteoclastogenesis-related marker gene expression.

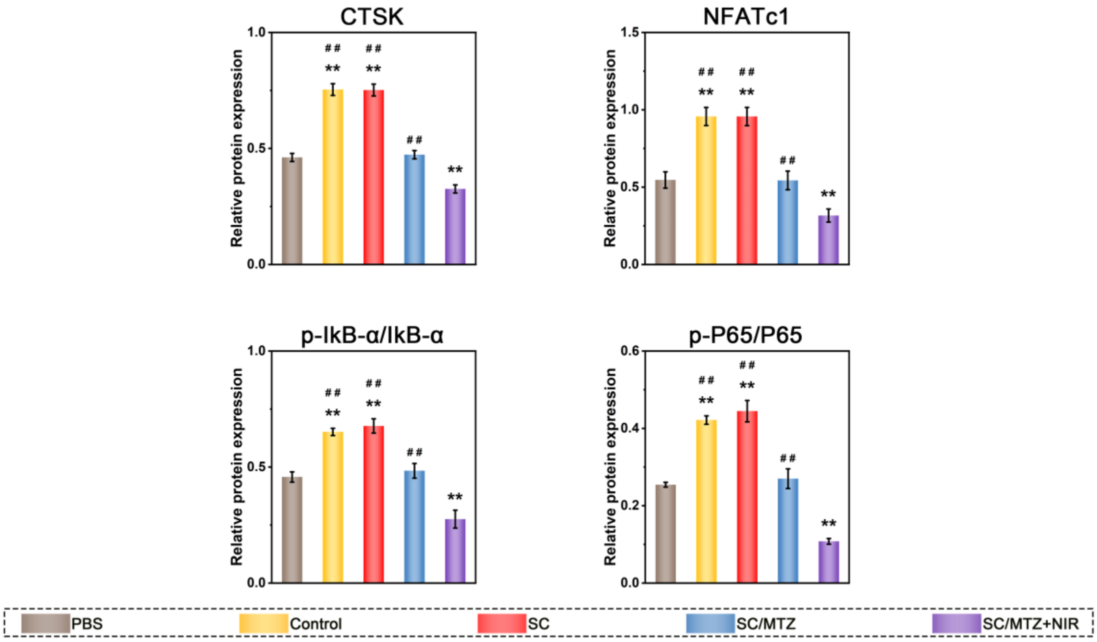

Figure S23. Quantitative analysis of osteoclastogenesis- and NF-κB pathway-related protein expression in BMMs after various treatments. Data are presented as the mean ± SD (n = 3). *P < 0.05 and **P < 0.01 indicate significant differences compared with the PBS group. #P < 0.05 and # #P < 0.01 indicate significant differences compared with the SC/MTZ+NIR group.

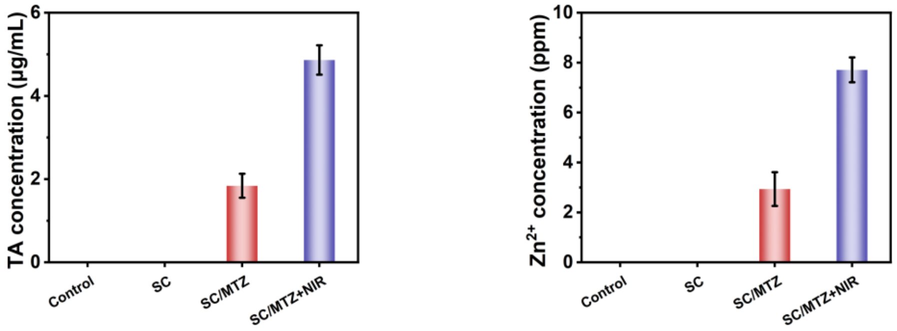

Figure S24. The amount of cumulative TA and Zn^{2+} release from different samples with or without the treatment of NIR. Data are presented as the mean ± SD (n = 3).

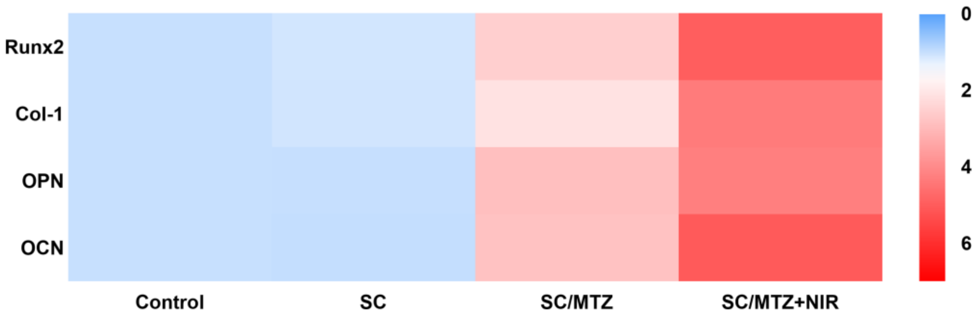

Figure S25. Relative mRNA expression of osteogenesis-related genes, including Col-1,

Runx2, OPN, and OCN. Data are presented as the mean ± SD (n = 3).

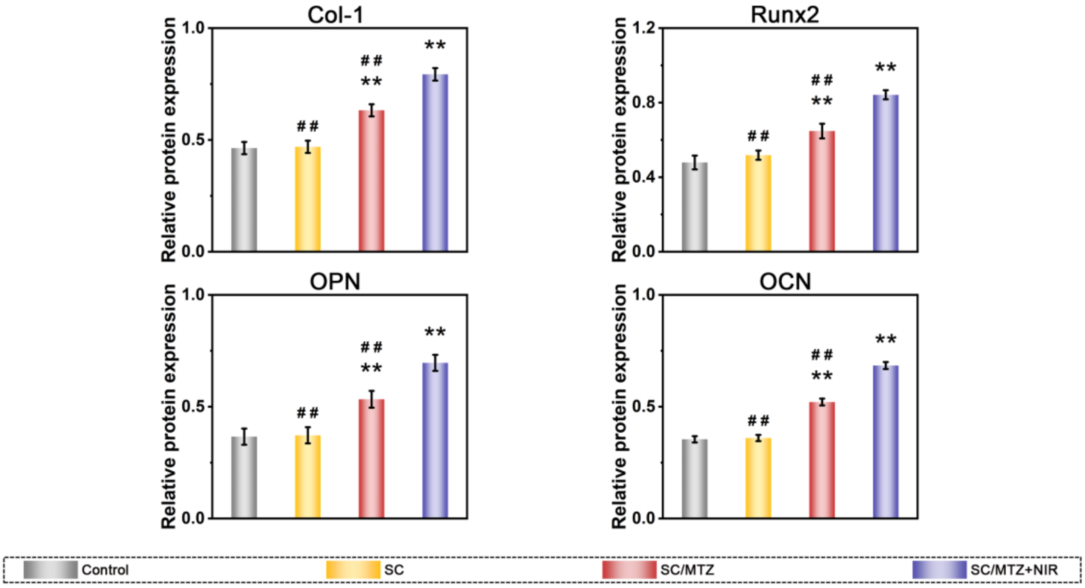

Figure S26. Quantitative analysis of osteogenic protein expression in BMSCs after various treatments. Data are presented as the mean ± SD (n = 3). *P < 0.05 and **P < 0.01 indicate significant differences compared with the control group. #P < 0.05 and # #P < 0.01 indicate significant differences compared with the SC/MTZ+NIR group.

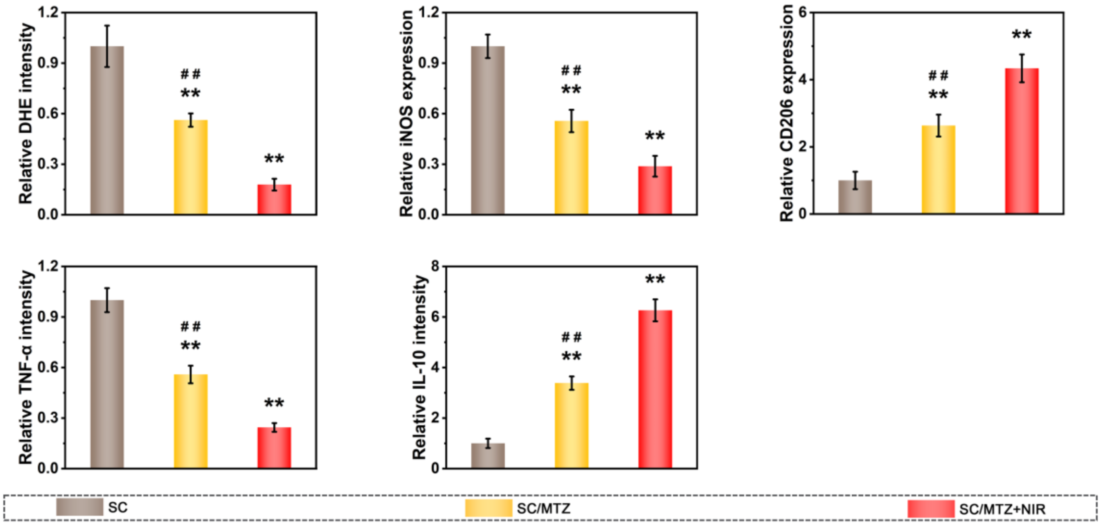

Figure S27. Quantitative analysis of DHE staining, immunohistochemical staining of iNOS and CD206, and immunofluorescence staining of TNF-α and IL-10 in each group. Data are presented as the mean ± SD (n = 3). *P < 0.05 and **P < 0.01 indicate significant differences compared with the SC group. #P < 0.05 and # #P < 0.01 indicate significant differences compared with the SC/MTZ+NIR group.

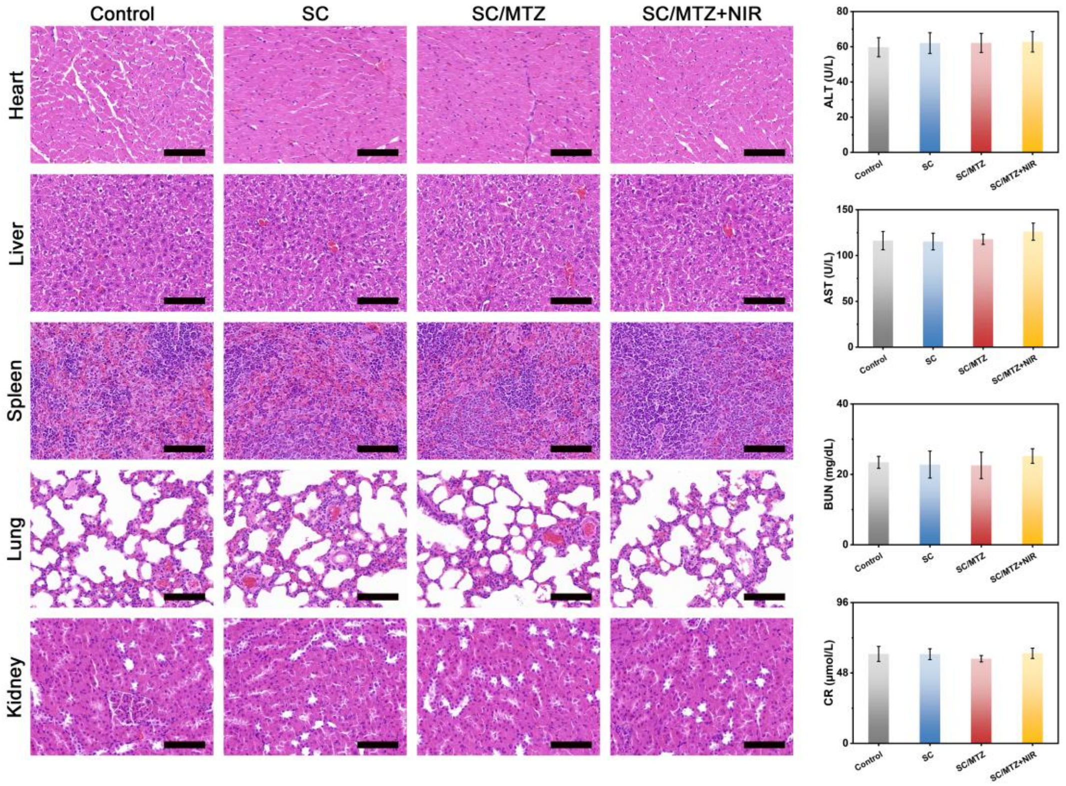

Figure S28. H&E staining and blood biochemical indicators of various organs in mice after implantation for 4 weeks. Scale bar: 100 μm. Data are presented as the mean ± SD (n = 3).

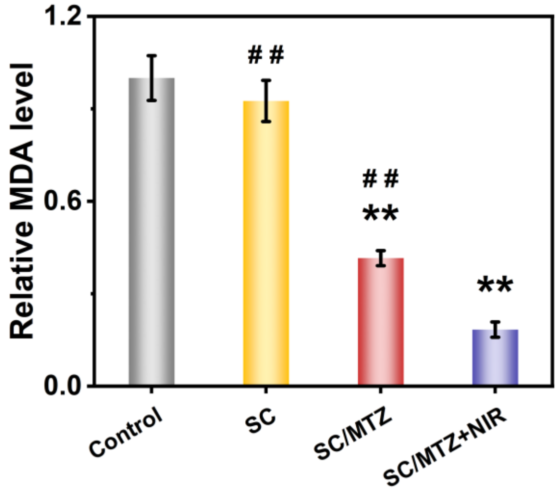

Figure S29. Quantitative analysis of the MDA levels in the different experimental groups. Data are presented as the mean ± SD (n = 3). *P < 0.05 and **P < 0.01 indicate significant differences compared with the control group. $^{\#}$P < 0.05 and $^{\#\#}$P < 0.01 indicate significant differences compared with the SC/MTZ+NIR group.

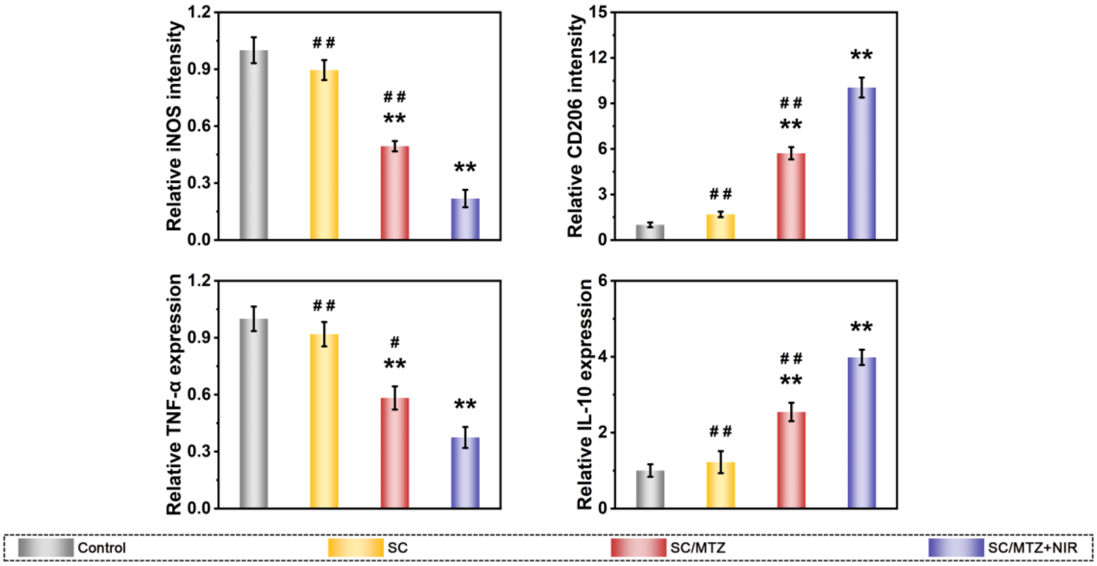

Figure S30. Quantitative analysis of immunofluorescence staining of iNOS and CD206 and immunohistochemical staining of TNF-α and IL-10 in each group. Data are presented as the mean ± SD (n = 3). *P < 0.05 and **P < 0.01 indicate significant differences compared with the control group. #P < 0.05 and ##P < 0.01 indicate significant differences compared with the SC/MTZ+NIR group.

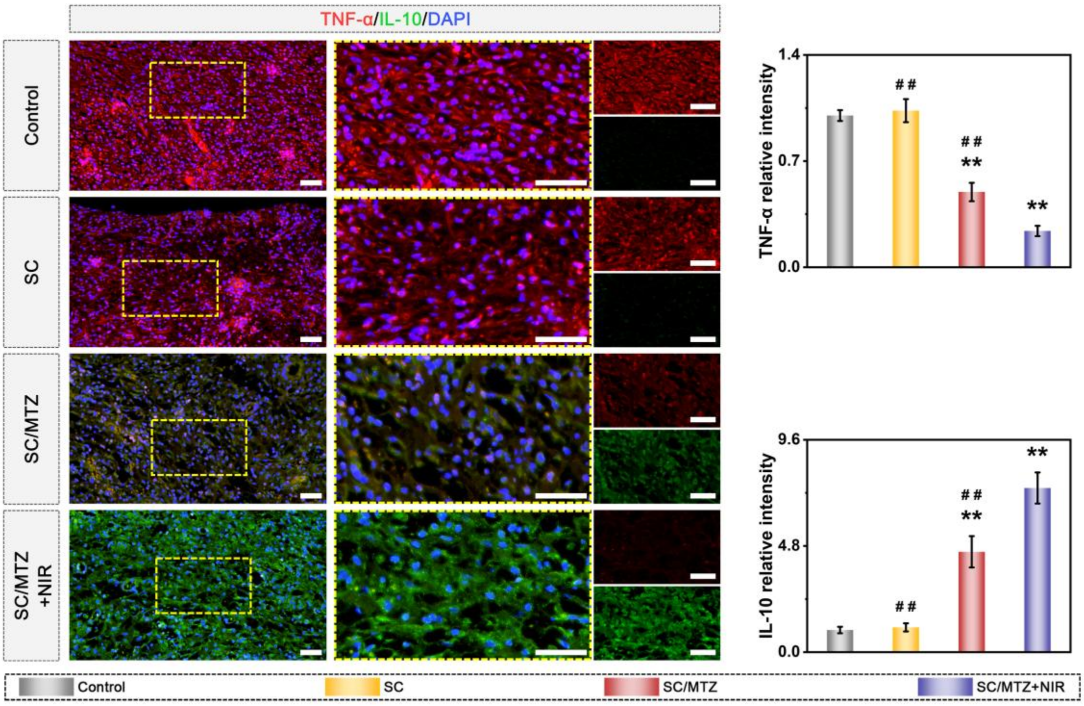

Figure S31. Representative images and corresponding quantitative analysis of the immunofluorescence staining of TNF-α and IL-10 in each group. Scale bar: 50 μm. Data are presented as the mean ± SD (n = 3). *P < 0.05 and **P < 0.01 indicate significant differences compared with the control group. #P < 0.05 and ##P < 0.01 indicate significant differences compared with the SC/MTZ+NIR group.

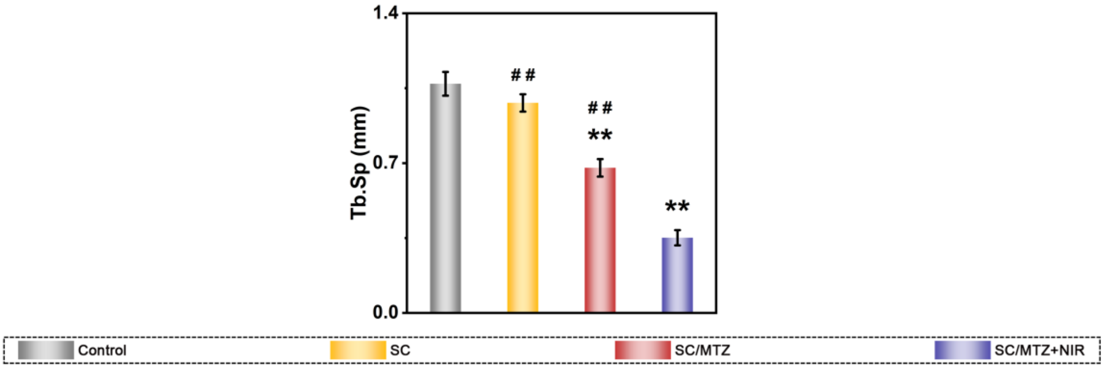

Figure S32. Quantitative statistics of new bone from micro-CT analysis. Data are presented as the mean ± SD (n = 3). *P < 0.05 and **P < 0.01 indicate significant differences compared with the control group. #P < 0.05 and ##P < 0.01 indicate significant differences compared with the SC/MTZ+NIR group.

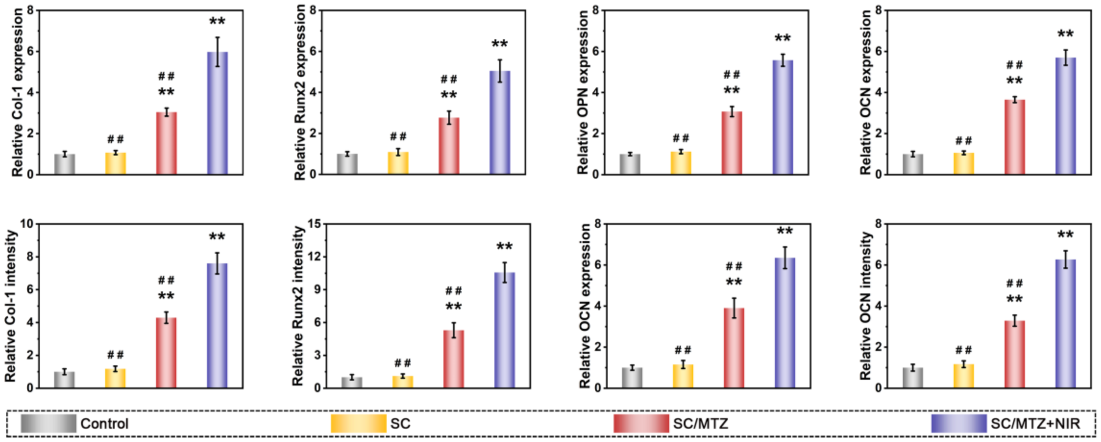

Figure S33. Quantitative analysis of immunohistochemical and immunofluorescence staining in each group. Data are presented as the mean ± SD (n = 3). *P < 0.05 and **P < 0.01 indicate significant differences compared with the control group. #P < 0.05 and ##P < 0.01 indicate significant differences compared with the SC/MTZ+NIR group.

Table S1. Primer sequences used in qRT-PCR analysis.

Genes	Primers (F, forward; R, reverse; 5'-3')
Mouse-GADPH	F: TCAACGGCACAGTCAAGG
	R: TTAGTGGGGTCTCGCTCC
Mouse-ALP	F: TGACTACCACTCGGGTGAACC
	R: TGATATGCGATGTCCTTGCAG
Mouse-Runx2	F: CATCCCAGTATGAGAGTAGGTGT
	R: GCTCAGATAGGAGGGGTAAGAC
Mouse-Col-1	F: CTGACTGGAAGAGCGGAGAG

	R: CGGCTGAGTAGGGAACACAC
Mouse-OPN	F: TCTGAGGGACTAACTACGACCAT<br>R: TGGAAGAGTTTCTTGCTTAAAGTC
Mouse-OCN	F: TTCTGCTCACTCTGCTGACCC<br>R: CTGATAGCTCGTCACAAGCAGG
Mouse-TNF-α	F: CAGGCGGTGCCTATGTCTC<br>R: CGATCACCCCGAAGTTCAGTAG
Mouse-IL-6	F: GAGACCACTGGGGAGAATGC<br>R: TTGCCAGGTGGGTAAAGTGG
Mouse-iNOS	F: GAATCTTGGAGCGAGTTG<br>R: CCAGGAAGTAGGTGAGGG
Mouse-CD86	F: ATGGGCTCGTATGATTGT<br>R: TCTTAGGTTTCGGGTGAC
Mouse-TGF-β	F: ACCGCAACAACGCCATCT<br>R: GGGCACTGCTTCCCGAAT
Mouse-IL-10	F: TTTCAAACAAAGGACCAG<br>R: GGATCATTTCCGATAAGG
Mouse-Arg-1	F: AAGACAGCAGAGGAGGTG<br>R: AGTCAGTCCCTGGCTTA
Mouse-CD206	F: GCAAGTGATTTGGAGGCT<br>R: ATAGGAAACGGGAGAACC
Mouse-SOD2	F: CAGACCTGCCTTACGACTATGG<br>R: CTCGGTGGCGTTGAGATTGTT
Mouse-CAT	F: AGCGACCAGATGAAGCAGTG<br>R: TCCGCTCTCTGTCAAAGTGTG
Mouse-CTSK	F: GCACCCTTAGTCTTCCGCTC<br>R: GGTCATATAGCCGCCTCCAC
Mouse-NFATc1	F: TATATGAGCCCATCCTTGCCT<br>R: GGCTGCCTTCCGTCTCATAG
Mouse-c-FOS	F: TTGAGCGATCATCCCGGTC<br>R: GCGTGAGTCCATACTGGCAAG
Mouse-MMP9	F: CTGGACAGCCAGACACTAAAG<br>R: CTCGCGGCAAGTCTTCAGAG
Rat-GAPDH	F: CTCCCATTCTTCCACCTTTG<br>R: TGGTCCAGGGTTTCTTACT
Rat-ALP	F: GTAGCACCCCTTCTTCCGTC<br>R: GGGTTCACTCATGGAGGGTG
Rat-BMP-2	F: GGACCCGCTGTCTTCTAGT<br>R: ACAGGTCGAGCATATAGGGG
Rat- HIF-1α	F: GTCTAGGGATGCAGCACGAT<br>R: GGGGAAGTGGCAACTGATGA
Rat-VEGF	F: GCTGCAATGATGAAGCCCTG<br>R: TACACGTCTGCGGATCTTGG

Human-GAPDH	F: CATCATCCCTGCCTCTACTGG<br>R: GTGGGTGTCGCTGTTGAAGTC
Human-VEGF	F: TATGCGGATCAAACCTCACCA<br>R: CACAGGGATTTTTCTTGTCTTGCT
Human-HIF-1α	F: ATCCATGTGACCATGAGGAAAT<br>R: CTCGGCTAGTTAGGGTACACTT
Human-CD31	F: AACAGTGTTGACATGAAGAGCC<br>R: TGTAAAACAGCACGTCATCCTT
Human-eNOS	F: ATGTTTGTCTGCGGCGATGT<br>R: GTGCGTATGCGGCTTGTC

Movie S1. The SC/MTZ hydrogel can maintain stable adhesion under continuous water flow washing.
